# Supplementary material for: Evaluating use of mass-media communication intervention ‘MTV-Shuga’ on increased awareness and demand for HIV and sexual health services by adolescent girls and young women in South Africa: an observational study
Source: BMJ Open. 2023 May 18;13(5):e062804. doi: 10.1136/bmjopen-2022-062804 (PMC10201230; doi:10.1136/bmjopen-2022-062804)
Supplement: Supplementary data [file bmjopen-2022-062804supp001.pdf]

Supplementary Table 1 Exposure to MTV Shuga and PrEP awareness in the nested cohort of AGYW aged 13-22 (n=2184)

	Unadjusted		Shuga adjusted			Adjusted-All		
	**OR**	**95%CI**	**OR**	**95%CI**	**p-value**	**OR**	**95%CI**	**p-value**
**Ever watched MTV Shuga, 2018/19**								
No	1					1		
Yes	2.01	1.55 -2.62				2.06	1.57 -2.70	<0.001
**Age**	1.12	1.08-1.16	1.12	1.08-1.17	<0.001	1.11	1.06-1.17	<0.001
**Currently in school**								
No	1		1			1		
Yes	0.6	0.47 -0.76	0.59	0.46 -0.75	<0.001	0.89	0.65 -1.23	0.488
**Socio-economic status, 2018**								
Low	1		1			1		
Middle	0.99	0.71 -1.39	0.96	0.68 -1.35		1.01	0.71 -1.44	
High	1.13	0.81 -1.58	1.05	0.75 -1.47	0.744	1.11	0.78 -1.58	0.699
**Urban or rural**								
Rural	1		1			1		
Peri-urban/urban	0.98	0.79 -1.22	0.93	0.75 -1.16	0.525	0.92	0.73 -1.15	0.459
**Invited or received DREAMS, 2017/18**								
No	1		1			1		
Yes	0.89	0.73 -1.10	0.88	0.71 -1.08	0.212	0.99	0.79 -1.23	0.897
