# Supplementary material for: Evaluating use of mass-media communication intervention ‘MTV-Shuga’ on increased awareness and demand for HIV and sexual health services by adolescent girls and young women in South Africa: an observational study
Source: BMJ Open. 2023 May 18;13(5):e062804. doi: 10.1136/bmjopen-2022-062804 (PMC10201230; doi:10.1136/bmjopen-2022-062804)
Supplement: Supplementary data [file bmjopen-2022-062804supp002.pdf]

Supplementary Table 2 Exposure to MTV Shuga and contraception uptake in the nested cohort of AGYW aged 13-22 (n=2184)

	Unadjusted		Shuga adjusted			Adjusted-All		
	OR	95%CI	OR	95%CI	p-value	OR	95%CI	p-value
**Ever watched MTV Shuga, 2018/19**								
No	1					1		
Yes	1.92	1.37 -2.68				2.08	1.45-2.98	<0.001
**Age**	1.26	1.19-1.33	1.27	1.20-1.34	<0.001	1.32	1.24-1.42	<0.001
**Currently in school**								
No	1		1			1		
Yes	0.6	0.44 -0.83	0.59	0.43-0.82	0.001	1.7	1.13-2.55	0.01
**Socio-economic status, 2018**								
Low	1		1			1		
Middle	0.79	0.51 -1.22	0.76	0.49-1.19		0.8	0.51-1.28	
High	0.86	0.55 -1.34	0.8	0.51-1.24	0.489	0.83	0.52-1.32	0.646
**Urban or rural**								
Rural	1		1			1		
Peri-urban/urban	0.91	0.68 -1.24	0.86	0.64-1.17	0.345	0.89	0.64-1.24	0.49
**Invited or received DREAMS, 2017/18**								
No	1		1			1		
Yes	0.84	0.63 -1.11	0.83	0.62-1.10	0.191	1.04	0.76-1.42	0.813
