# Supplementary material for: Evaluating use of mass-media communication intervention ‘MTV-Shuga’ on increased awareness and demand for HIV and sexual health services by adolescent girls and young women in South Africa: an observational study
Source: BMJ Open. 2023 May 18;13(5):e062804. doi: 10.1136/bmjopen-2022-062804 (PMC10201230; doi:10.1136/bmjopen-2022-062804)
Supplement: Supplementary data [file bmjopen-2022-062804supp003.pdf]

Supplementary Table 3 Exposure to MTV Shuga and consistent condom use in the nested cohort of AGYW aged 13-22 (n=2184)

	Unadjusted		Shuga adjusted			Adjusted-All		
	OR	95%CI	OR	95%CI	p-value	OR	95%CI	p-value
**Ever watched MTV Shuga, 2018/19**								
No	1					1		
Yes	1.84	1.22-2.78				1.9	1.24-2.93	0.003
**Age**	0.94	0.86-1.01	0.94	0.87-1.02	0.157	1	0.91-1.10	0.984
**Currently in school**								
No	1		1			1		
Yes	1.76	1.22-2.53	1.73	1.20-2.49	0.003	1.78	1.15-2.76	0.01
**Socio-economic status, 2018**								
Low	1		1			1		
Middle	1.01	0.60-1.70	0.97	0.58-1.63		0.98	0.58-1.67	
High	0.87	0.52-1.47	0.8	0.47-1.36	0.552	0.86	0.50-1.48	0.753
**Urban or rural**								
Rural	1		1			1		
Peri-urban/urban	0.7	0.48-1.02	0.67	0.46-0.97	0.035	0.71	0.48-1.05	0.088
**Invited or received DREAMS, 2017/18**								
No	1		1			1		
Yes	1.13	0.81-1.59	1.13	0.80-1.59	0.486	0.97	0.67-1.40	0.875
