# Supplementary material for: Evaluating use of mass-media communication intervention ‘MTV-Shuga’ on increased awareness and demand for HIV and sexual health services by adolescent girls and young women in South Africa: an observational study
Source: BMJ Open. 2023 May 18;13(5):e062804. doi: 10.1136/bmjopen-2022-062804 (PMC10201230; doi:10.1136/bmjopen-2022-062804)
Supplement: Supplementary data [file bmjopen-2022-062804supp004.pdf]

Supplementary Table 4 Exposure to MTV Shuga and HIV testing in the nested cohort of AGYW aged 13-22 (n=2184)

	Unadjusted		Shuga adjusted			Adjusted-All		
	OR	95%CI	OR	95%CI	p-value	OR	95%CI	p-value
**Ever watched MTV Shuga, 2018/19**								
No	1					1		
Yes	0.93	0.71-1.21				1.02	0.77-1.36	0.885
**Age**	1.3	1.25-1.35	1.3	1.24-1.35	<0.001	1.3	1.24-1.37	<0.001
**Currently in school**								
No	1		1			1		
Yes	0.37	0.28-0.48	0.37	0.28-0.48	0	1.07	0.74-1.53	0.726
**Socio-economic status, 2018**								
Low	1		1			1		
Middle	0.67	0.48-0.94	0.67	0.48-0.95		0.71	0.49-1.02	
High	0.58	0.41-0.81	0.58	0.41-0.81	0.007	0.6	0.41-0.86	0.018
**Urban or rural**								
Rural	1		1			1		
Peri-urban/urban	0.84	0.68-1.03	0.84	0.69-1.04	0.104	0.89	0.71-1.11	0.298
**Invited or received DREAMS, 2017/18**								
No	1		1			1		
Yes	0.84	0.69-1.02	0.84	0.69-1.02	0.081	0.98	0.79-1.23	0.89
