# Supplementary material for: Evaluating use of mass-media communication intervention ‘MTV-Shuga’ on increased awareness and demand for HIV and sexual health services by adolescent girls and young women in South Africa: an observational study
Source: BMJ Open. 2023 May 18;13(5):e062804. doi: 10.1136/bmjopen-2022-062804 (PMC10201230; doi:10.1136/bmjopen-2022-062804)
Supplement: Supplementary data [file bmjopen-2022-062804supp005.pdf]

Supplementary Table 5: Exposure to MTV Shuga with HIV prevention and SRHR awareness and uptake in the cross-sectional analysis of AGYW aged 12-24 (n=4127)

	aOR (95% Confidence Interval)	P-value
**District**		
City of Johannesburg	1	
Ekurhuleni	0.66 (0.47-0.91)	0.013
eThekwini	0.78 (0.54-1.13)	0.186
**Age group**		
12-14	1	
15-19	1.75 (0.35-8.84)	0.499
20-24	1.60 (0.32-8.07)	0.570
**Highest education level**		
Grade R to 7	1	
No schooling	3.84 (1.05-14.02)	0.041
Grade 8 to 12	3.17 (0.93-10.77)	0.065
Complete or incomplete tertiary	6.52 (1.90-22.33)	0.003
**Ever had sex with a boy/man**		
No	1	
Yes	0.67 (0.32-1.40)	0.286
**Away from home**		
No	1	
Yes	1.48 (0.93-2.37)	0.099
**PrEP awareness**		
No	1	
Yes	1.71 (1.20-2.43)	0.003
**Contraception use**		
No	1	
Yes	0.95 (0.74-1.23)	0.718
**Condom use at last sex**		
No	1	
Yes	1.19 (0.92-1.55)	0.183
**HIV test (self-report)**		
No	1	
Yes	0.88 (0.63-1.24)	0.471
