# Supplementary material for: Evaluating use of mass-media communication intervention ‘MTV-Shuga’ on increased awareness and demand for HIV and sexual health services by adolescent girls and young women in South Africa: an observational study
Source: BMJ Open. 2023 May 18;13(5):e062804. doi: 10.1136/bmjopen-2022-062804 (PMC10201230; doi:10.1136/bmjopen-2022-062804)
Supplement: Supplementary data [file bmjopen-2022-062804supp006.pdf]

Supplementary Table 6 Estimated causal effect of MTV Shuga on HSV-2 incidence, overall and by age group

**Estimated causal effect of MTV Shuga on HSV-2 incidence, overall and by age group**												
	**% Outcome in total study population**	**Estimated % Outcome if no Shuga**	**95% CI**	**Estimated % Outcome if all get Shuga**	**95% CI**	**Risk Difference (%; PS adjusted)**	**95% CI**	**Prevalence Ratio (%; PS adjusted)**	**95% CI**	**Odds Ratio (%; PS adjusted)**	**95% CI**
<u>**PS adjustment: Primary results**</u>											
Overall	17.3	17.2	14.71 - 19.67	18.0	14.51 - 22.32	0.80	-3.24 - 5.71	1.05	0.82 - 1.36	1.06	0.79 - 1.46
13-17 Years	13.2	12.4	9.85 - 15.16	15.7	11.63 - 20.26	3.34	-1.60 - 8.25	1.27	0.89 - 1.79	1.32	0.87 - 1.98
18-22 Years	24.3	25.8	21.01 - 30.74	22.0	15.19 - 30.17	-3.76	-11.92 - 5.68	0.85	0.59 - 1.24	0.81	0.51 - 1.34
**Sensitivity analyses**											
<u>Regression under counterfactual framework</u>											
Overall	17.3	17.2	14.74 - 19.66	17.8	14.50 - 22.08	0.65	-3.42 - 5.72	1.04	0.82 - 1.36	1.05	0.78 - 1.46
13-17 Years	13.2	12.42	9.86 - 15.21	15.9	11.85 - 20.49	3.47	-1.54 - 8.63	1.28	0.89 - 1.80	1.33	0.88 - 1.99
18-22 Years	24.3	25.7	20.78 - 30.63	21.3	14.88 - 28.28	-4.42	-12.28 - 4.95	0.83	0.57 - 1.22	0.78	0.48 - 1.31
<u>PS stratification</u>											
Overall	17.3	17.1	14.68 - 19.68	17.9	14.53 - 22.13	0.80	-3.21 - 5.52	1.09	0.84 - 1.40	1.11	0.82 - 1.51
<u>PS weighting</u>											

Overall	17.3	17.08	14.74 - 19.48	17.22	13.99 - 21.10	0.13	-3.76 - 4.63	1.01	0.79 - 1.30	1.01	0.76 - 1.37
