# Supplementary material for: Evaluating use of mass-media communication intervention ‘MTV-Shuga’ on increased awareness and demand for HIV and sexual health services by adolescent girls and young women in South Africa: an observational study
Source: BMJ Open. 2023 May 18;13(5):e062804. doi: 10.1136/bmjopen-2022-062804 (PMC10201230; doi:10.1136/bmjopen-2022-062804)
Supplement: Author's manuscript [file bmjopen-2022-062804.draft_revisions.pdf]

BMJ Open

BMJ Open is committed to open peer review. As part of this commitment we make the peer review history of every article we publish publicly available.

When an article is published we post the peer reviewers' comments and the authors' responses online. We also post the versions of the paper that were used during peer review. These are the versions that the peer review comments apply to.

The versions of the paper that follow are the versions that were submitted during the peer review process. They are not the versions of record or the final published versions. They should not be cited or distributed as the published version of this manuscript.

BMJ Open is an open access journal and the full, final, typeset and author-corrected version of record of the manuscript is available on our site with no access controls, subscription charges or pay-per-view fees (http://bmjopen.bmj.com).

If you have any questions on BMJ Open's open peer review process please email info.bmjopen@bmj.com

BMJ Open

‘MTV Shuga’: Mass media communication in adolescent girls and young women in South Africa: Can it increase awareness and demand for HIV and sexual health technologies

Journal:	*BMJ Open*
Manuscript ID	bmjopen-2022-062804
Article Type:	Original research
Date Submitted by the Author:	28-Mar-2022
Complete List of Authors:	Chimbindi, Natsayi; Africa Health Research Institute; University College London,<br>Mthiyane, Nondumiso; Africa Health Research Institute<br>Chidumwa, Glory ; Africa Health Research Institute<br>Zuma, Thembelihle; Africa Health Research Institute; University College London,<br>Dreyer, Jaco; Africa Health Research Institute<br>Birdthistle, Isolde; London School of Hygiene and Tropical Medicine Faculty of Epidemiology and Population Health, Department of Population Health<br>Floyd, Sian; London School of Hygiene & Tropical Medicine, Infectious Disease Epidemiology<br>Kyegombe, Nambusi; London School of Hygiene & Tropical Medicine<br>Grundy, Chris; London School of Hygiene and Tropical Medicine Faculty of Epidemiology and Population Health, Department of Population Health<br>Cawood, Cherie; Epicentre Health Research<br>Danaviah, Siva; Africa Health Research Institute<br>Smit, Theresa; Africa Health Research Institute<br>Pillay, Deenan; University College London,<br>Baisley, Kathy; London School of Hygiene and Tropical Medicine Faculty of Epidemiology and Population Health; Africa Health Research Institute<br>Harling, Guy; Africa Health Research Institute<br>Seeley, Janet; London School of Hygiene and Tropical Medicine, Department of Global Health &Development; Africa Health Research Institute<br>Shahmanesh, Maryam; University College London, Institute for Global Health; Africa Health Research Institute,
Keywords:	HIV & AIDS < INFECTIOUS DISEASES, PUBLIC HEALTH, PREVENTIVE MEDICINE

SCHOLARONE™
Manuscripts

I, the Submitting Author has the right to grant and does grant on behalf of all authors of the Work (as defined in the below author licence), an exclusive licence and/or a non-exclusive licence for contributions from authors who are: i) UK Crown employees; ii) where BMJ has agreed a CC-BY licence shall apply, and/or iii) in accordance with the terms applicable for US Federal Government officers or employees acting as part of their official duties; on a worldwide, perpetual, irrevocable, royalty-free basis to BMJ Publishing Group Ltd ("BMJ") its licensees and where the relevant Journal is co-owned by BMJ to the co-owners of the Journal, to publish the Work in this journal and any other BMJ products and to exploit all rights, as set out in our licence.

The Submitting Author accepts and understands that any supply made under these terms is made by BMJ to the Submitting Author unless you are acting as an employee on behalf of your employer or a postgraduate student of an affiliated institution which is paying any applicable article publishing charge ("APC") for Open Access articles. Where the Submitting Author wishes to make the Work available on an Open Access basis (and intends to pay the relevant APC), the terms of reuse of such Open Access shall be governed by a Creative Commons licence – details of these licences and which Creative Commons licence will apply to this Work are set out in our licence referred to above.

Other than as permitted in any relevant BMJ Author's Self Archiving Policies, I confirm this Work has not been accepted for publication elsewhere, is not being considered for publication elsewhere and does not duplicate material already published. I confirm all authors consent to publication of this Work and authorise the granting of this licence.

For peer review only

Title page

‘MTV Shuga’: Mass media communication in adolescent girls and young women in South Africa: Can it increase awareness and demand for HIV and sexual health technologies

Authors: Natsayi Chimbindi*[1,2,3], Nondumiso Mthiyane*[2], Glory Chidumwa[1], Thembelihle Zuma[1,2,3], Jaco Dreyer[1], Isolde Birdthistle[4], Sian Floyd[4], Nambusi Kyegombe[4], Chris Grundy[4], Cherie Cawood[5], Siva Danaviah[1], Theresa Smit[1], Deenan Pillay[2], Kathy Baisley[1,3], Guy Harling[1,2,3,6,7], Janet Seeley[1,3,4], Maryam Shahmanesh§[1,2,3]

1. Africa Health Research Institute, KwaZulu-Natal, South Africa
2. University College London, Institute for Global Health, London, UK
3. University of KwaZulu-Natal, KwaZulu-Natal, South Africa
4. London School of Hygiene and Tropical Medicine, London, UK
5. Epicentre, South Africa
6. MRC/Wits Rural Public Health & Health Transitions Research Unit (Agincourt), University of the Witwatersrand, Gauteng, South Africa
7. Department of Epidemiology & Harvard Center for Population and Development Studies, Harvard T.H. Chan School of Public Health, Boston, USA

*Joint authors

§Corresponding author (full address details): Maryam Shahmanesh, Institute for Global Health, UCL, 3rd Floor, Mortimer Market Centre, Capper Street, London WC1E 6JB. m.shahmanesh@ucl.ac.uk. +44 7776 185 572

Key words (6): HIV, Adolescents, mass communication campaigns, Sexual Health, Teenage Pregnancy, South Africa.

For peer review only - http://bmjopen.bmj.com/site/about/guidelines.xhtml

Abstract (word count=300)

Objectives: Mass media edu-dramas have potential to increase demand for HIV and sexual and reproductive health(SRH) services among young people. We investigate the effect of exposure to MTV Shuga Down South(DS) during the scale-up of combination HIV-prevention interventions (DREAMS) on awareness and uptake of SRH and HIV-prevention services by adolescent girls and young women(AGYW).

Design: One longitudinal and three cross-sectional surveys of representative samples of AGYW

Setting: AGYW in four South African districts with high HIV-prevalence (>10%) (May 2017 and September 2019)

Participants: 6341 AGYW aged 12-24

Measures: Using logistic regression we measured the relationship between exposure to MTV Shuga-DS and awareness of Pre-Exposure Prophylaxis (PrEP), condom-use at last sex, uptake of HIV-testing or contraception, and incident pregnancy or HSV-2 infection.

Results: Within the rural cohort 2184 (85.5%) of eligible sampled individuals were enrolled, of whom 92.6% had at least one follow-up visit; the urban cross-sectional surveys enrolled 4157 (22.6%) of eligible sampled individuals. Self-report of watching at least one MTV Shuga-DS episode was 14.1% (cohort) and 35.8% (cross-section), while storyline recall was 5.5% (cohort) and 6.7% (cross-section).

In the cohort, after adjustment (for DREAMS-exposure, age, education, SES), MTV Shuga-DS exposure was associated with increased PrEP awareness (aOR=2.06, 95%CI:1.57-2.70), contraception uptake (aOR=2.08, 95%CI:1.45-2.98) and consistent condom-use (aOR=1.84, 95%CI:1.24-2.93), but not with HIV-testing (aOR=1.02,95%CI: 0.77-1.21) or acquiring HSV-2 (aOR=0.92, 95%CI: 0.61-1.38). In the cross-sections, MTV Shuga-DS was associated with greater PrEP awareness (aOR=1.7, 95%CI: 1.20-2.43), but no other outcome.

Conclusions: Among both urban and rural AGYW in South Africa, MTV Shuga-DS exposure was associated with increased PrEP awareness and improved demand for some HIV-prevention and SRH technologies but not sexual health outcomes. However, exposure to MTV Shuga-DS was low. Given these positive indications, supportive programming may be required to raise exposure and allow future evaluation of edu-drama impact in this setting.

For peer review only

Strengths and limitations of this study

- We use the opportunity of MTV Shuga being broadcast in the context of an impact evaluation of DREAMS roll out (January 2016-September 2019) in a representative, population-based sample of young people to describe the real-world reach of nationally broadcast MTV Shuga-DS.

- We employed a longitudinal cohort (n~2000) and cross-sectional surveys (n~5000) of representative samples of AGYW aged 12-24 in four districts of South Africa with a high burden of HIV to measure the reach of MTV Shuga-DS.

- However, as this is an observational study, we cannot exclude the possibility that those who are exposed to MTV Shuga are systematically different in ways that impact on the outcome of interest, for example more exposed to social media and sexual health promotion and innovative technologies to support sexual health than those who were not.

For peer review only

For peer review only - http://bmjopen.bmj.com/site/about/guidelines.xhtml

[word count 4531]

Introduction

HIV remains one of the gravest health problems facing young people living in sub-Saharan Africa (SSA). There are over 7.6 million people living with HIV in South Africa, with 200,000 new HIV infections annually in those aged 15-49 years (1). The highest incidence is in adolescent girls and young women (AGYW) (15-24 years) (1, 2). In response to this the South African government launched the `She Conquers Campaign', and the US President's Emergency Fund for AIDS Relief and others are supporting the roll-out of Determined, Resilient, Empowered, AIDS free, Mentored, and Safe (DREAMS)(3-5). These programmes provide an evidence-based combination HIV prevention package, including HIV-testing and counselling for adolescent girls and young women (AGYW) and male partners, alongside universal test and treat and improved sexual health services (6, 7).

However, the key ingredient to success of these multicomponent interventions will be the extent to which AGYW and their male partners at most risk of HIV will uptake and adhere to the active components of the intervention. This is challenging: uptake and retention of contraception and HIV treatment cascade by young people, even within population-wide Universal Test and Treat trials, has been suboptimal (2, 8, 9). Data from the baseline analysis for the DREAMS impact evaluation in uMkhanyakude district, rural KwaZulu-Natal (KZN) in 2015 suggest that less than 40% of girls (15-19 years) and boys and young men (15-29 years) had ever tested for HIV; linkage to HIV treatment was even lower (10). Contraception use prevalence was 20% in girls (15-19 years) and 50% in young women (20-24 years) and 21% of 15-19 year old girls had ever been pregnant (2).

It is against this backdrop that the fifth series of Shuga: 'MTV Shuga: Down South' (MTV Shuga-DS), a mass media serial edu-drama designed for SA, was broadcast on free-to-air South Africa National television. From March 8th 2017, MTV Shuga aired one episode per week for 12 weeks (with repeats). MTV Shuga is a mass media behaviour change campaign that aims to improve sexual and reproductive health rights (SRHR). At the centre of the campaign, which includes radio and social media, is a TV-drama that weaves messages about HIV, family planning, transactional and intergenerational sex, safer and healthy sexual relationships, into storylines with young characters (http://www.mtvshuga.com/show/series-5/MTV Shuga-down-south/).

Mass media campaigns, have the potential to reach a large number of people and have been shown to improve knowledge and health behaviour of a range of health conditions, with more recent data suggesting that theoretically informed and targeted interventions are more likely

For peer review only - http://bmjopen.bmj.com/site/about/guidelines.xhtml

to have an effect (11, 12). MTV Shuga-DS was designed to reduce HIV-related risk behaviour and improve SRHR outcomes in adolescents and young adults in SA. This was expected to be achieved through increasing young people's demand for, and uptake of HIV and SRH prevention and treatment technologies. The show's characters explicitly model how to discuss issues that are sensitive or taboo. Moreover, in 'melodramas', tensions between good and evil are played out through 'good' characters who adopt certain behaviours, and 'evil' characters who reject them. The former are rewarded while the latter punished. A third category of character, the 'transitional' character, begins as ambivalent but changes into a positive role model and promote positive behaviour change. This is a deliberate method to immerse the audience in the action, rather than passively watching or listening (13). Young people, or at least early adopters, are anticipated to be immersed in the serial, able to classify and identify with the transitional characters and their outcomes. Pathways to behaviour change through MTV Shuga, therefore relate to the extent to which the observer, including early adopters, are immersed and critically engaged with the story. It also depends on a context which is supportive rather than disruptive (see the conceptual framework Figure 1 below) (14).

Figure 1 showing conceptual framework for MTV Shuga impact on HIV prevention on AGYW in uMkhanyakude

A cluster randomised controlled trial of community-viewings of MTV Shuga in Nigeria found that exposure to MTV Shuga significantly improved HIV knowledge and testing in both sexes, the intervention arm showing 35% more likely to test for HIV than the control arm. There was also a 60% reduction in genital chlamydia as a marker of recent sexual risk in women, amongst those exposed to MTV Shuga compared to those who were not (15). There were, however, fewer changes in social norms, particularly around gender-based violence. Further work suggested that the impact was greatest in those who were immersed in the narrative. The importance of immersion (classification of characters and identifying with them and observing outcomes) coupled with critical participation and an enabling context were also found in a thematic analysis looking at how storylines in MTV Shuga-DS shaped awareness, knowledge and opinions of sexual health and personal relationships among young people in SA (14).

Whilst the Nigerian and SA studies provide evidence for the efficacy of MTV Shuga impacting on SRHR and HIV-testing behaviours in exposed individuals, there is little evidence of how this will translate into a population-level effect when nationally broadcast and in less controlled environments. In particular, it is not clear how the impact will spill-over to non-viewers and how innovations will diffuse when shown and watched by adolescents and young adults in a real-world scale-up. It is also unclear how such impact will differ according to: setting (rural or

For peer review only - http://bmjopen.bmj.com/site/about/guidelines.xhtml

urban); differential digital literacy and access to social media (geographically and socioeconomically), and the dose and context of watching (shared viewing with family, friends or individual experience through social media).

We use the opportunity of MTV Shuga being broadcast in the context of an impact evaluation of DREAMS roll out (January 2016-September 2019) in a representative, population-based sample of young people (10) to describe the real-world reach of nationally broadcast MTV Shuga-DS. Further, we explore the hypothesis that exposure to a mass-media serial edu-drama, like MTV Shuga, will improve SRHR outcomes by increasing demand for, and uptake of, existing combination individual and community-based SRH and HIV prevention services for AGYW in four diverse settings including uMkhanyakude, a socioeconomically deprived rural district with an extremely high burden of HIV: 40% antenatal HIV prevalence and an annual HIV incidence of 5% in girls (15-19 years) and 8% in young women (20-24 years) (2, 16) and in three high-prevalence urban districts (HIV prevalence of greater than 10%) of City of Johannesburg, Ekurhuleni, and eThekwini.

Methods

Study design

We employed a longitudinal cohort (n~2000) and cross-sectional surveys (n~5000) of representative samples of AGYW aged 12-24 in four districts of South Africa with a high burden of HIV to measure the reach of MTV Shuga-DS. Data were collected between May 2017 and September 2019.

We use baseline and follow-up data from a nested cohort of ~2000 AGYW aged 13-22 years, enrolled in 2017 for the DREAMS impact evaluation. The cohort is nested in a large population-based longitudinal HIV surveillance study, in the uMkhanyakude district of KwaZulu-Natal (17, 18). A random sample of 3013 AGYW was selected from the surveillance population, stratified by age (13-17 years and 18-22 years) and geography, and invited to enrol in the nested cohort. Baseline interviews were conducted between May 2017 and February 2018 and follow-up interviews April 2018 and September 2019 in the local language (isiZulu) using a structured quantitative questionnaire programmed in REDCap (10).

The cross-sectional survey was conducted on a household-based representative sample of ~4000 AGYW (between the ages 12-24 years) in three high prevalence (City of Johannesburg, Ekurhuleni and eThekwini) districts. Between August 2017 and July 2018, a stratified cluster-

For peer review only - http://bmjopen.bmj.com/site/about/guidelines.xhtml

based sampling was used to select 18500 AGYW aged 12-24 eligible for a cross-sectional survey of individuals, based on an expected response rate of 80%.

The interview included questions on socio-demographics, general health, exposure to DREAMS and to MTV Shuga, sexual relationships, awareness and uptake of DREAMS and DREAMS-like services, migration, and gender norms across the four districts. A Dried Blood Spot (DBS) was taken at baseline and follow-up for Herpes Simplex Virus type 2 (HSV-2) antibody testing in the uMkhanyakude district. For sexual behaviour questions, participants were given a tablet computer to complete a self-interview; the fieldworker was available to provide support as needed.

Study setting and population

The cohort was nested within the Africa Health Research Institute (AHRI) triannual demographic surveillance of a population of approximately 150,000 people who are members of 15,000 geocoded households in an area of 845 km^2 (17). The study area is mostly rural and poor with high levels of youth unemployment (over 85% of those aged 18-24 are unemployed) (2, 16). From 2017, AHRI has embedded data collection clerks within the government clinics in the surveillance area to capture electronically any clinical attendance and the reason for the visit. Consenting individuals are linked to their surveillance identification number at the time of the clinic visit. This allows us to link exposure to an intervention with engagement with primary health care, including attendance for HIV care or use of contraceptive services.

The cross-sectional survey was conducted in three districts which were mostly urban with more towns and townships compared to AHRI surveillance area. The three study districts (City of Johannesburg, Ekurhuleni and eThekwini) consist of an estimated 12,073,421 individuals. The eThekwini district in KZN province is among those with the highest HIV prevalence (16.8% HIV prevalence in 2016) in South Africa. Over two-thirds (68%) of eThekwini is considered rural and 32% urban. About 11 963 (3%) AGYW in eThekwini are estimated to be living with HIV. The Gauteng province (GP), whilst geographically the smallest, is the most populous province in South Africa. GP has the fifth highest provincial HIV prevalence in the country with a prevalence of 11.1% among those aged 15 to 49 years old in 2016. The HIV prevalence in the two districts, City of Johannesburg and Ekurhuleni, is 11.1% and 14.3% among 15- to 49-year-olds, respectively. Both districts are densely populated and have high levels of industrialisation. The HIV prevalence amongst AGYW (15 to 24 years old) in the City of Johannesburg is 3%, and similarly 3% in Ekurhuleni in 2012.

For peer review only - http://bmjopen.bmj.com/site/about/guidelines.xhtml

Variables and Measurement:

Outcomes definitions:

We measured the effect of exposure to MTV Shuga on awareness and uptake of HIV prevention and SRHR outcomes at the follow-up visit. Our outcomes were: 1) self-reported HIV-testing in the past 12 months; 2) awareness of Pre-Exposure Prophylaxis (PrEP) for HIV prevention; 3) condom use at last sex; 4) use of contraception; 5) any new pregnancy since baseline; and 6) any new teenage pregnancy (restricted to those under the age of 20). AGYW were considered to use contraception if they self-reported using modern/hormonal contraception (modern contraception was defined as current use of any modern contraceptives, i.e., excluding traditional methods), or self-reported consistent condom use (using condoms as a contraceptive method and at last sex). Condomless sex was calculated using 2019 data among participants who reported having had sex with the most recent partner in the past 12 months. Recent pregnancy was calculated as any new pregnancy that occurred between baseline and 2019, while teenage pregnancy was calculated as any new pregnancy that occurred between baseline and 2019 among participants aged below 20 years. We also examined the effect of exposure to MTV Shuga on incident HSV-2 infections, among those who were HSV2 negative at baseline.

Exposure definitions:

Exposure to MTV Shuga was defined as ever watched MTV Shuga between 2017 and 2018. The level of exposure was measured based on the content of the series MTV Shuga, defined using 15 questions used to assess knowledge of content of MTV Shuga series. A composite score was developed summing-up the correct responses. The scores ranged between 2 and 14, and the median being 4. The median was used as a cut-off to define level of exposure among those who watched the series. Consequently, the level of exposure was categorized into 3 levels: High (watching a MTV Shuga and being able to correctly respond to 5 or more questions on content); Medium (watching programme and being able to correctly respond to less than 5 questions); and None (not watched any MTV Shuga). This was further categorised into 3 levels: High (watching MTV Shuga and being able to recall the content from MTV Shuga); Medium (watching programme, but unable to recall content); and None (not being aware of and not watched any MTV Shuga).

Potential confounding variables:

We included socio-demographic and sexual behaviour characteristics of AGYW that were measured at baseline and exposure to HIV prevention. The socio-demographic variables included age (as measured at follow-up), household socio-economic status, education broken

For peer review only - http://bmjopen.bmj.com/site/about/guidelines.xhtml

down by those who are still in school and those who have completed school, geographic area (rural or peri-urban/urban), and migration in the last 12 months. The socio-economic status (SES) variable was constructed using Principal Component Analysis (PCA) based on ownership of household assets and characteristics such as access to piped water, type of toilet, electricity and type of cooking fuel (19). Further, potential individual-level confounders measured included exposure to DREAMS (defined either as ever been invited to participate in any of the DREAMS activities or ever used any of the DREAMS HIV prevention interventions in the past 12 months or since 2016 and phone ownership at baseline.

Laboratory:

The HerpeSelect® 2 ELISA IgG assay (FOCUS Diagnostics, Cypress, California, USA) for the qualitative detection of human IgG class antibodies to HSV-2 was used on Dried blood Spot (DBS) samples collected on Whatman 903 filter cards. The HerpeSelect ® 2 ELISA IgG assay uses purified type-specific gG-2 antigen immobilized on polystyrene microwells reducing the cross-reactivity issues as seen with viral lysate assays(20). The assay is validated for use with serum samples but was optimised for use with DBS in the AHRI Diagnostic Research Laboratory following comparative testing with plasma samples. During the initial evaluation of the HerpeSelect ® 2 ELISA IgG a select number of plasma samples were also tested by an external accredited pathology laboratory. A 6mm diameter punch of a DBS spot was incubated overnight in 150ul Assay Diluent for no more than 16 hours at 4°C. The assay was performed with 50ul of the eluent in accordance with the manufacturer's instructions. Following a disproportionately high number of positive results based on other studies and our experience we multiplied the mean cut-off calibrator absorbance values by a factor of 1.5 before determining the index value for each sample (21, 22). The HerpeSelect ® 2 ELISA IgG results are reported as positive (index value >1.10), equivocal (index value of ≥ 0.90 but ≤1.10) or negative (index value <0.90). All initial equivocal results were re-tested and those that re-tested equivocal are reported as equivocal. An incident HSV-2 was defined as having been negative at baseline and positive at follow-up. Those who were equivocal at either baseline or follow-up were not considered as a sero-conversions.

Statistical analyses:

We included only participants who had data available at baseline and follow-up for nested cohorts. We used Chi-square tests to compare baseline characteristics between AGYW who did and did not have any exposure to MTV Shuga. We used logistic regression to examine the effect of MTV Shuga on health outcomes, adjusting for exposure to DREAMS and all other potential confounders. Potential effect-modification of MTV Shuga by exposure to DREAMS

For peer review only - http://bmjopen.bmj.com/site/about/guidelines.xhtml

was examined by fitting an interaction term to fully adjusted model: likelihood ratio tests were used to compare models with and without interaction terms.

We calculated the proportion of AGYW who reported an outcome (condomless-sex, recent pregnant) or tested positive for HSV-2 at 12-month or 24-month follow-up; and estimated associations between MTV Shuga and each outcome using a logistic regression, adjusting for potential confounders (age, household and individual socio-demographic characteristics and sexual behaviour). For HSV-2 incidence, we included participants who tested negative at baseline and had at least 1 follow-up test result. For DREAMS exposure, we included data collected at baseline and 12-month follow-up. For health outcomes (consistent condom use, modern contraception, HIV testing, PrEP awareness), we used data collected at 24-month follow-up; and for HSV-2 and pregnancy incidence we used data collected at 12 and 24-month follow-up.

Propensity score logistic regression adjustment was used to estimate the causal effect of MTV Shuga on health outcomes. A propensity or probability of being exposed to MTV Shuga was measured by fitting a logistic regression with MTV Shuga exposure as an outcome and potential confounders. A logistic regression models adjusting for propensity scores were then used to predict the probability of an outcome for all participants and separately by age group, under two scenario (1) exposed to MTV Shuga and (2) Not exposed to MTV Shuga. The predicted probabilities were then used to calculate the marginal risk difference, prevalence ratio and odds ratio. Confidence intervals were generated by using a bootstrap procedure, repeating the estimation procedure described above in 1000 samples that were drawn with replacement from the complete dataset and calculating 95% confidence intervals from the resulting bootstrap distribution using the 2.5% and 97.5% percentiles.
All analyses were performed using Stata version 15 (StataCorp LP, College Station, Texas USA).

Reporting

The STROBE reporting guidelines were used to guide synthesis and standardise reporting of our results(23)

Ethics

The DREAMS Partnership impact evaluation protocol was reviewed and approved by the University of KwaZulu-Natal (UKZN) Biomedical Research Ethics Committee (BREC) (BFC 339/16), the London School of Hygiene & Tropical Medicine Research Ethics Committee (Ref 11835) and the AHRI Somkhele Community Advisory Board, the Associate Director of Science of the Center for Global Health (CGH) at the United States Centers for Disease Control and

For peer review only - http://bmjopen.bmj.com/site/about/guidelines.xhtml

For peer review only

Prevention (CDC) in Atlanta and the Department of Health, Province of KZN and Gauteng. Approval for the demographic surveillance and data collection in the clinics was granted by UKZN BREC. All participants provided separate informed consent for the questionnaires and the HSV2 sero-survey. Consent for follow-up interviews was provided separately. For participants aged <18 years, written parental consent and participant assent were provided. Individuals attending the clinics in the surveillance area provided informed consent to record the clinic visit and link to their surveillance identification number.

Patient and public involvement

The study did not involve patients. AHRI has a Public Engagement Unit which conducts community engagement activities with the local communities as part of study finding dissemination. The Community Advisory Board provided feedback of the study including design before approval from ethics review board. Study findings are being made publicly available to funders, participants and the public through webinars, study reports and open access journal articles.

Results:

Participants

Of 3013 potentially eligible AGYW randomly selected from the surveillance data set, 85.5% of those eligible consented to participate at baseline (Figure 2). Of the 2184 eligible participants that were surveyed at baseline, 2016 (92.3%) had at least one follow-up visit and contributed data to this analysis. From the cross-sectional survey, 4127 (22.6%) eligible participants were surveyed.

Figure 2 Flowchart showing AGYW follow-up from nested cohorts 2017-2019

For peer review only

Awareness and exposure to MTV Shuga

MTV Shuga exposure at baseline was low, with a total of 308 (14.1%) respondents reported watching at least one episode. In the cross-sectional analysis of the three districts, a total of 1477 (35.8%) reported watching any MTV Shuga. In the nested cohort 121 (5.5%) recalled any storyline. Similarly in the cross-sectional snapshot 276 (6.7%) recalled the storyline (high exposure).

Social demographic characteristics of adolescents and young people by exposure to MTV Shuga (Table 1&2)

Table 1 summarises the profiles of the nested cohort (n=2184) and cross-sectional surveys (n=4127) AGYW comparing those exposed to MTV Shuga with those not exposed. In summary those who had seen any MTV Shuga were more likely to be from households in the highest socioeconomic tertile (p<0.001) and more urbanised areas (p<0.001). They were also more likely to have also received DREAMS (p=0.015) (Table 1). In the cross-sectional surveys (n=4127), MTV Shuga exposure was associated with older age (p<0.001), tertiary education (p <0.001) and never having sex (p< 0.001) Table 1.

Table 1. Baseline socio-demographic characteristics of adolescent girls and young women (13-22) by exposure to MTV Shuga in the nested cohort (n=2184)

	Overall		Exposed		Not exposed		
	n/N	%	n/N	%	n/N	%	p-value
**Age group (4 cats), 2017**							
13-14	460/2184	21.1	72/308	23.4	388/1876	20.7	
15-17	688/2184	31.5	107/308	34.7	581/1876	31	
18-19	475/2184	21.7	60/308	19.5	415/1876	22.1	
20-22	561/2184	25.7	69/308	22.4	492/1876	26.2	0.216
**Currently in school**							
No	540/2184	24.7	67/308	21.8	473/1876	25.2	
Yes	1644/2184	75.3	241/308	78.2	1403/1876	74.8	0.192
**Socio-economic status, 2018**							
Low	255/2118	12	22/303	7.3	233/1815	12.8	
Middle	920/2118	43.4	110/303	36.3	810/1815	44.6	
High	943/2118	44.5	171/303	56.4	772/1815	42.5	<0.001
**Urban or rural**							
Rural	1388/2165	64.1	165/305	54.1	1223/1860	65.8	
Peri-urban/urban	777/2165	35.9	140/305	45.9	637/1860	34.2	<0.001
**Invited or received DREAMS, 2017/18**	1101/2184	50.4	175/308	56.8	926/1876	49.4	0.015

For peer review only

For peer review only - http://bmjopen.bmj.com/site/about/guidelines.xhtml

**Away from home in the last 12 months**	314/1853	17.0	45/228	15.6	269/1565	17.2	0.516

Baseline socio-demographic characteristics of adolescent girls and young women (12-24) by exposure to MTV Shuga in the cross-sectional survey (n=4127)

	**Overall**		**Exposed**		**Not exposed**		
	n/N	%	n/N	%	n/N	%	**p-value**
**Age group, (N=4127)**							
12-14	958/4127	23.2	307/1477	20.1	651/2650	24.6	
15-19	1628/4127	39.5	599/1477	40.6	1029/2650	38.8	
20-24	1541/4127	37.3	571/1477	38.7	970/2650	36.6	0.022
**District**							
City of Johannesburg	1146/4127	27.8	476/1477	32.2	670/2650	25.3	
Ekurhuleni	1635/4127	39.6	521/1477	35.3	1114/2650	42.0	
eThekwini	1342/4127	32.5	480/1477	32.5	862/2650	32.5	<0.001
**Highest Education (N=4108)**							
No schooling	175/4108	4.3	99/1465	6.8	76/2646	2.9	
Grade R to 7	502/4108	12.2	142/1465	9.7	360/2646	13.6	
Grade 8 to 12	2978/4108	72.5	1022/1465	69.8	1956/2646	74.0	
Tertiary studies (complete/incomplete)	453/4108	11.0	202/1465	13.8	251/2646	9.5	<0.001
**Ever had sex with a boy/man (n=4108)**	1860/4108	45.3	621/1469	42.3	1239/2639	47.0	0.004
**Away from home in the last 12 months (n=4121)**	183/4121	4.4	66/1474	4.5	117/2647	4.4	0.932

After adjustment for confounders in nested cohorts (Table 2), AGYW from wealthier households (aOR=2.04 95%CI 1.27-3.30), peri-urban or urban areas (aOR=1.54 95%CI 1.19-1.98) and those invited to DREMS (aOR=1.48 95%CI 1.14-1.92) were more likely to be exposed to MTV Shuga than those from poor households, from rural areas and those not invited to DREAMS respectively. Similarly, after adjustment in the cross-sectional surveys (Table 2), AGYW with higher education were more likely to be exposed to MTV Shuga (aOR=2.58 95%CI 1.81-3.69) than those with less and those who ever had sex (aOR=0.68 95%CI 0.57-0.82) were less likely to be exposed to MTV Shuga.

For peer review only - http://bmjopen.bmj.com/site/about/guidelines.xhtml

Table 2 Factors associated with exposure to MTV Shuga in the nested cohort of AGYW aged 13-22 (n=2184)

	Unadjusted		Adjusted - All		
	OR	95%CI	OR	95%CI	P-value
**Age group, 2017**					
13-14	1		1		
15-17	0.99	0.72 -1.37	0.93	0.66 -1.29	
18-19	0.78	0.54 -1.13	0.77	0.51 -1.14	
20-22	0.76	0.53 -1.08	0.78	0.50 -1.21	0.555
**Currently in school**					
No	1		1		
Yes	1.21	0.91 -1.62	1.06	0.78 -1.43	0.726
**Socio-economic status, 2018**					
Low	1		1		
Middle	1.44	0.89 -2.33	1.27	0.78 -2.07	
High	2.35	1.47 -3.74	2.04	1.27 -3.30	<0.001
**Site**					
Rural	1		1		
Peri-urban/ urban	1.63	1.28 -2.08	1.54	1.19 -1.98	0.001
**Invited or received DREAMS, 2017/18**					
No	1		1		
Yes	1.35	1.06 -1.72	1.48	1.14 -1.92	0.003

Factors associated with exposure to MTV Shuga in the cross-sectional analysis of AGYW aged 12-24 (n=4127)

	Unadjusted		Adjusted - all		
	**OR**	**95% CI**	**OR**	**95% CI**	**P-value**
**District**					
City of Johannesburg	1		1		
Ekurhuleni	0.69	0.54 - 0.89	0.66	0.52- 0.84	0.001
eThekwini	0.79	0.60 - 1.04	0.78	0.59- 1.04	0.087
**Age group**					
12-14	1		1		
15-19	1.27	1.06 - 1.53	1.18	0.95- 1.46	0.126
20-24	1.22	1.01 - 1.48	1.16	0.90- 1.49	0.256
**Highest education level**					
Grade R to 7	1		1		
No schooling	2.88	1.81 - 4.58	3.29	2.01 - 5.38	<0.001
Grade 8 to 12	1.38	1.09 - 1.75	1.42	1.08 - 1.86	0.011

For peer review only - http://bmjopen.bmj.com/site/about/guidelines.xhtml

For peer review only

Complete or incomplete tertiary	2.29	1.70 - 3.09	2.58	1.81 - 3.69	<0.001
**Ever had sex with a boy/man**					
No	1		1		
Yes	0.82	0.71 - 0.95	0.68	0.57- 0.82	<0.001
**Away from home in last 12 months**					
No	1		1		
Yes	1.15	0.80 - 1.64	1.21	0.83- 1.77	0.309

Relationship between MTV Shuga exposure and HIV and SRHR outcomes (Table 3&4)

In the nested cohorts by 2019, overall 63.3% of those aged 14-23 knew their HIV status, 13.4% were consistently using contraception, 20.0% were using condoms consistently, and about a third were aware of PrEP, 5% had a pregnancy and 15% acquired HSV2. There were higher proportions of contraception use, condom use and PrEP awareness among those exposed to MTV Shuga (Table 3). For survey sites (Table 3) overall, 85.0% knew their HIV status, over a fifth 22.6% were using contraception and about half using condoms 48.4%. About a tenth (7.5%) were aware of PrEP, with higher proportions of these being among those exposed to MTV Shuga.

Table 3: HIV and SRHR outcomes by exposure to MTV Shuga nested cohort 13-22 year olds n= 2167)

	Overall		Exposed (n=308)		Not exposed (n=1878)		P-value
	n/N	%	n/N	%	n/N	%	
Knowledge of HIV status, 2019	1083/1712	63.3	175/283	61.8	908/1429	63.5	0.587
Modern contraception, 2019	221/1651	13.4	56/271	20.7	165/1380	12	<0.001
Consistent condom use, 2019	168/838	20	41/141	29.1	127/697	18.2	0.003
Aware of PrEP, 2019	523/1712	30.5	124/283	43.8	399/1429	27.9	0.302
Pregnant in 2018/19	124/2184	5.7	20/308	6.5	104/1876	5.5	0.504
Teenage pregnancy	72/1395	5.16	55/1187	4.63	17/208	8.17	0.033
HSV-2 2018/19	241/1562	15.4	35/237	14.8	206/1325	15.5	0.760

HIV and SRHR outcomes by exposure to MTV Shuga weighted for sampling (cross sectional survey 12-24 year olds n=4127)

	Overall		Exposed		Not exposed		
	**n/N**	**%**	**n/N**	**%**	**n/N**	**%**	**p-value**

For peer review only

PrEP awareness (N=4127)	310/4127	7.5	148/1477	10.0	162/2650	6.1	<0.001
HIV test (self-report) (N=4127)	2529/2156	85.3	656/797	82.3	1500/1732	86.6	0.005
Condom use at last sex (N=1898)‡	918/1898	48.4	320/640	50.0	598/1258	47.5	0.310
Contraception use (N=4127)	934/4127	22.6	302/1477	20.5	632/2650	23.9	0.012

‡Restricted to those who ever had sex with man

At follow up, incident HSV-2 and teenage pregnancy were high, HSV-2 incidence was 15.26 and teenage pregnancy incidence was 9.86 per 100 person-years, respectively (Table 4).

Table 4: Rate of SRHR outcome by exposure to MTV Shuga (nested cohort 13-22 year olds n=2167)

**HSV-2 incidence rate**					
	person-time	n with HSV-2	Rate/ 100 py	[95% Conf.	Interval]
MTV Shuga Not exposed	1303.0	206	15.81	13.79	18.12
MTV Shuga Exposed	276.3	35	12.67	9.09	17.64
Total	1579.3	241	15.26	13.45	17.31
**Pregnancy incidence rates among all AGYW**					
	person-time	n pregnant	Rate/100 py	[95% Conf.	Interval]
MTV Shuga Not exposed	1068.7	104	9.73	8.03	11.79
MTV Shuga Exposed	188.5	20	10.61	6.84	16.44
Total	1257.2	124	9.86	8.27	11.76
**Pregnancy incidence rates among girls below 19 years**					
	person-time	n pregnant	Rate/100 py	[95% Conf.	Interval]
MTV Shuga Not exposed	668.7	55	8.22	6.31	10.71
MTV Shuga Exposed	123.9	17	13.72	8.53	22.07
Total	792.6	72	9.08	7.21	11.44

MTV Shuga and HIV prevention and SRHR awareness and uptake

In the nested cohort after adjusting for age, education, SES, area and DREAMS, MTV Shuga exposure in the AGYW cohort was associated with significantly greater awareness of PrEP (aOR=2.06, 95%CI: 1.57-2.70), contraception uptake (aOR=2.08, 95%CI: 1.45-2.98), and consistent condom use (aOR=1.84, 95%CI: 1.24-2.93). Watching MTV-Shuga was not associated with HIV testing (aOR=1.02,95%CI: 0.77-1.21) (Figure 3 and Supplementary Tables S1-S4). There was no effect modification by DREAMS exposure.

Figure 3 Forest plots showing the association between MTV Shuga exposure and HIV prevention and SRHR awareness and uptake in the nested cohorts.

For peer review only

Similarly, in the cross-sectional analysis, after adjusting for age, education, district, migration and sexual history, exposure to MTV-Shuga watching was associated with greater awareness of PrEP (aOR=1.7, 95%CI 1.20 - 2.43). However, there was no association with contraception, lower self-reported HIV testing, or condom use as shown in Figure 4 below (Supplementary Table 5).

Figure 4 Forest plots showing association between MTV Shuga exposure and HIV prevention and SRHR awareness and uptake in the cross-sectional surveys

Causal effect of MTV Shuga on health outcomes – HSV2

The causal analysis similarly finds no effect of MTV Shuga on HSV2, with a risk difference of 1.10 95% CI (-2.82- 5.38)%. Findings in the younger age group (aged 13-17) and the older age group (18-22) was similar (Supplementary Table 6).

Discussion

In our study of the population-level effect of a national broadcast of a TV-based edu-drama on HIV prevention and SRHR, exposure to MTV Shuga was associated with higher awareness of a novel HIV prevention intervention (PrEP). However, despite a very high incidence of HSV-2 and teenage pregnancy, MTV Shuga exposure was not significantly associated with safer sexual behaviour, uptake of contraception and HIV-testing or prevention of teenage pregnancy. Notably though, the size of the relationship and direction of effect between exposure to MTV Shuga, condom use and markers of unprotected sex (HSV-2 and pregnancy) was consistent with a possible relationship. These findings may be partly explained by our finding that less than one in 12 of the target age group had any exposure to MTV Shuga and only half of these had high exposure (defined as watching the MTV Shuga South African series and being able to recall the content), suggesting that one of the limiting factors for the effective use of TV-based edu-drama maybe the dose that young people are exposed to, particularly in rural and resource-constrained settings most affected by HIV.

Our inability to find a measurable population effect of mass-media edu-drama behaviour change campaign compared to the trial findings of the RCT is disappointing (15). However, firstly the direction and size of the relationships suggest that we may have been able to see an effect if the proportion exposed had been greater than 7%. Secondly, AGYW in urban settings were more likely to have been exposed to MTV Shuga and they are also more likely to be living in small towns and townships. Data from our settings suggest that young people in small towns and townships are more vulnerable to HIV (24) and sexual risk (25) and therefore it is possible that a real effect of MTV Shuga on this group was masked by their

For peer review only - http://bmjopen.bmj.com/site/about/guidelines.xhtml

greater risk for the outcome. Due to the low numbers with high levels of exposure to MTV Shuga we did not have the power to explore this hypothesis by looking at effect modification by geography or socioeconomic status.

The finding of a differential association of MTV Shuga exposure with awareness of PrEP, compared to uptake of HIV and contraception, suggests that whilst educational mass entertainment may be able to increase awareness and possibly demand for a service, it does not impact on accessibility of the service: i.e. it impacts the first two steps of the prevention cascade and not the final step(26). Well described barriers to uptake of HIV-testing and contraception in this area are internalized and externalized stigma, fear of judgement from health care workers and the social costs of accessing care in busy primary health care settings(27-29). Behaviour change intervention including mass communication campaigns can be constrained or facilitated by the context in which people live (14, 30). To optimise MTV Shuga's effect there may need to be parallel innovations in SRHR and HIV service delivery that makes the services easier to access. We aim to test this hypothesis by providing community-based delivery of HIV and SRHR services in the context of the MTV Shuga.

The behaviour change theory that underpins edu-drama as a vehicle for mass behaviour change communication (13) explicitly suggests that the audience, and especially the early adopters, need to be actively watching, rather than passively watching or listening (14, 15). TV watching in rural homesteads can be in the context of large, often grandparent-led households and competing chores and priorities. This coupled with the relatively late timing of the shows may explain why so few girls and young women were sufficiently engaged or immersed to be able to recall characters or story lines. Moreover, the timing of this analysis may have allowed insufficient time for early adopters to convey the message of the show.

Limitations

The limitations of our study are that a meaningful exposure to MTV Shuga was low and so whilst the size of the relationship and direction of effect between exposure to MTV Shuga, condom use, and markers of unprotected sex (HSV-2 and pregnancy) suggested a possible effect we did not have the power to show a significant relationship between exposure to MTV Shuga and SRHR outcomes. We also do not have the power to see a difference by dose and immersion. Furthermore, as this is an observational study, we cannot exclude the possibility that those who are exposed to MTV Shuga are systematically different in ways that impact on the outcome of interest, for example more exposed to social media and sexual health promotion and innovative technologies to support sexual health than those who were not.

For peer review only - http://bmjopen.bmj.com/site/about/guidelines.xhtml

Conclusions and implications for the future

- Meaningful exposure to MTV Shuga was low across rural and urban settings in South Africa and so additional efforts need to be made to reach young people and increase their immersion in promising edu-drama if it is to have the desired effect, especially in rural and deprived settings.
- MTV Shuga was an effective vehicle to raise awareness and promote newer HIV prevention technologies such as HIV PrEP.
- There was some suggestion that MTV Shuga improved uptake of some HIV prevention and sexual health technologies (contraception and condoms)
- There was less evidence from this observational study that it improved SRHR and HIV outcomes

We highlight the importance of evaluating the real-world scale up of promising interventions to understand both the reach and population effect as well as inform interventions to increase impact and equity.

Efforts to increase exposure, which have been rolled out as part of MTV Shuga in SA, such as social media, school-based or community-based MTV Shuga film clubs will need to be evaluated, both to understand whether or not they increase exposure and coverage and improve SRH and HIV outcomes. However, to have a significant impact on the HIV and SRH prevention and treatment cascades, demand generation in AGYW needs to be delivered in parallel with accessible service delivery models that support adherence and retention (26).

Contributorship

M.S., N.C., J.D., T.Z. N.K, G.H. and C.C. developed the study tools and performed the research. M.S., J.S. D.P., K.B., I.B., and S.F. designed the research study. T.S. and S.D. conducted the laboratory analysis. N.M. supported by K.B. conducted the statistical analysis and C.G. M.S wrote the first and final draft of the paper with input from N.C., N.M., G.H., J.S., I.B., G.C., K.B., C.C., T.S., and N.K. All authors have approved the final draft of the paper.

Competing interests

All authors declare they have no conflict of interest

For peer review only - http://bmjopen.bmj.com/site/about/guidelines.xhtml

Funding

The authors are grateful to the communities of the Hlabisa sub-district, Ekurhuleni district, Thekwini district and City of Johannesburg who contributed their data to this study and to all the staff at Africa Health Research Institute and EpiCentre who collected the data. We acknowledge Eskindir Shambullo for his input in this manuscript.

The impact evaluation of DREAMS and MTV Shuga is funded by the Bill and Melinda Gates Foundation (OPP1136774 and OPP1171600, http://www.gatesfoundation.org). Foundation staff advised the study team, but did not substantively affect the study design, instruments, interpretation of data, or decision to publish. The research leading to these results has received funding from the People Programme (Marie Curie Actions) of the European Union's seventh Framework Programme FP7/2007-2013 under REA grant agreement n° 612216. MS National Institutes of Health 5R01MH114560-03 funding acknowledgement Africa Health Research Institute is supported by core funding from the Wellcome Trust [Core grant number (201433/Z/16/Z)]. GH is supported by a fellowship from the Royal Society and the Wellcome Trust [Grant number 210479/Z/18/Z]. MS work is also supported by a BMGF 3ie grant at AHRI. This research was funded in whole, or in part, by the Wellcome Trust [Grant number 210479/Z/18/Z]. For the purpose of open access, the author has applied a CC BY public copyright licence to any Author Accepted Manuscript version arising from this submission.

Data sharing statement

Data are available on reasonable request.

References

1. HSRC. The fifth South African National HIV prevalence, incidence, behaviour and communication survey [Report]. http://www.hsrc.ac.za/uploads/pageContent/9234/SABSSMV_Impact_Assessment_Summary_ZA_ADS_cleared_PDFA4.pdf: South African Health Services Research Council 2017 [updated 01/11/2018.

2. Chimbindi N, Mthiyane N, Birdthistle I, Floyd S, McGrath N, Pillay D, et al. Persistently high incidence of HIV and poor service uptake in adolescent girls and young women in rural KwaZulu-Natal, South Africa prior to DREAMS. PLoS One. 2018;13(10):e0203193.

3. Department of Health RoSA. National Campaign for Young Women and Adolescent Girls. . 2016.

For peer review only - http://bmjopen.bmj.com/site/about/guidelines.xhtml

4. Saul J, Bachman G, Allen S, Toiv NF, Cooney C, Beamon TA. The DREAMS core package of interventions: A comprehensive approach to preventing HIV among adolescent girls and young women. PLOS ONE. 2018;13(12):e0208167.
5. PEPFAR. Preventing HIV in Adolescent Girls and Young Women: Guidance for PEPFAR Country

Teams on the DREAMS Partnership 2015.
6. PEPFAR. South Africa DREAMS overview https://www.pepfar.gov/documents/organization/253958.pdf: PEPFAR; 2017 [
7. Chang L, Serwadda D, Quinn T, Wawer M, Gray R, Reynolds S. Combination implementation for HIV prevention: moving from clinical trial evidence to population-level effects The Lancet Infectious Diseases 2013;13(1):65-76.
8. Iwuji CC, Orne-Gliemann J, Larmarange J, Balestre E, Thiebaut R, Tanser F, et al. Universal test and treat and the HIV epidemic in rural South Africa: a phase 4, open-label, community cluster randomised trial. Lancet HIV. 2018;5(3):e116-e25.
9. Iwuji CC, Orne-Gliemann J, Tanser F, Boyer S, Lessells RJ, Lert F, et al. Evaluation of the impact of immediate versus WHO recommendations-guided antiretroviral therapy initiation on HIV incidence: the ANRS 12249 TasP (Treatment as Prevention) trial in Hlabisa sub-district, KwaZulu-Natal, South Africa: study protocol for a cluster randomised controlled trial. Trials. 2013;14:230.
10. Birdthistle I, Schaffnit SB, Kwaro D, Shahmanesh M, Ziraba A, Kabiru CW, et al. Evaluating the impact of the DREAMS partnership to reduce HIV incidence among adolescent girls and young women in four settings: a study protocol. BMC Public Health. 2018;18(1):912.
11. Shen F, Han J. Effectiveness of entertainment education in communicating health information: a systematic review. Asian Journal of Communication. 2014;24(6):605-16.
12. Noar SM, Palmgreen P, Chabot M, Dobransky N, Zimmerman RS. A 10-year systematic review of HIV/AIDS mass communication campaigns: have we made progress? Journal of Health Communication. 2009;14(1):15-42.
13. Barker K. Sex Soap and Social change- The Sabedo methadology https://www.populationmedia.org/product/sabido-theory/; http://www.comminit.com/content/sabido-methodology [
14. Baker V, Birdthistle I, Seeley J, Arnold G, Piot S, Hajiyiannis H. How can a TV drama about Love, Sex and Growing up Increase Knowledge and Shape the Sexual Health Behaviours of Young People? . AIDS Impact; Cape Town, South Africa2017.

For peer review only

For peer review only - http://bmjopen.bmj.com/site/about/guidelines.xhtml

15. MTV Shuga Soap Opera Turns Edutainment into a Tool to Fight HIV and Gender-Based Violence http://www.worldbank.org/en/news/feature/2017/02/23/mtv-shuga-soap-opera-turns-edutainment-into-a-tool-to-fight-hiv-and-gender-based-violence2017 [

16. National Department of Health. The National Antenatal Sentinel HIV prevalence survey, South Africa, http://www.health.gov.za/index.php/shortcodes/2015-03-29-10-42-47/2015-04-30-08-18-10/2015-04-30-08-21-56?download=2584:2015-national-antenatal-hiv-prevalence-survey-final-23oct17, 2013 [

17. Herbst K, Law M, Geldsetzer P, Tanser F, Harling G, Barnighausen T. Innovations in health and demographic surveillance systems to establish the causal impacts of HIV policies. Current Opinion on HIV AIDS. 2015;10(6):483-94.

18. Gareta D, Baisley K, Mngomezulu T, Smit T, Khoza T, Nxumalo S, et al. Cohort Profile Update: Africa Centre Demographic Information System (ACDIS) and population-based HIV survey. Int J Epidemiol. 2021;50(1):33-4.

19. Filmer D, Pritchett LH. Estimating Wealth Effects Without Expenditure Data—Or Tears: An Application To Educational Enrollments In States Of India*. Demography. 2001;38:115-32.

20. Arvin A, Prober C. Herpes Simplex Viruses. In: Murray P, Baron E, Pfaller M, Tenover F, Yolkenet R, editors. Manual of Clinical Microbiology 6th Ed Washington D.C.: ASM; 1995. p. 876-83.

21. Delany-Moretlwe S, Jentsch U, Weiss H, Moyes J, Ashley-Morrow R, Stevens W, et al. Comparison of Focus HerpeSelect ® and Kalon TM HSV-2 gG2 ELISA serological assays to detect herpes simplex virus type 2 (HSV-2) antibodies in a South African population. . Sexually Transmitted Infections,. 2009;86(1):46-50.

22. Ashley-Morrow R, Nolkamper J, Robinson N, Bishop N, Smith J. Performance of Focus ELISA test for herpes simplex virus type 1 (HSV-1) and HSV-2 antibodies among women in ten diverse geographical locations. . Clinical Microbiology and Infection 2004;10(6):530-6.

23. STROBE Statement. STROBE Checklists 2007 [Available from: http://www.strobe-statement.org/index.php?id=available-checklists.

24. Cuadros DF, Li J, Branscum AJ, Akullian A, Jia P, Mziray EN, et al. Mapping the spatial variability of HIV infection in Sub-Saharan Africa: Effective information for localized HIV prevention and control. Scientific Reports. 2017;7(1):9093.

25. Ngwenya N, Gumede D, Shahmanesh M, McGrath N, Grant A, Seeley J. Community perceptions of the socio-economic structural context influencing HIV and TB risk, prevention and treatment in a high prevalence area in the era of antiretroviral therapy. African Journal of AIDS Research. 2018;17(1):72-81.

For peer review only

26. Hargreaves JR, Delany-Moretlwe S, Hallett TB, Johnson S, Kapiga S, Bhattacharjee P, et al. The HIV prevention cascade: integrating theories of epidemiological, behavioural, and social science into programme design and monitoring. Lancet HIV. 2016;3(7):e318-22.
27. Hargreaves JR, Krishnaratne S, Mathema H, Lilleston PS, Sievwright K, Mandla N, et al. Individual and community-level risk factors for HIV stigma in 21 Zambian and South African communities: analysis of data from the HPTN071 (PopART) study. AIDS. 2018;32(6):783-93.
28. Chimbindi N, Zuma T, Nakasone S, Birdthistle I, Mthiyane N, Dreyer J, et al. Platforms, Processes and Perceptions of PrEP Delivery Through DREAMS in a Rural Setting in KwaZulu-Natal, South Africa. . Research for Prevention 2018 (HIVR4P 2018) Madrid, Spain 2018.
29. Adeagbo O, Herbst C, Dlamini N, Mhlongo T, Luthuli M, Xulu S, et al. “If she tests negative, it means I am also negative”: Men’s construction of HIV testing in KwaZulu-Natal, South Africa. . HIV Research for Prevention (HIVR4P) 23 October 2018.; Madrid, Spain **2018**
30. Michie S, van Stralen MM, West R. The behaviour change wheel: A new method for characterising and designing behaviour change interventions. Implementation Science. 2011;6(42).

For peer review only

For peer review only - http://bmjopen.bmj.com/site/about/guidelines.xhtml

Figure legends

Figure 1 showing conceptual framework for MTV Shuga impact on HIV prevention on AGYW in uMkhanyakude

Figure 2 Flowchart showing AGYW follow-up from nested cohorts 2017-2019

Figure 3 Forest plots showing the association between MTV Shuga exposure and HIV prevention and SRHR awareness and uptake in the nested cohorts.

Figure 4 Forest plots showing association between MTV Shuga exposure and HIV prevention and SRHR awareness and uptake in the cross-sectional surveys

For peer review only

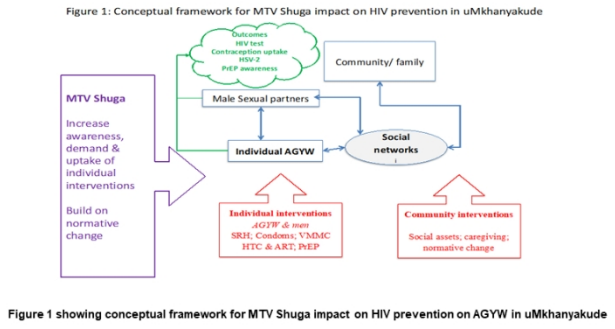

Figure 1 showing conceptual framework for MTV Shuga impact on HIV prevention on AGYW in uMkhanyakude

338x190mm (96 x 96 DPI)

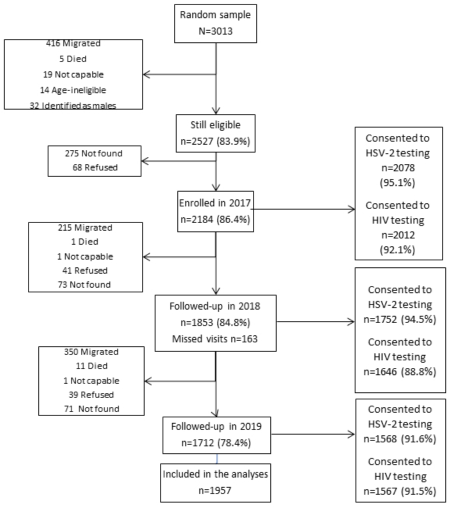

Figure 2 Flowchart showing AGYW follow-up from nested cohorts 2017-2019

338x190mm (96 x 96 DPI)

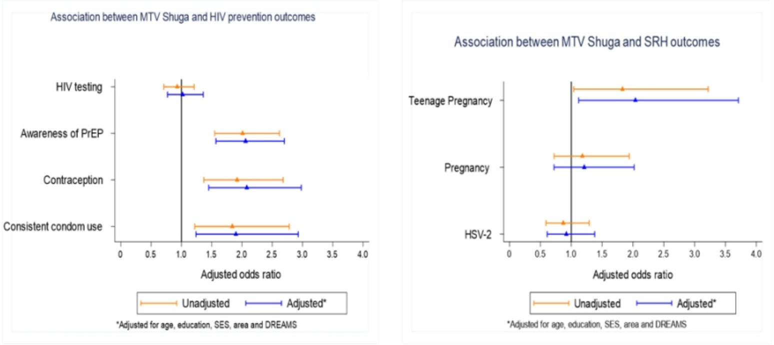

Figure 3 Forest plots showing the association between MTV Shuga exposure and HIV prevention and SRHR awareness and uptake in the nested cohorts.

338x190mm (96 x 96 DPI)

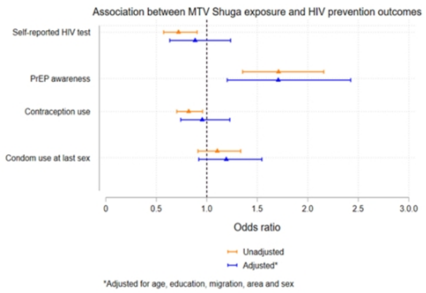

Figure 4 Forest plots showing association between MTV Shuga exposure and HIV prevention and SRHR awareness and uptake in the cross-sectional surveys

338x190mm (96 x 96 DPI)

Supplementary Table 1 Exposure to MTV Shuga and PrEP awareness in the nested cohort of AGYW aged 13-22 (n=2184)

	Unadjusted		Shuga adjusted			Adjusted-All		
	**OR**	**95%CI**	**OR**	**95%CI**	**p-value**	**OR**	**95%CI**	**p-value**
**Ever watched MTV Shuga, 2018/19**								
No	1					1		
Yes	2.01	1.55 -2.62				2.06	1.57 -2.70	<0.001
**Age**	1.12	1.08-1.16	1.12	1.08-1.17	<0.001	1.11	1.06-1.17	<0.001
**Currently in school**								
No	1		1			1		
Yes	0.6	0.47 -0.76	0.59	0.46 -0.75	<0.001	0.89	0.65 -1.23	0.488
**Socio-economic status, 2018**								
Low	1		1			1		
Middle	0.99	0.71 -1.39	0.96	0.68 -1.35		1.01	0.71 -1.44	
High	1.13	0.81 -1.58	1.05	0.75 -1.47	0.744	1.11	0.78 -1.58	0.699
**Urban or rural**								
Rural	1		1			1		
Peri-urban/urban	0.98	0.79 -1.22	0.93	0.75 -1.16	0.525	0.92	0.73 -1.15	0.459
**Invited or received DREAMS, 2017/18**								
No	1		1			1		
Yes	0.89	0.73 -1.10	0.88	0.71 -1.08	0.212	0.99	0.79 -1.23	0.897

For peer review only

Supplementary Table 2 Exposure to MTV Shuga and contraception uptake in the nested cohort of AGYW aged 13-22 (n=2184)

	Unadjusted		Shuga adjusted			Adjusted-All		
	**OR**	**95%CI**	**OR**	**95%CI**	**p-value**	**OR**	**95%CI**	**p-value**
**Ever watched MTV Shuga, 2018/19**								
No	1					1		
Yes	1.92	1.37 -2.68				2.08	1.45-2.98	<0.001
**Age**	1.26	1.19-1.33	1.27	1.20-1.34	<0.001	1.32	1.24-1.42	<0.001
**Currently in school**								
No	1		1			1		
Yes	0.6	0.44 -0.83	0.59	0.43-0.82	0.001	1.7	1.13-2.55	0.01
**Socio-economic status, 2018**								
Low	1		1			1		
Middle	0.79	0.51 -1.22	0.76	0.49-1.19		0.8	0.51-1.28	
High	0.86	0.55 -1.34	0.8	0.51-1.24	0.489	0.83	0.52-1.32	0.646
**Urban or rural**								
Rural	1		1			1		
Peri-urban/urban	0.91	0.68 -1.24	0.86	0.64-1.17	0.345	0.89	0.64-1.24	0.49
**Invited or received DREAMS, 2017/18**								
No	1		1			1		
Yes	0.84	0.63 -1.11	0.83	0.62-1.10	0.191	1.04	0.76-1.42	0.813

For peer review only

Supplementary Table 3 Exposure to MTV Shuga and consistent condom use in the nested cohort of AGYW aged 13-22 (n=2184)

	Unadjusted		Shuga adjusted			Adjusted-All		
	**OR**	**95%CI**	**OR**	**95%CI**	**p-value**	**OR**	**95%CI**	**p-value**
**Ever watched MTV Shuga, 2018/19**								
No	1					1		
Yes	1.84	1.22-2.78				1.9	1.24-2.93	0.003
**Age**	0.94	0.86-1.01	0.94	0.87-1.02	0.157	1	0.91-1.10	0.984
**Currently in school**								
No	1		1			1		
Yes	1.76	1.22-2.53	1.73	1.20-2.49	0.003	1.78	1.15-2.76	0.01
**Socio-economic status, 2018**								
Low	1		1			1		
Middle	1.01	0.60-1.70	0.97	0.58-1.63		0.98	0.58-1.67	
High	0.87	0.52-1.47	0.8	0.47-1.36	0.552	0.86	0.50-1.48	0.753
**Urban or rural**								
Rural	1		1			1		
Peri-urban/urban	0.7	0.48-1.02	0.67	0.46-0.97	0.035	0.71	0.48-1.05	0.088
**Invited or received DREAMS, 2017/18**								
No	1		1			1		
Yes	1.13	0.81-1.59	1.13	0.80-1.59	0.486	0.97	0.67-1.40	0.875

For peer review only

Supplementary Table 4 Exposure to MTV Shuga and HIV testing in the nested cohort of AGYW aged 13-22 (n=2184)

	**Unadjusted**		**Shuga adjusted**			**Adjusted-All**		
	**OR**	**95%CI**	**OR**	**95%CI**	**p-value**	**OR**	**95%CI**	**p-value**
**Ever watched MTV Shuga, 2018/19**								
No	1					1		
Yes	0.93	0.71-1.21				1.02	0.77-1.36	0.885
**Age**	1.3	1.25-1.35	1.3	1.24-1.35	<0.001	1.3	1.24-1.37	<0.001
**Currently in school**								
No	1		1			1		
Yes	0.37	0.28-0.48	0.37	0.28-0.48	0	1.07	0.74-1.53	0.726
**Socio-economic status, 2018**								
Low	1		1			1		
Middle	0.67	0.48-0.94	0.67	0.48-0.95		0.71	0.49-1.02	
High	0.58	0.41-0.81	0.58	0.41-0.81	0.007	0.6	0.41-0.86	0.018
**Urban or rural**								
Rural	1		1			1		
Peri-urban/urban	0.84	0.68-1.03	0.84	0.69-1.04	0.104	0.89	0.71-1.11	0.298
**Invited or received DREAMS, 2017/18**								
No	1		1			1		
Yes	0.84	0.69-1.02	0.84	0.69-1.02	0.081	0.98	0.79-1.23	0.89

For peer review only

Supplementary Table 5: Exposure to MTV Shuga with HIV prevention and SRHR awareness and uptake in the cross-sectional analysis of AGYW aged 12-24 (n=4127)

	aOR (95% Confidence Interval)	P-value
**District**		
City of Johannesburg	1	
Ekurhuleni	0.66 (0.47-0.91)	0.013
eThekwini	0.78 (0.54-1.13)	0.186
**Age group**		
12-14	1	
15-19	1.75 (0.35-8.84)	0.499
20-24	1.60 (0.32-8.07)	0.570
**Highest education level**		
Grade R to 7	1	
No schooling	3.84 (1.05-14.02)	0.041
Grade 8 to 12	3.17 (0.93-10.77)	0.065
Complete or incomplete tertiary	6.52 (1.90-22.33)	0.003
**Ever had sex with a boy/man**		
No	1	
Yes	0.67 (0.32-1.40)	0.286
**Away from home**		
No	1	
Yes	1.48 (0.93-2.37)	0.099
**PrEP awareness**		
No	1	
Yes	1.71 (1.20-2.43)	0.003
**Contraception use**		
No	1	
Yes	0.95 (0.74-1.23)	0.718
**Condom use at last sex**		
No	1	
Yes	1.19 (0.92-1.55)	0.183
**HIV test (self-report)**		
No	1	
Yes	0.88 (0.63-1.24)	0.471

For peer review only

Supplementary Table 6 Estimated causal effect of MTV Shuga on HSV-2 incidence, overall and by age group

Estimated causal effect of MTV Shuga on HSV-2 incidence, overall and by age group											
	% Outcome in total study population	Estimated % Outcome if no Shuga	95% CI	Estimated % Outcome if all get Shuga	95% CI	Risk Difference (%; PS adjusted)	95% CI	Prevalence Ratio (%; PS adjusted)	95% CI	Odds Ratio (%; PS adjusted)	95% CI
PS adjustment: Primary results											
Overall	17.3	17.2	14.71 - 19.67	18.0	14.51 - 22.32	0.80	-3.24 - 5.71	1.05	0.82 - 1.36	1.06	0.79 - 1.46
13-17 Years	13.2	12.4	9.85 - 15.16	15.7	11.63 - 20.26	3.34	-1.60 - 8.25	1.27	0.89 - 1.79	1.32	0.87 - 1.98
18-22 Years	24.3	25.8	21.01 - 30.74	22.0	15.19 - 30.17	-3.76	-11.92 - 5.68	0.85	0.59 - 1.24	0.81	0.51 - 1.34
**Sensitivity analyses**											
Regression under counterfactual framework											
Overall	17.3	17.2	14.74 - 19.66	17.8	14.50 - 22.08	0.65	-3.42 - 5.72	1.04	0.82 - 1.36	1.05	0.78 - 1.46
13-17 Years	13.2	12.42	9.86 - 15.21	15.9	11.85 - 20.49	3.47	-1.54 - 8.63	1.28	0.89 - 1.80	1.33	0.88 - 1.99
18-22 Years	24.3	25.7	20.78 - 30.63	21.3	14.88 - 28.28	-4.42	-12.28 - 4.95	0.83	0.57 - 1.22	0.78	0.48 - 1.31
PS stratification											
Overall	17.3	17.1	14.68 - 19.68	17.9	14.53 - 22.13	0.80	-3.21 - 5.52	1.09	0.84 - 1.40	1.11	0.82 - 1.51
PS weighting											

For peer review only

Overall	17.3	17.08	14.74 - 19.48	17.22	13.99 - 21.10	0.13	-3.76 - 4.63	1.01	0.79 - 1.30	1.01	0.76 - 1.37

For peer review only

STROBE Statement—checklist of items that should be included in reports of observational studies

	Item No	Recommendation	Page No
**Title and abstract**	1	(*a*) Indicate the study's design with a commonly used term in the title or the abstract	1
		(*b*) Provide in the abstract an informative and balanced summary of what was done and what was found	2
**Introduction**			
Background/rationale	2	Explain the scientific background and rationale for the investigation being reported	4
Objectives	3	State specific objectives, including any prespecified hypotheses	6
**Methods**			
Study design	4	Present key elements of study design early in the paper	6-7
Setting	5	Describe the setting, locations, and relevant dates, including periods of recruitment, exposure, follow-up, and data collection	7
Participants	6	(*a*) *Cohort study*—Give the eligibility criteria, and the sources and methods of selection of participants. Describe methods of follow-up<br>*Case-control study*—Give the eligibility criteria, and the sources and methods of case ascertainment and control selection. Give the rationale for the choice of cases and controls<br>*Cross-sectional study*—Give the eligibility criteria, and the sources and methods of selection of participants	7
		(*b*) *Cohort study*—For matched studies, give matching criteria and number of exposed and unexposed<br>*Case-control study*—For matched studies, give matching criteria and the number of controls per case	
Variables	7	Clearly define all outcomes, exposures, predictors, potential confounders, and effect modifiers. Give diagnostic criteria, if applicable	8-9
Data sources/ measurement	8*	For each variable of interest, give sources of data and details of methods of assessment (measurement). Describe comparability of assessment methods if there is more than one group	8-10
Bias	9	Describe any efforts to address potential sources of bias	9-10
Study size	10	Explain how the study size was arrived at	6-7
Quantitative variables	11	Explain how quantitative variables were handled in the analyses. If applicable, describe which groupings were chosen and why	8
Statistical methods	12	(*a*) Describe all statistical methods, including those used to control for confounding	9-10
		(*b*) Describe any methods used to examine subgroups and interactions	10
		(*c*) Explain how missing data were addressed	10
		(*d*) *Cohort study*—If applicable, explain how loss to follow-up was addressed<br>*Case-control study*—If applicable, explain how matching of cases and controls was addressed<br>*Cross-sectional study*—If applicable, describe analytical methods taking account of sampling strategy	10
		(*e*) Describe any sensitivity analyses	10

Continued on next page

For peer review only - http://bmjopen.bmj.com/site/about/guidelines.xhtml

**Results**			
Participants	13*	(a) Report numbers of individuals at each stage of study—eg numbers potentially eligible, examined for eligibility, confirmed eligible, included in the study, completing follow-up, and analysed	11
		(b) Give reasons for non-participation at each stage	11
		(c) Consider use of a flow diagram	11
Descriptive data	14*	(a) Give characteristics of study participants (eg demographic, clinical, social) and information on exposures and potential confounders	11
		(b) Indicate number of participants with missing data for each variable of interest	
		(c) *Cohort study*—Summarise follow-up time (eg, average and total amount)	
Outcome data	15*	*Cohort study*—Report numbers of outcome events or summary measures over time	12
		Case-control study—Report numbers in each exposure category, or summary measures of exposure	
		Cross-sectional study—Report numbers of outcome events or summary measures	12
Main results	16	(*a*) Give unadjusted estimates and, if applicable, confounder-adjusted estimates and their precision (eg, 95% confidence interval). Make clear which confounders were adjusted for and why they were included	12-16
		(*b*) Report category boundaries when continuous variables were categorized	12-16
		(*c*) If relevant, consider translating estimates of relative risk into absolute risk for a meaningful time period	
Other analyses	17	Report other analyses done—eg analyses of subgroups and interactions, and sensitivity analyses	17
**Discussion**			
Key results	18	Summarise key results with reference to study objectives	17-18
Limitations	19	Discuss limitations of the study, taking into account sources of potential bias or imprecision. Discuss both direction and magnitude of any potential bias	18
Interpretation	20	Give a cautious overall interpretation of results considering objectives, limitations, multiplicity of analyses, results from similar studies, and other relevant evidence	18-19
Generalisability	21	Discuss the generalisability (external validity) of the study results	17-19
**Other information**			
Funding	22	Give the source of funding and the role of the funders for the present study and, if applicable, for the original study on which the present article is based	20

*Give information separately for cases and controls in case-control studies and, if applicable, for exposed and unexposed groups in cohort and cross-sectional studies.

Note: An Explanation and Elaboration article discusses each checklist item and gives methodological background and published examples of transparent reporting. The STROBE checklist is best used in conjunction with this article (freely available on the Web sites of PLoS Medicine at http://www.plosmedicine.org/, Annals of Internal Medicine at http://www.annals.org/, and Epidemiology at http://www.epidem.com/). Information on the STROBE Initiative is available at www.strobe-statement.org.

For peer review only

BMJ Open

Evaluating use of mass-media communication intervention 'MTV-Shuga' on increased awareness and demand for HIV and sexual health services by adolescent girls and young women in South Africa: An observational study

Journal:	*BMJ Open*
Manuscript ID	bmjopen-2022-062804.R1
Article Type:	Original research
Date Submitted by the Author:	07-Sep-2022

Complete List of Authors:	Chimbindi, Natsayi; Africa Health Research Institute; University College London,<br>Mthiyane, Nondumiso; Africa Health Research Institute<br>Chidumwa, Glory ; Africa Health Research Institute<br>Zuma, Thembelihle; Africa Health Research Institute; University College London,<br>Dreyer, Jaco; Africa Health Research Institute<br>Birdthistle, Isolde; London School of Hygiene and Tropical Medicine Faculty of Epidemiology and Population Health, Department of Population Health<br>Floyd, Sian; London School of Hygiene & Tropical Medicine, Infectious Disease Epidemiology<br>Kyegombe, Nambusi; London School of Hygiene & Tropical Medicine<br>Grundy, Chris; London School of Hygiene and Tropical Medicine Faculty of Epidemiology and Population Health, Department of Population Health<br>Cawood, Cherie; Epicentre Health Research<br>Danaviah, Siva; Africa Health Research Institute<br>Smit, Theresa; Africa Health Research Institute<br>Pillay, Deenan; University College London,<br>Baisley, Kathy; London School of Hygiene and Tropical Medicine Faculty of Epidemiology and Population Health; Africa Health Research Institute<br>Harling, Guy; Africa Health Research Institute<br>Seeley, Janet; London School of Hygiene and Tropical Medicine, Department of Global Health &Development; Africa Health Research Institute<br>Shahmanesh, Maryam; University College London, Institute for Global Health; Africa Health Research Institute,

**Primary Subject Heading**:	Sexual health
Secondary Subject Heading:	HIV/AIDS, Public health, Sexual health
Keywords:	HIV & AIDS < INFECTIOUS DISEASES, PUBLIC HEALTH, PREVENTIVE MEDICINE

For peer review only - http://bmjopen.bmj.com/site/about/guidelines.xhtml

I, the Submitting Author has the right to grant and does grant on behalf of all authors of the Work (as defined in the below author licence), an exclusive licence and/or a non-exclusive licence for contributions from authors who are: i) UK Crown employees; ii) where BMJ has agreed a CC-BY licence shall apply, and/or iii) in accordance with the terms applicable for US Federal Government officers or employees acting as part of their official duties; on a worldwide, perpetual, irrevocable, royalty-free basis to BMJ Publishing Group Ltd ("BMJ") its licensees and where the relevant Journal is co-owned by BMJ to the co-owners of the Journal, to publish the Work in this journal and any other BMJ products and to exploit all rights, as set out in our licence.

The Submitting Author accepts and understands that any supply made under these terms is made by BMJ to the Submitting Author unless you are acting as an employee on behalf of your employer or a postgraduate student of an affiliated institution which is paying any applicable article publishing charge ("APC") for Open Access articles. Where the Submitting Author wishes to make the Work available on an Open Access basis (and intends to pay the relevant APC), the terms of reuse of such Open Access shall be governed by a Creative Commons licence – details of these licences and which Creative Commons licence will apply to this Work are set out in our licence referred to above.

Other than as permitted in any relevant BMJ Author's Self Archiving Policies, I confirm this Work has not been accepted for publication elsewhere, is not being considered for publication elsewhere and does not duplicate material already published. I confirm all authors consent to publication of this Work and authorise the granting of this licence.

For peer review only

Title page

Revised Title: **Evaluating use of mass-media communication intervention 'MTV-Shuga' on increased awareness and demand for HIV and sexual health services by adolescent girls and young women in South Africa: An observational study**

Authors: Natsayi Chimbindi*[1,2,3], Nondumiso Mthiyane*[2], Glory Chidumwa[1], Thembelihle Zuma[1,2,3], Jaco Dreyer[1], Isolde Birdthistle[4], Sian Floyd[4], Nambusi Kyegombe[4], Chris Grundy[4], Cherie Cawood[5], Siva Danaviah[1], Theresa Smit[1], Deenan Pillay[2], Kathy Baisley[1,3], Guy Harling[1,2,3,6,7], Janet Seeley[1,3,4], Maryam Shahmanesh§[1,2,3]

1. Africa Health Research Institute, KwaZulu-Natal, South Africa
2. University College London, Institute for Global Health, London, UK
3. University of KwaZulu-Natal, KwaZulu-Natal, South Africa
4. London School of Hygiene and Tropical Medicine, London, UK
5. Epicentre, South Africa
6. MRC/Wits Rural Public Health & Health Transitions Research Unit (Agincourt), University of the Witwatersrand, Gauteng, South Africa
7. Department of Epidemiology & Harvard Center for Population and Development Studies, Harvard T.H. Chan School of Public Health, Boston, USA

*Joint authors

§Corresponding author (full address details): Maryam Shahmanesh, Institute for Global Health, UCL, 3rd Floor, Mortimer Market Centre, Capper Street, London WC1E 6JB. m.shahmanesh@ucl.ac.uk. +44 7776 185 572

Key words (6): HIV, Adolescents, mass communication campaigns, Sexual Health, Teenage Pregnancy, South Africa.

Abstract (word count=300)

For peer review only - http://bmjopen.bmj.com/site/about/guidelines.xhtml

Objectives: Mass media edu-dramas have potential to increase demand for HIV and sexual and reproductive health(SRH) services among young people. MTVShuga:'MTV Shuga:Down South'(MTV Shuga-DS) is a serial mass media edu-drama designed to reduce HIV-related risk-behaviour and improve SRH outcomes in adolescents and young adults in South Africa. We investigate the effect of exposure to MTVShuga-DS during the scale-up of combination HIV-prevention interventions(DREAMS) on awareness and uptake of SRH and HIV-prevention services by adolescent girls and young women(AGYW).

Design: One longitudinal and three cross-sectional surveys of representative samples of AGYW

Setting: AGYW in four South African districts with high HIV-prevalence(>10%) (May 2017 and September 2019)

Participants: 6311AGYW aged 12-24

Measures: Using logistic-regression we measured the relationship between exposure to MTV Shuga-DS and awareness of Pre-Exposure Prophylaxis(PrEP), condom-use at last sex, uptake of HIV-testing or contraception, and incident pregnancy or HSV-2 infection.

Results: Within the rural cohort 2184(85.5%) of eligible sampled individuals were enrolled, of whom 92.6% had at least one follow-up visit; the urban cross-sectional surveys enrolled 4127(22.6%) of eligible sampled individuals. Self-report of watching at least one MTV Shuga-DS episode was 14.1%(cohort) and 35.8%(cross-section), while storyline recall was 5.5%(cohort) and 6.7%(cross-section).

In the cohort, after adjustment (for DREAMS-exposure, age, education, SES), MTVShuga-DS exposure was associated with increased PrEP awareness(aOR=2.06,95%CI:1.57-2.70), contraception uptake(aOR=2.08,95%CI:1.45-2.98) and consistent condom-use(aOR=1.84, 95%CI:1.24-2.93), but not with HIV-testing(aOR=1.02,95%CI:0.77-1.21) or acquiring HSV-2 (aOR=0.92, 95%CI:0.61-1.38). In the cross-sections, MTVShuga-DS was associated with greater PrEP awareness(aOR=1.7, 95%CI:1.20-2.43), but no other outcome.

Conclusions: Among both urban and rural AGYW in South Africa, MTVShuga-DS exposure was associated with increased PrEP awareness and improved demand for some HIV-prevention and SRH technologies but not sexual health outcomes. However, exposure to MTVShuga-DS was low. Given these positive indications, supportive programming may be required to raise exposure and allow future evaluation of edu-drama impact in this setting.

For peer review only - http://bmjopen.bmj.com/site/about/guidelines.xhtml

Strengths and limitations of this study

- Evaluated the real-world reach of nationally broadcast edu-drama focusing on adolescent sexual health in South Africa.
- Data collection focused on a vulnerable population of AGYW across four diverse high HIV-burden districts of South Africa that included both rural and urban settings.
- Study measured the reach of MTV-Shuga and the relationship between exposure to the edu-drama on awareness and demand for HIV prevention technologies in representative longitudinal cohort and cross-sectional samples of young women.
- Observational studies give no opportunity to infer the cause–effect relationship, in this case exposure to MTV-Shuga and impact on uptake of sexual health promotion and innovative technologies.

For peer review only

[word count 4531]

Introduction

HIV remains one of the gravest health problems facing young people living in sub-Saharan Africa (SSA). There are over 7.7 million people living with HIV in South Africa, with more than 200,000 new HIV infections annually in those aged 15-49 years (1). The highest incidence is in adolescent girls and young women (AGYW) (15-24 years) (1, 2). Although HIV incidence has been declining in South Africa, a 43% decline in the overall incidence rate between 2012 and 2017, from 4.0 to 2.3 seroconversion events per 100 person-years among 15-49 year old; it still remains high among AGYW in South Africa (3). In uMkhanyakude, HIV incidence was lower during roll-out of combination HIV prevention for AGYW (2016 to 2018) than in the previous 5-year period among 15- to 19-year-old females (4.5 new infections per 100 person-years as compared with 2.8; and lower among 20- to 24-year-olds (7.1/100 person-years as compared with 5.8) (4).

In response to the high HIV incidence in young people, the South African government launched the `She Conquers Campaign', and the US President's Emergency Fund for AIDS Relief and others are supporting the roll-out of Determined, Resilient, Empowered, AIDS free, Mentored, and Safe (DREAMS)(5-7). These programmes provide an evidence-based combination HIV prevention package, including HIV-testing and counselling for adolescent girls and young women (AGYW) and their male sexual partners, alongside universal test and treat and improved sexual health services (8, 9).

However, the key ingredient to the success of these multicomponent interventions will be the extent to which AGYW and their male partners at most risk of HIV will uptake and adhere to the active components of the intervention. This is challenging: uptake and retention of contraception and HIV treatment cascade by young people, even within population-wide Universal Test and Treat trials, has been suboptimal (2, 10, 11). Data from the baseline analysis for the DREAMS impact evaluation in uMkhanyakude district, rural KwaZulu-Natal (KZN) in 2015 suggest that less than 40% of girls (15-19 years) and boys and young men (15-29 years) had ever tested for HIV; linkage to HIV treatment was even lower (12). Contraception use prevalence was 20% in girls (15-19 years) and 50% in young women (20-24 years) and 21% of 15-19 year old girls had ever been pregnant (2).

It is against this backdrop that the fifth series of Shuga: 'MTV Shuga: Down South' (MTV Shuga-DS), a mass media serial edu-drama designed for SA, was broadcast on free-to-air South Africa National television. From March 8th 2017, MTV Shuga aired one episode per week for 12 weeks (with repeats). MTV Shuga is a mass media behaviour change campaign

For peer review only - http://bmjopen.bmj.com/site/about/guidelines.xhtml

that aims to improve sexual and reproductive health rights (SRHR). At the centre of the campaign, which includes radio and social media, is a TV-drama that weaves messages about HIV, family planning, transactional and intergenerational sex, safer and healthy sexual relationships, into storylines with young characters (http://www.mtvshuga.com/show/series-5/MTV Shuga-down-south/).

Mass media campaigns, have the potential to reach a large number of people and have been shown to improve knowledge and health behaviour of a range of health conditions, with more recent data suggesting that theoretically informed and targeted interventions are more likely to have an effect (13, 14). MTV Shuga-DS was designed to reduce HIV-related risk behaviour and improve SRHR outcomes in adolescents and young adults in SA. This was expected to be achieved through increasing young people's awareness of their sexual and reproductive health rights and demand for, and uptake of HIV and SRH prevention and treatment technologies. The show's characters explicitly model how to discuss issues that are sensitive or taboo. MTV Shuga use the technique of 'melodramas', where drama is created through the battles between stereotypical goodies and baddies, and the way in which the 'transitional' (often empathetic) character, begins as ambivalent but changes into a positive role model to promote positive behaviour change(15). This is a deliberate method to immerse the audience in the action, rather than passively watching or listening (16). AGYW, or at least early adopters, are anticipated to be immersed in the serial, able to classify and identify with the transitional characters and their outcomes. Pathways to behaviour change through MTV Shuga, therefore relate to the extent to which the observer, including early adopters, are immersed and critically engaged with the story. It also depends on a context which is supportive rather than disruptive (see the conceptual framework Figure 1 below) (17).

Figure 1 showing conceptual framework for MTV Shuga impact on HIV prevention on AGYW in uMkhanyakude

A cluster randomised controlled trial of community-viewings of MTV Shuga in Nigeria found that exposure to MTV Shuga significantly improved HIV knowledge and testing in both sexes, the intervention arm showing 35% more likely to test for HIV than the control arm. There was also a 60% reduction in genital chlamydia as a marker of recent sexual risk in women, amongst those exposed to MTV Shuga compared to those who were not (18). There were, however, fewer changes in social norms, particularly around gender-based violence. Further work suggested that the impact was greatest in those who were immersed in the narrative. The importance of immersion (classification of characters and identifying with them and observing outcomes) coupled with critical participation and an enabling context were also found in a

For peer review only - http://bmjopen.bmj.com/site/about/guidelines.xhtml

thematic analysis looking at how storylines in MTV Shuga-DS shaped awareness, knowledge and opinions of sexual health and personal relationships among young people in SA (17).

Whilst the Nigerian and SA studies provide evidence for the efficacy of MTV Shuga impacting on SRHR and HIV-testing behaviours in exposed individuals, there is little evidence of how this will translate into a population-level effect when nationally broadcast and in less controlled environments. In particular, it is not clear how the impact will spill-over to non-viewers and how innovations will diffuse when shown and watched by adolescents and young adults in a real-world scale-up. It is also unclear how such impact will differ according to: setting (rural or urban); differential digital literacy and access to social media (geographically and socioeconomically), and the dose and context of watching (shared viewing with family, friends or individual experience through social media).

We use the opportunity of MTV Shuga being broadcast in the context of an impact evaluation of DREAMS roll out (January 2016-September 2019) in a representative, population-based sample of young people (12) to describe the real-world reach of nationally broadcast MTV Shuga-DS. Further, we explore the hypothesis that exposure to a mass-media serial edu-drama, like MTV Shuga, will improve SRHR outcomes by increasing demand for, and uptake of, existing combination individual and community-based SRH and HIV prevention services for AGYW in four diverse settings including uMkhanyakude, a socioeconomically deprived rural district with an extremely high burden of HIV: 40% antenatal HIV prevalence and an annual HIV incidence of 5% in girls (15-19 years) and 8% in young women (20-24 years) (2, 19) and in three high-prevalence urban districts (HIV prevalence of greater than 10%) of City of Johannesburg, Ekurhuleni, and eThekwini.

Methods

Study design

We employed a longitudinal cohort and cross-sectional surveys of representative samples of AGYW aged 12-24 in four districts of South Africa with a high burden of HIV to measure the reach of MTV Shuga-DS. Data were collected between May 2017 and September 2019.

We used baseline and follow-up data from a nested cohort of 2184 AGYW aged 13-22 years, enrolled in 2017 for the DREAMS impact evaluation. The cohort is nested in a large population-based longitudinal HIV surveillance study, in the uMkhanyakude district of KwaZulu-Natal (20, 21). A random sample of 3013 AGYW was selected from the surveillance

For peer review only - http://bmjopen.bmj.com/site/about/guidelines.xhtml

population, stratified by age (13-17 years and 18-22 years) and geography, and invited to enrol in the nested cohort. Baseline interviews were conducted between May 2017 and February 2018 and follow-up interviews April 2018 and September 2019 in the local language (isiZulu) using a structured quantitative questionnaire programmed in REDCap (12).

The cross-sectional survey was conducted on a household-based representative sample of 4127 AGYW (between the ages 12-24 years) in three high prevalence (City of Johannesburg, Ekurhuleni and eThekwini) districts. Between August 2017 and July 2018, a stratified cluster-based sampling strategy was used to select 18500 AGYW aged 12-24 eligible for a cross-sectional survey of individuals, based on an expected response rate of 80% (22).

The interview included questions on socio-demographics, general health, exposure to DREAMS and to MTV Shuga, sexual relationships, awareness and uptake of DREAMS and DREAMS-like services, migration, and gender norms across the four districts. A Dried Blood Spot (DBS) was taken at baseline and follow-up for Herpes Simplex Virus type 2 (HSV-2) antibody testing in the uMkhanyakude district. For sexual behaviour questions, participants were given a tablet computer to complete a self-interview; the fieldworker was available to provide support as needed.

Study setting and population

The cohort was nested within the Africa Health Research Institute (AHRI) triannual demographic surveillance of a population of approximately 150,000 people who are members of 15,000 geocoded households in an area of 845 km^2 (20). The study area is mostly rural and poor with high levels of youth unemployment (over 85% of those aged 18-24 are unemployed) (2, 19).

The cross-sectional survey was conducted in three districts which were mostly urban with more towns and townships compared to AHRI surveillance area. The three study districts (City of Johannesburg, Ekurhuleni and eThekwini) consist of an estimated 12,073,421 individuals. The eThekwini district in KZN province is among those with the highest HIV prevalence (16.8% HIV prevalence in 2016) in South Africa. Over two-thirds (68%) of eThekwini is considered rural and 32% urban. About 11 963 (3%) AGYW in eThekwini are estimated to be living with HIV. The Gauteng province (GP), whilst geographically the smallest, is the most populous province in South Africa. GP has the fifth highest provincial HIV prevalence in the country with a prevalence of 11.1% among those aged 15 to 49 years old in 2016. The HIV prevalence in the two districts, City of Johannesburg and Ekurhuleni, is 11.1% and 14.3% among 15- to 49-

For peer review only

year-olds, respectively. Both districts are densely populated and have high levels of industrialisation. The HIV prevalence amongst AGYW (15 to 24 years old) in the City of Johannesburg is 3%, and similarly 3% in Ekurhuleni in 2012.

Variables and Measurement:

Outcomes definitions:

We measured the effect of exposure to MTV Shuga on awareness and uptake of HIV prevention and SRHR outcomes at the follow-up visit. Our outcomes were: 1) self-reported HIV-testing in the past 12 months; 2) awareness of Pre-Exposure Prophylaxis (PrEP) for HIV prevention; 3) condom use at last sex; 4) use of contraception; 5) any new pregnancy since baseline; and 6) any new teenage pregnancy (restricted to those under the age of 20). AGYW were considered to use contraception if they self-reported using pill, injection, Intrauterine Device (IUD), implant, sterilisation or self-reported consistent condom use (using condoms as a contraceptive method and at last sex). Condomless sex was calculated using 2019 data among participants who reported having had sex with the most recent partner in the past 12 months. Recent pregnancy was calculated as any new pregnancy that occurred between baseline and 2019, while teenage pregnancy was calculated as any new pregnancy that occurred between baseline and 2019 among participants aged below 20 years. We also examined the effect of exposure to MTV Shuga on incident HSV-2 infections, among those who were HSV2 negative at baseline.

Exposure definitions:

Exposure to MTV Shuga was defined as ever watched MTV Shuga between 2017 and 2018. The level of exposure was measured based on the content of the series MTV Shuga, defined using 15 questions used to assess knowledge of content of MTV Shuga series. A composite score was developed summing-up the correct responses. The scores ranged between 2 and 14, and the median being 4. The median was used as a cut-off to define level of exposure among those who watched the series. Consequently, the level of exposure was categorized into 3 levels: High (watching a MTV Shuga and being able to correctly respond to 5 or more questions on content); Medium (watching programme and being able to correctly respond to less than 5 questions); and None (not watched any MTV Shuga). This was further categorised into 3 levels: High (watching MTV Shuga and being able to recall the content from MTV Shuga); Medium (watching programme, but unable to recall content); and None (not being aware of and not watched any MTV Shuga).

For peer review only - http://bmjopen.bmj.com/site/about/guidelines.xhtml

Potential confounding variables:

We included socio-demographic and sexual behaviour characteristics of AGYW that were measured at baseline and exposure to HIV prevention. The socio-demographic variables included age (as measured at follow-up), household socio-economic status, education broken down by those who are still in school and those who have completed school, geographic area (rural or peri-urban/urban), and migration in the last 12 months. The socio-economic status (SES) variable was constructed using Principal Component Analysis (PCA) based on ownership of household assets and characteristics such as access to piped water, type of toilet, electricity and type of cooking fuel (23). Further, potential individual-level confounders measured included exposure to DREAMS (defined either as ever been invited to participate in any of the DREAMS activities or ever used any of the DREAMS HIV prevention interventions in the past 12 months or since 2016 and phone ownership at baseline.

Laboratory:

The HerpeSelect® 2 ELISA IgG assay (FOCUS Diagnostics, Cypress, California, USA) for the qualitative detection of human IgG class antibodies to HSV-2 was used on Dried blood Spot (DBS) samples collected on Whatman 903 filter cards. The HerpeSelect ® 2 ELISA IgG assay uses purified type-specific gG-2 antigen immobilized on polystyrene microwells reducing the cross-reactivity issues as seen with viral lysate assays(24). The assay is validated for use with serum samples but was optimised for use with DBS in the AHRI Diagnostic Research Laboratory following comparative testing with plasma samples. During the initial evaluation of the HerpeSelect ® 2 ELISA IgG a select number of plasma samples were also tested by an external accredited pathology laboratory. A 6mm diameter punch of a DBS spot was incubated overnight in 150ul Assay Diluent for no more than 16 hours at 4°C. The assay was performed with 50ul of the eluent in accordance with the manufacturer's instructions. Following a disproportionately high number of positive results based on other studies and our experience we multiplied the mean cut-off calibrator absorbance values by a factor of 1.5 before determining the index value for each sample (25, 26). The HerpeSelect ® 2 ELISA IgG results are reported as positive (index value >1.10), equivocal (index value of ≥ 0.90 but ≤1.10) or negative (index value <0.90). All initial equivocal results were re-tested and those that re-tested equivocal are reported as equivocal. An incident HSV-2 was defined as having been negative at baseline and positive at follow-up. Those who were equivocal at either baseline or follow-up were not considered as a sero-conversions.

For peer review only - http://bmjopen.bmj.com/site/about/guidelines.xhtml

Statistical analyses:

We conducted two separate analyses for cohort and cross-sectional data. For the nested cohort, we included only participants who had data available at baseline and follow-up. We used Chi-square tests to compare baseline characteristics between AGYW who did and did not have any exposure to MTV Shuga. We used logistic regression to examine the effect of MTV Shuga on health outcomes, adjusting for exposure to DREAMS and all other potential confounders. Potential effect-modification of MTV Shuga by exposure to DREAMS was examined by fitting an interaction term to fully adjusted model: likelihood ratio tests were used to compare models with and without interaction terms.

We calculated the proportion of AGYW who reported an outcome (condomless-sex, recent pregnant) or tested positive for HSV-2 at 12-month or 24-month follow-up; and estimated associations between MTV Shuga and each outcome using a logistic regression, adjusting for potential confounders (age, household and individual socio-demographic characteristics and sexual behaviour). For HSV-2 incidence, we included participants who tested negative at baseline and had at least 1 follow-up test result. For DREAMS exposure, we included data collected at baseline and 12-month follow-up. For health outcomes (consistent condom use, modern contraception, HIV testing, PrEP awareness), we used data collected at 24-month follow-up; and for HSV-2 and pregnancy incidence we used data collected at 12 and 24-month follow-up.

Propensity score logistic regression adjustment was used to estimate the causal effect of MTV Shuga on health outcomes. A propensity or probability of being exposed to MTV Shuga was measured by fitting a logistic regression with MTV Shuga exposure as an outcome and potential confounders. A logistic regression models adjusting for propensity scores were then used to predict the probability of an outcome for all participants and separately by age group, under two scenario (1) exposed to MTV Shuga and (2) Not exposed to MTV Shuga. The predicted probabilities were then used to calculate the marginal risk difference, prevalence ratio and odds ratio. Confidence intervals were generated by using a bootstrap procedure, repeating the estimation procedure described above in 1000 samples that were drawn with replacement from the complete dataset and calculating 95% confidence intervals from the resulting bootstrap distribution using the 2.5% and 97.5% percentiles. We also used propensity score stratification and probability weighting methods to check the consistency of our findings.

For peer review only - http://bmjopen.bmj.com/site/about/guidelines.xhtml

For cross-sectional survey, we used Chi square test to compare the characteristics of AGYW who did and did not have any exposure to MTV Shuga; and logistic regression models adjusting for potential confounders were used to examine the association between exposure to MTV Shuga and health outcomes. Sampling weights were applied to achieve proportionality between groups of participants in the survey.

All analyses were performed using Stata version 15 (StataCorp LP, College Station, Texas USA).

Reporting

The STROBE reporting guidelines were used to guide synthesis and standardise reporting of our results(27)

Patient and public involvement

The study did not involve patients. AHRI has a Public Engagement Unit which conducts community engagement activities with the local communities as part of study findings dissemination. The Community Advisory Board provided feedback of the study including design before approval from ethics review board. Study findings are being made publicly available to funders, participants and the public through webinars, study reports and open access journal articles.

Results:

Participants

Of 3013 potentially eligible AGYW randomly selected from the surveillance data set, 85.5% of those eligible consented to participate at baseline (Figure 2). Of the 2184 eligible participants that were surveyed at baseline, 2016 (92.3%) had at least one follow-up visit and contributed data to this analysis. From the cross-sectional survey, 4127 (22.6%) eligible participants were surveyed.

Figure 2 Flowchart showing AGYW follow-up from nested cohorts 2017-2019

Awareness and exposure to MTV Shuga

MTV Shuga exposure at baseline was low, with a total of 308 (14.1%) respondents reported watching at least one episode. In the cross-sectional analysis of the three districts, a total of 1477 (35.8%) reported watching any MTV Shuga. In the nested cohort 121 (5.5%) recalled

For peer review only - http://bmjopen.bmj.com/site/about/guidelines.xhtml

any storyline. Similarly in the cross-sectional snapshot 276 (6.7%) recalled the storyline (high exposure).

Social demographic characteristics of adolescents and young people by exposure to MTV Shuga (Table 1&2)

Table 1 summarises the profiles of the nested cohort (n=2184) and cross-sectional surveys (n=4127) AGYW comparing those exposed to MTV Shuga with those not exposed. In summary those who had seen any MTV Shuga were more likely to be from households in the highest socioeconomic tertile ($p<0.001$) and more urbanised areas ($p<0.001$). They were also more likely to have also received DREAMS ($p=0.015$) (Table 1). In the cross-sectional surveys (n=4127), MTV Shuga exposure was associated with older age ($p<0.001$), tertiary education ($p <0.001$) and never having sex ($p< 0.001$) Table 1.

Table 1. Baseline socio-demographic characteristics of adolescent girls and young women (13-22) by exposure to MTV Shuga in the nested cohort (n=2184)

	Overall		Exposed		Not exposed		
	n/N	%	n/N	%	n/N	%	p-value
**Age group (4 cats), 2017**							
13-14	460/2184	21.1	72/308	23.4	388/1876	20.7	
15-17	688/2184	31.5	107/308	34.7	581/1876	31	
18-19	475/2184	21.7	60/308	19.5	415/1876	22.1	
20-22	561/2184	25.7	69/308	22.4	492/1876	26.2	0.216
**Currently in school**							
No	540/2184	24.7	67/308	21.8	473/1876	25.2	
Yes	1644/2184	75.3	241/308	78.2	1403/1876	74.8	0.192
**Socio-economic status, 2018**							
Low	255/2118	12	22/303	7.3	233/1815	12.8	
Middle	920/2118	43.4	110/303	36.3	810/1815	44.6	
High	943/2118	44.5	171/303	56.4	772/1815	42.5	<0.001
**Urban or rural**							
Rural	1388/2165	64.1	165/305	54.1	1223/1860	65.8	
Peri-urban/urban	777/2165	35.9	140/305	45.9	637/1860	34.2	<0.001
**Invited or received DREAMS, 2017/18**	1101/2184	50.4	175/308	56.8	926/1876	49.4	0.015
**Away from home in the last 12 months**	314/1853	17.0	45/228	15.6	269/1565	17.2	0.516

Baseline socio-demographic characteristics of adolescent girls and young women (12-24) by exposure to MTV Shuga in the cross-sectional survey (n=4127)

	Overall		Exposed		Not exposed		

For peer review only - http://bmjopen.bmj.com/site/about/guidelines.xhtml

	n/N	%	n/N	%	n/N	%	p-value
**Age group, (N=4127)**							
12-14	958/4127	23.2	307/1477	20.1	651/2650	24.6	
15-19	1628/4127	39.5	599/1477	40.6	1029/2650	38.8	
20-24	1541/4127	37.3	571/1477	38.7	970/2650	36.6	0.022
**District**							
City of Johannesburg	1146/4127	27.8	476/1477	32.2	670/2650	25.3	
Ekurhuleni	1635/4127	39.6	521/1477	35.3	1114/2650	42.0	
eThekwini	1342/4127	32.5	480/1477	32.5	862/2650	32.5	<0.001
**Highest Education (N=4108)**							
No schooling	175/4108	4.3	99/1465	6.8	76/2646	2.9	
Grade R to 7	502/4108	12.2	142/1465	9.7	360/2646	13.6	
Grade 8 to 12	2978/4108	72.5	1022/1465	69.8	1956/2646	74.0	
Tertiary studies (complete/incomplete)	453/4108	11.0	202/1465	13.8	251/2646	9.5	<0.001
**Ever had sex with a boy/man (n=4108)**	1860/4108	45.3	621/1469	42.3	1239/2639	47.0	0.004
**Away from home in the last 12 months (n=4121)**	183/4121	4.4	66/1474	4.5	117/2647	4.4	0.932

After adjustment for confounders in nested cohorts (Table 2), AGYW from wealthier households (aOR=2.04 95%CI 1.27-3.30), peri-urban or urban areas (aOR=1.54 95%CI 1.19-1.98) and those invited to DREAMS (aOR=1.48 95%CI 1.14-1.92) were more likely to be exposed to MTV Shuga than those from poor households, from rural areas and those not invited to DREAMS respectively. Similarly, after adjustment in the cross-sectional surveys (Table 2), AGYW with higher education were more likely to be exposed to MTV Shuga (aOR=2.58 95%CI 1.81-3.69) than those with less and those who ever had sex (aOR=0.68 95%CI 0.57-0.82) were less likely to be exposed to MTV Shuga.

Table 2 Factors associated with exposure to MTV Shuga in the nested cohort of AGYW aged 13-22 (n=2184)

	Unadjusted		Adjusted - All		
	OR	95%CI	OR	95%CI	P-value
**Age group, 2017**					
13-14	1		1		
15-17	0.99	0.72 -1.37	0.93	0.66 -1.29	
18-19	0.78	0.54 -1.13	0.77	0.51 -1.14	
20-22	0.76	0.53 -1.08	0.78	0.50 -1.21	0.555
**Currently in school**					
No	1		1		
Yes	1.21	0.91 -1.62	1.06	0.78 -1.43	0.726

For peer review only - http://bmjopen.bmj.com/site/about/guidelines.xhtml

**Socio-economic status, 2018**					
Low	1		1		
Middle	1.44	0.89 -2.33	1.27	0.78 -2.07	
High	2.35	1.47 -3.74	2.04	1.27 -3.30	<0.001
**Site**					
Rural	1		1		
Peri-urban/ urban	1.63	1.28 -2.08	1.54	1.19 -1.98	0.001
**Invited or received DREAMS, 2017/18**					
No	1		1		
Yes	1.35	1.06 -1.72	1.48	1.14 -1.92	0.003

Factors associated with exposure to MTV Shuga in the cross-sectional analysis of AGYW aged 12-24 (n=4127)

	**Unadjusted**		**Adjusted - all**		
	**OR**	**95% CI**	**OR**	**95% CI**	**P-value**
**District**					
City of Johannesburg	1		1		
Ekurhuleni	0.69	0.54 - 0.89	0.66	0.52- 0.84	0.001
eThekwini	0.79	0.60 - 1.04	0.78	0.59- 1.04	0.087
**Age group**					
12-14	1		1		
15-19	1.27	1.06 - 1.53	1.18	0.95- 1.46	0.126
20-24	1.22	1.01 - 1.48	1.16	0.90- 1.49	0.256
**Highest education level**					
Grade R to 7	1		1		
No schooling	2.88	1.81 - 4.58	3.29	2.01 - 5.38	<0.001
Grade 8 to 12	1.38	1.09 - 1.75	1.42	1.08 - 1.86	0.011
Complete or incomplete tertiary	2.29	1.70 - 3.09	2.58	1.81 - 3.69	<0.001
**Ever had sex with a boy/man**					
No	1		1		
Yes	0.82	0.71 - 0.95	0.68	0.57- 0.82	<0.001
**Away from home in last 12 months**					
No	1		1		
Yes	1.15	0.80 - 1.64	1.21	0.83- 1.77	0.309

For peer review only - http://bmjopen.bmj.com/site/about/guidelines.xhtml

Relationship between MTV Shuga exposure and HIV and SRHR outcomes (Table 3&4)

In the nested cohorts by 2019, overall 63.3% of those aged 14-23 knew their HIV status, 13.4% were consistently using contraception, 20.0% were using condoms consistently, and about a third were aware of PrEP, 5% had a pregnancy and 15% acquired HSV2. There were higher proportions of contraception use, condom use and PrEP awareness among those exposed to MTV Shuga (Table 3). For survey sites (Table 3) overall, 85.0% knew their HIV status, over a fifth 22.6% were using contraception and about half using condoms 48.4%. About a tenth (7.5%) were aware of PrEP, with higher proportions of these being among those exposed to MTV Shuga.

Table 3: HIV and SRHR outcomes by exposure to MTV Shuga nested cohort 13-22 year olds (n= 2167)

	Overall		Exposed (n=308)		Not exposed (n=1878)		P-value
	n/N	%	n/N	%	n/N	%	
Knowledge of HIV status, 2019	1083/1712	63.3	175/283	61.8	908/1429	63.5	0.587
Modern contraception, 2019	221/1651	13.4	56/271	20.7	165/1380	12	<0.001
Consistent condom use, 2019	168/838	20	41/141	29.1	127/697	18.2	0.003
Aware of PrEP, 2019	523/1712	30.5	124/283	43.8	399/1429	27.9	0.302
Pregnant in 2018/19	124/2184	5.7	20/308	6.5	104/1876	5.5	0.504
Teenage pregnancy	72/1395	5.16	55/1187	4.63	17/208	8.17	0.033
HSV-2 2018/19	241/1562	15.4	35/237	14.8	206/1325	15.5	0.760
**HIV and SRHR outcomes by exposure to MTV Shuga weighted for sampling cross sectional survey 12-24 year olds (n=4127)**							
	**Overall**		**Exposed**		**Not exposed**		
	**n/N**	**%**	**n/N**	**%**	**n/N**	**%**	**p-value**
PrEP awareness (N=4127)	310/4127	7.5	148/1477	10.0	162/2650	6.1	<0.001
HIV test (self-report) (N=2529)	2156/2529	85.3	656/797	82.3	1500/1732	86.6	0.005
Condom use at last sex							0.310
(N=1898)‡	918/1898	48.4	320/640	50.0	598/1258	47.5	
Contraception use (N=4127)	934/4127	22.6	302/1477	20.5	632/2650	23.9	0.012

‡Restricted to those who ever had sex with man

At follow up, incident HSV-2 and teenage pregnancy were high, HSV-2 incidence was 15.26 and teenage pregnancy incidence was 9.86 per 100 person-years, respectively (Table 4).

For peer review only - http://bmjopen.bmj.com/site/about/guidelines.xhtml

Table 4: Rate of SRHR outcome by exposure to MTV Shuga (nested cohort 13-22 year olds n=2167)

**HSV-2 incidence rate**					
	person-time	n with HSV-2	Rate/ 100 py	[95% Conf.	Interval]
MTV Shuga Not exposed	1303.0	206	15.81	13.79	18.12
MTV Shuga Exposed	276.3	35	12.67	9.09	17.64
Total	1579.3	241	15.26	13.45	17.31
**Pregnancy incidence rates among all AGYW**					
	person-time	n pregnant	Rate/100 py	[95% Conf.	Interval]
MTV Shuga Not exposed	1068.7	104	9.73	8.03	11.79
MTV Shuga Exposed	188.5	20	10.61	6.84	16.44
Total	1257.2	124	9.86	8.27	11.76
**Pregnancy incidence rates among girls below 19 years**					
	person-time	n pregnant	Rate/100 py	[95% Conf.	Interval]
MTV Shuga Not exposed	668.7	55	8.22	6.31	10.71
MTV Shuga Exposed	123.9	17	13.72	8.53	22.07
Total	792.6	72	9.08	7.21	11.44

MTV Shuga and HIV prevention and SRHR awareness and uptake

In the nested cohort after adjusting for age, education, SES, area and DREAMS, MTV Shuga exposure in the AGYW cohort was associated with significantly greater awareness of PrEP (aOR=2.06, 95%CI: 1.57-2.70), contraception uptake (aOR=2.08, 95%CI: 1.45-2.98), and consistent condom use (aOR=1.84, 95%CI: 1.24-2.93). Watching MTV-Shuga was not associated with HIV testing (aOR=1.02,95%CI: 0.77-1.21) (Figure 3 and Supplementary Tables S1-S4). There was no effect modification by DREAMS exposure.

Figure 3 Forest plots showing the association between MTV Shuga exposure and HIV prevention and SRHR awareness and uptake in the nested cohorts.

Similarly, in the cross-sectional analysis, after adjusting for age, education, district, migration and sexual history, exposure to MTV-Shuga watching was associated with greater awareness of PrEP (aOR=1.70, 95%CI 1.20 - 2.43). However, there was no association with contraception, lower self-reported HIV testing, or condom use as shown in Figure 4 below (Supplementary Table 5).

For peer review only - http://bmjopen.bmj.com/site/about/guidelines.xhtml

Figure 4 Forest plots showing association between MTV Shuga exposure and HIV prevention and SRHR awareness and uptake in the cross-sectional surveys

Causal effect of MTV Shuga on health outcomes – HSV2

The causal analysis similarly finds no effect of MTV Shuga on HSV2, with a risk difference of 1.10 95% CI (-2.82- 5.38)%. Findings in the younger age group (aged 13-17) and the older age group (18-22) was similar (Supplementary Table 6).

Discussion

In our study of the population-level effect of a national broadcast of a TV-based edu-drama on HIV prevention and SRHR, exposure to MTV Shuga was associated with higher awareness of a novel HIV prevention intervention (PrEP). However, despite a very high incidence of HSV-2 and teenage pregnancy, MTV Shuga exposure was not significantly associated with safer sexual behaviour, uptake of contraception and HIV-testing or prevention of teenage pregnancy. Notably though, the size of the relationship and direction of effect between exposure to MTV Shuga, condom use and markers of unprotected sex (HSV-2 and pregnancy) was consistent with a possible relationship. These findings may be partly explained by our finding that less than one in 12 of the target age group had any exposure to MTV Shuga and only half of these had high exposure (defined as watching the MTV Shuga South African series and being able to recall the content), suggesting that one of the limiting factors for the effective use of TV-based edu-drama maybe the dose that young people are exposed to, particularly in rural and resource-constrained settings most affected by HIV.

Our inability to find a measurable population effect of mass-media edu-drama behaviour change campaign compared to the trial findings of the RCT is disappointing (18). However, firstly the direction and size of the relationships suggest that we may have been able to see an effect if the proportion exposed had been greater than 7%. Secondly, AGYW in urban settings were more likely to have been exposed to MTV Shuga and they are also more likely to be living in small towns and townships. Data from our settings suggest that young people in small towns and townships are more vulnerable to HIV (28) and sexual risk (29) and therefore it is possible that a real effect of MTV Shuga on this group was masked by their greater risk for the outcome. Due to the low numbers with high levels of exposure to MTV Shuga we did not have the power to explore this hypothesis by looking at effect modification by geography or socioeconomic status.

For peer review only - http://bmjopen.bmj.com/site/about/guidelines.xhtml

The finding of a differential association of MTV Shuga exposure with awareness of PrEP, compared to uptake of HIV and contraception, suggests that whilst educational mass entertainment may be able to increase awareness and possibly demand for a service, it does not impact on accessibility of the service: i.e. it impacts the first two steps of the prevention cascade and not the final step(30). Well described barriers to uptake of HIV-testing and contraception in this area are internalized and externalized stigma, fear of judgement from health care workers and the social costs of accessing care in busy primary health care settings(31-33). Behaviour change intervention including mass communication campaigns can be constrained or facilitated by the context in which people live (17, 34). To optimise MTV Shuga's effect there may need to be parallel innovations in SRHR and HIV service delivery that makes the services easier to access. We aim to test this hypothesis by providing community-based delivery of HIV and SRHR services in the context of the MTV Shuga.

The behaviour change theory that underpins edu-drama as a vehicle for mass behaviour change communication (16) explicitly suggests that the audience, and especially the early adopters, need to be actively watching, rather than passively watching or listening (17, 18). TV watching in rural homesteads can be in the context of large, often grandparent-led households and competing chores and priorities. This coupled with the relatively late timing of the shows may explain why so few girls and young women were sufficiently engaged or immersed to be able to recall characters or story lines. Moreover, the timing of this analysis may have allowed insufficient time for early adopters to convey the message of the show.

Limitations

The limitations of our study are that a meaningful exposure to MTV Shuga was low and so whilst the size of the relationship and direction of effect between exposure to MTV Shuga, condom use, and markers of unprotected sex (HSV-2 and pregnancy) suggested a possible effect we did not have the power to show a significant relationship between exposure to MTV Shuga and SRHR outcomes. We also do not have the power to see a difference by dose and immersion. Furthermore, as this is an observational study, we cannot exclude the possibility that those who are exposed to MTV Shuga are systematically different in ways that impact on the outcome of interest, for example more exposed to social media and sexual health promotion and innovative technologies to support sexual health than those who were not.

Conclusions and implications for the future

- Meaningful exposure to MTV Shuga was low across rural and urban settings in South Africa and so additional efforts need to be made to reach young people and

For peer review only - http://bmjopen.bmj.com/site/about/guidelines.xhtml

increase their immersion in promising edu-drama if it is to have the desired effect, especially in rural and deprived settings.

- MTV Shuga was an effective vehicle to raise awareness and promote newer HIV prevention technologies such as HIV PrEP.
- There was some suggestion that MTV Shuga improved uptake of some HIV prevention and sexual health technologies (contraception and condoms)
- There was less evidence from this observational study that it improved SRHR and HIV outcomes

We highlight the importance of evaluating the real-world scale up of promising interventions to understand both the reach and population effect as well as inform interventions to increase impact and equity.

Efforts to increase exposure, which have been rolled out as part of MTV Shuga in SA, such as social media, school-based or community-based MTV Shuga film clubs will need to be evaluated, both to understand whether or not they increase exposure and coverage and improve SRH and HIV outcomes. However, to have a significant impact on the HIV and SRH prevention and treatment cascades, demand generation in AGYW needs to be delivered in parallel with accessible service delivery models that support adherence and retention (30).

Contributorship

M.S., N.C., J.D., T.Z. N.K, G.H. and C.C. developed the study tools and performed the research. M.S., J.S. D.P., K.B., I.B., and S.F. designed the research study. T.S. and S.D. conducted the laboratory analysis. N.M. supported by K.B. conducted the statistical analysis and G.C. M.S wrote the first and final draft of the paper with input from N.C., N.M., G.H., J.S., I.B., G.C., K.B., C.C., T.S., T.Z, D.P and N.K. All the authors critically reviewed the manuscript. All authors have approved the final draft of the paper.

Competing interests

All authors declare they have no conflict of interest

Funding

The impact evaluation of DREAMS and MTV Shuga is funded by the Bill and Melinda Gates Foundation (OPP1136774 and OPP1171600, http://www.gatesfoundation.org). Foundation staff advised the study team, but did not substantively affect the study design, instruments,

For peer review only - http://bmjopen.bmj.com/site/about/guidelines.xhtml

interpretation of data, or decision to publish. The research leading to these results has received funding from the People Programme (Marie Curie Actions) of the European Union's seventh Framework Programme FP7/2007-2013 under REA grant agreement n° 612216. MS National Institutes of Health 5R01MH114560-03 funding acknowledgement. This research was funded in whole, or in part, by the Africa Health Research Institute through the Wellcome [Strategic Core award: 201433/Z/16/A]. For the purpose of open access, the author has applied a CC BY public copyright licence to any Author Accepted Manuscript version arising from this submission. N.C is supported by a training fellowship from the National Institute for Health Research (NIHR) (using the UK's Official Development Assistance (ODA) Funding) and Wellcome [grant reference number 224309/Z/21/Z] under the NIHR-Wellcome Partnership for Global Health Research. The views expressed are those of the authors and not necessarily those of Wellcome, the NIHR or the Department of Health and Social Care. GH is supported by a fellowship from the Royal Society and the Wellcome Trust [Grant number 210479/Z/18/Z]. MS work is also supported by a BMGF 3ie grant at AHRI. This research was funded in whole, or in part, by the Wellcome Trust [Grant number 210479/Z/18/Z]. For the purpose of open access, the author has applied a CC BY public copyright licence to any Author Accepted Manuscript version arising from this submission.

Data sharing statement

Data are available on reasonable request.

Ethics statement

The DREAMS Partnership impact evaluation protocol was reviewed and approved by the University of KwaZulu-Natal (UKZN) Biomedical Research Ethics Committee (BREC) (BFC 339/16), the London School of Hygiene & Tropical Medicine Research Ethics Committee (Ref 11835) and the AHRI Somkhele Community Advisory Board, the Associate Director of Science of the Center for Global Health (CGH) at the United States Centers for Disease Control and Prevention (CDC) in Atlanta and the Department of Health, Province of KZN and Gauteng. Approval for the demographic surveillance and data collection in the clinics was granted by UKZN BREC. All participants provided separate informed consent for the questionnaires and the HSV2 sero-survey. Consent for follow-up interviews was provided separately. For participants aged <18 years, written parental consent and participant assent were provided. Individuals attending the clinics in the surveillance area provided informed consent to record the clinic visit and link to their surveillance identification number.

For peer review only - http://bmjopen.bmj.com/site/about/guidelines.xhtml

Acknowledgements

The authors are grateful to the communities of the Hlabisa sub-district, Ekurhuleni district, Thekwini district and City of Johannesburg who contributed their data to this study and to all the staff at Africa Health Research Institute and EpiCentre who collected the data. We acknowledge Eskindir Shambullo for his input in this manuscript.

References

1. HSRC. The fifth South African National HIV prevalence, incidence, behaviour and communication survey [Report]. http://www.hsrc.ac.za/uploads/pageContent/9234/SABSSMV_Impact_Assessment_Summary_ZA_ADS_cleared_PDFA4.pdf: South African Health Services Research Council 2017 [updated 01/11/2018.
2. Chimbindi N, Mthiyane N, Birdthistle I, Floyd S, McGrath N, Pillay D, et al. Persistently high incidence of HIV and poor service uptake in adolescent girls and young women in rural KwaZulu-Natal, South Africa prior to DREAMS. PLoS One. 2018;13(10):e0203193.
3. Vandormael A, Akullian A, Siedner M, de Oliveira T, Bärnighausen T, Tanser F. Declines in HIV incidence among men and women in a South African population-based cohort. Nature Communications. 2019;10(1):5482.
4. Birdthistle I, Kwaro D, Shahmanesh M, Baisley K, Khagayi S, Chimbindi N, et al. Evaluating the impact of DREAMS on HIV incidence among adolescent girls and young women: A population-based cohort study in Kenya and South Africa. PLoS medicine. 2021;18(10):e1003837.
5. Department of Health RoSA. National Campaign for Young Women and Adolescent Girls. . 2016.
6. Saul J, Bachman G, Allen S, Toiv NF, Cooney C, Beamon TA. The DREAMS core package of interventions: A comprehensive approach to preventing HIV among adolescent girls and young women. PLOS ONE. 2018;13(12):e0208167.
7. PEPFAR. Preventing HIV in Adolescent Girls and Young Women: Guidance for PEPFAR Country Teams on the DREAMS Partnership 2015.
8. PEPFAR. South Africa DREAMS overview https://www.pepfar.gov/documents/organization/253958.pdf: PEPFAR; 2017 [
9. Chang L, Serwadda D, Quinn T, Wawer M, Gray R, Reynolds S. Combination implementation for HIV prevention: moving from clinical trial evidence to population-level effects The Lancet Infectious Diseases 2013;13(1):65-76.

For peer review only - http://bmjopen.bmj.com/site/about/guidelines.xhtml

10. Iwuji CC, Orne-Gliemann J, Larmarange J, Balestre E, Thiebaut R, Tanser F, et al. Universal test and treat and the HIV epidemic in rural South Africa: a phase 4, open-label, community cluster randomised trial. Lancet HIV. 2018;5(3):e116-e25.

11. Iwuji CC, Orne-Gliemann J, Tanser F, Boyer S, Lessells RJ, Lert F, et al. Evaluation of the impact of immediate versus WHO recommendations-guided antiretroviral therapy initiation on HIV incidence: the ANRS 12249 TasP (Treatment as Prevention) trial in Hlabisa sub-district, KwaZulu-Natal, South Africa: study protocol for a cluster randomised controlled trial. Trials. 2013;14:230.

12. Birdthistle I, Schaffnit SB, Kwaro D, Shahmanesh M, Ziraba A, Kabiru CW, et al. Evaluating the impact of the DREAMS partnership to reduce HIV incidence among adolescent girls and young women in four settings: a study protocol. BMC Public Health. 2018;18(1):912.

13. Shen F, Han J. Effectiveness of entertainment education in communicating health information: a systematic review. Asian Journal of Communication. 2014;24(6):605-16.

14. Noar SM, Palmgreen P, Chabot M, Dobransky N, Zimmerman RS. A 10-year systematic review of HIV/AIDS mass communication campaigns: have we made progress? Journal of Health Communication. 2009;14(1):15-42.

15. Kyegombe N, Zuma T, Hlongwane S, Nhlenyama M, Chimbindi N, Birdthistle I, et al. A qualitative exploration of the salience of MTV-Shuga, an edutainment programme, and adolescents' engagement with sexual and reproductive health information in rural KwaZulu-Natal, South Africa. Sexual and Reproductive Health Matters. 2022;30(1):2083809.

16. Barker K. Sex Soap and Social change- The Sabedo methadology https://www.populationmedia.org/product/sabido-theory/; http://www.comminit.com/content/sabido-methodology [

17. Baker V, Birdthistle I, Seeley J, Arnold G, Piot S, Hajiyiannis H. How can a TV drama about Love, Sex and Growing up Increase Knowledge and Shape the Sexual Health Behaviours of Young People? . AIDS Impact; Cape Town, South Africa2017.

18. MTV Shuga Soap Opera Turns Edutainment into a Tool to Fight HIV and Gender-Based Violence http://www.worldbank.org/en/news/feature/2017/02/23/mtv-shuga-soap-opera-turns-edutainment-into-a-tool-to-fight-hiv-and-gender-based-violence2017 [

19. National Department of Health. The National Antenatal Sentinel HIV prevalence survey, South Africa, http://www.health.gov.za/index.php/shortcodes/2015-03-29-10-42-47/2015-04-30-08-18-10/2015-04-30-08-21-56?download=2584:2015-national-antenatal-hiv-prevalence-survey-final-23oct17, 2013 [

For peer review only

20. Herbst K, Law M, Geldsetzer P, Tanser F, Harling G, Barnighausen T. Innovations in health and demographic surveillance systems to establish the causal impacts of HIV policies. Current Opinion on HIV AIDS. 2015;10(6):483-94.

21. Gareta D, Baisley K, Mngomezulu T, Smit T, Khoza T, Nxumalo S, et al. Cohort Profile Update: Africa Centre Demographic Information System (ACDIS) and population-based HIV survey. Int J Epidemiol. 2021;50(1):33-4.

22. George G, Cawood C, Puren A, Khanyile D, Gerritsen A, Govender K, et al. Evaluating DREAMS HIV prevention interventions targeting adolescent girls and young women in high HIV prevalence districts in South Africa: protocol for a cross-sectional study. BMC Women's Health. 2020;20(1):7.

23. Filmer D, Pritchett LH. Estimating Wealth Effects Without Expenditure Data—Or Tears: An Application To Educational Enrollments In States Of India*. Demography. 2001;38:115-32.

24. Arvin A, Prober C. Herpes Simplex Viruses. In: Murray P, Baron E, Pfaller M, Tenover F, Yolkenet R, editors. Manual of Clinical Microbiology 6th Ed Washington D.C.: ASM; 1995. p. 876-83.

25. Delany-Moretlwe S, Jentsch U, Weiss H, Moyes J, Ashley-Morrow R, Stevens W, et al. Comparison of Focus HerpeSelect ® and Kalon TM HSV-2 gG2 ELISA serological assays to detect herpes simplex virus type 2 (HSV-2) antibodies in a South African population. . Sexually Transmitted Infections,. 2009;86(1):46-50.

26. Ashley-Morrow R, Nolkamper J, Robinson N, Bishop N, Smith J. Performance of Focus ELISA test for herpes simplex virus type 1 (HSV-1) and HSV-2 antibodies among women in ten diverse geographical locations. . Clinical Microbiology and Infection 2004;10(6):530-6.

27. STROBE Statement. STROBE Checklists 2007 [Available from: http://www.strobe-statement.org/index.php?id=available-checklists.

28. Cuadros DF, Li J, Branscum AJ, Akullian A, Jia P, Mziray EN, et al. Mapping the spatial variability of HIV infection in Sub-Saharan Africa: Effective information for localized HIV prevention and control. Scientific Reports. 2017;7(1):9093.

29. Ngwenya N, Gumede D, Shahmanesh M, McGrath N, Grant A, Seeley J. Community perceptions of the socio-economic structural context influencing HIV and TB risk, prevention and treatment in a high prevalence area in the era of antiretroviral therapy. African Journal of AIDS Research. 2018;17(1):72-81.

30. Hargreaves JR, Delany-Moretlwe S, Hallett TB, Johnson S, Kapiga S, Bhattacharjee P, et al. The HIV prevention cascade: integrating theories of epidemiological, behavioural, and social science into programme design and monitoring. Lancet HIV. 2016;3(7):e318-22.

For peer review only - http://bmjopen.bmj.com/site/about/guidelines.xhtml

31. Hargreaves JR, Krishnaratne S, Mathema H, Lilleston PS, Sievwright K, Mandla N, et al. Individual and community-level risk factors for HIV stigma in 21 Zambian and South African communities: analysis of data from the HPTN071 (PopART) study. AIDS. 2018;32(6):783-93.

32. Chimbindi N, Zuma T, Nakasone S, Birdthistle I, Mthiyane N, Dreyer J, et al. Platforms, Processes and Perceptions of PrEP Delivery Through DREAMS in a Rural Setting in KwaZulu-Natal, South Africa. . Research for Prevention 2018 (HIVR4P 2018) Madrid, Spain 2018.

33. Adeagbo O, Herbst C, Dlamini N, Mhlongo T, Luthuli M, Xulu S, et al. "If she tests negative, it means I am also negative": Men's construction of HIV testing in KwaZulu-Natal, South Africa. . HIV Research for Prevention (HIVR4P) 23 October 2018.; Madrid, Spain **2018**

34. Michie S, van Stralen MM, West R. The behaviour change wheel: A new method for characterising and designing behaviour change interventions. Implementation Science. 2011;6(42).

For peer review only

Figure legends

Figure 1 showing conceptual framework for MTV Shuga impact on HIV prevention on AGYW in uMkhanyakude

Figure 2 Flowchart showing AGYW follow-up from nested cohorts 2017-2019

Figure 3 Forest plots showing the association between MTV Shuga exposure and HIV prevention and SRHR awareness and uptake in the nested cohorts.

Figure 4 Forest plots showing association between MTV Shuga exposure and HIV prevention and SRHR awareness and uptake in the cross-sectional surveys

For peer review only

For peer review only - http://bmjopen.bmj.com/site/about/guidelines.xhtml

Figure 1: Conceptual framework for MTV Shuga impact on HIV prevention in uMkhanyakude

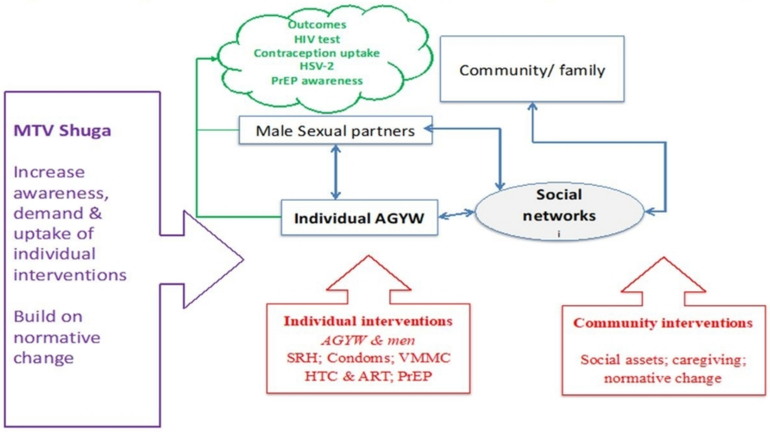

Figure 1 showing conceptual framework for MTV Shuga impact on HIV prevention on AGYW in uMkhanyakude

98x51mm (300 x 300 DPI)

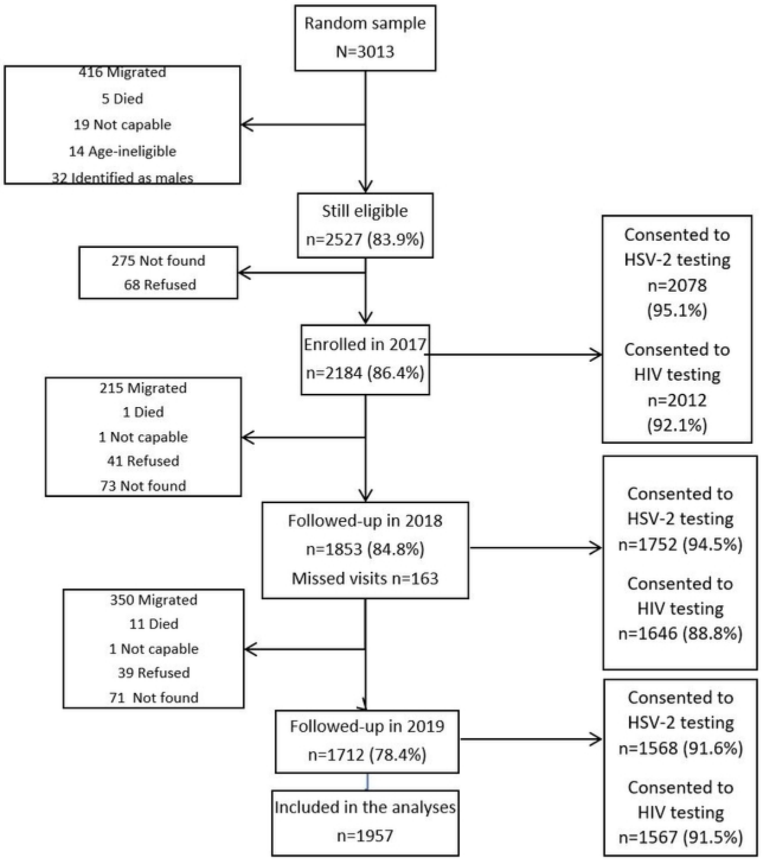

Figure 2 Flowchart showing AGYW follow-up from nested cohorts 2017-2019

71x64mm (300 x 300 DPI)

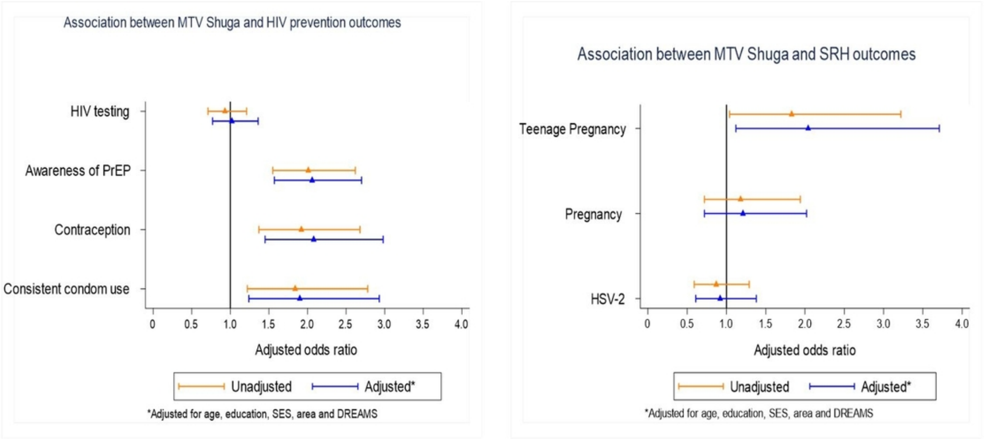

Figure 3 Forest plots showing the association between MTV Shuga exposure and HIV prevention and SRHR awareness and uptake in the nested cohorts.

95x46mm (300 x 300 DPI)

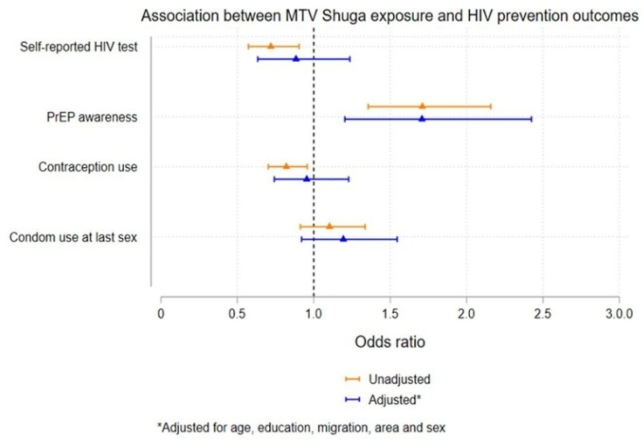

Figure 4 Forest plots showing association between MTV Shuga exposure and HIV prevention and SRHR awareness and uptake in the cross-sectional surveys

101x54mm (300 x 300 DPI)

Supplementary Table 1 Exposure to MTV Shuga and PrEP awareness in the nested cohort of AGYW aged 13-22 (n=2184)

	Unadjusted		Shuga adjusted			Adjusted-All		
	OR	95%CI	OR	95%CI	p-value	OR	95%CI	p-value
**Ever watched MTV Shuga, 2018/19**								
No	1					1		
Yes	2.01	1.55 -2.62				2.06	1.57 -2.70	<0.001
**Age**	1.12	1.08-1.16	1.12	1.08-1.17	<0.001	1.11	1.06-1.17	<0.001
**Currently in school**								
No	1		1			1		
Yes	0.6	0.47 -0.76	0.59	0.46 -0.75	<0.001	0.89	0.65 -1.23	0.488
**Socio-economic status, 2018**								
Low	1		1			1		
Middle	0.99	0.71 -1.39	0.96	0.68 -1.35		1.01	0.71 -1.44	
High	1.13	0.81 -1.58	1.05	0.75 -1.47	0.744	1.11	0.78 -1.58	0.699
**Urban or rural**								
Rural	1		1			1		
Peri-urban/urban	0.98	0.79 -1.22	0.93	0.75 -1.16	0.525	0.92	0.73 -1.15	0.459
**Invited or received DREAMS, 2017/18**								
No	1		1			1		
Yes	0.89	0.73 -1.10	0.88	0.71 -1.08	0.212	0.99	0.79 -1.23	0.897

For peer review only

Supplementary Table 2 Exposure to MTV Shuga and contraception uptake in the nested cohort of AGYW aged 13-22 (n=2184)

	Unadjusted		Shuga adjusted			Adjusted-All		
	OR	95%CI	OR	95%CI	p-value	OR	95%CI	p-value
**Ever watched MTV Shuga, 2018/19**								
No	1					1		
Yes	1.92	1.37 -2.68				2.08	1.45-2.98	<0.001
**Age**	1.26	1.19-1.33	1.27	1.20-1.34	<0.001	1.32	1.24-1.42	<0.001
**Currently in school**								
No	1		1			1		
Yes	0.6	0.44 -0.83	0.59	0.43-0.82	0.001	1.7	1.13-2.55	0.01
**Socio-economic status, 2018**								
Low	1		1			1		
Middle	0.79	0.51 -1.22	0.76	0.49-1.19		0.8	0.51-1.28	
High	0.86	0.55 -1.34	0.8	0.51-1.24	0.489	0.83	0.52-1.32	0.646
**Urban or rural**								
Rural	1		1			1		
Peri-urban/urban	0.91	0.68 -1.24	0.86	0.64-1.17	0.345	0.89	0.64-1.24	0.49
**Invited or received DREAMS, 2017/18**								
No	1		1			1		
Yes	0.84	0.63 -1.11	0.83	0.62-1.10	0.191	1.04	0.76-1.42	0.813

For peer review only

Supplementary Table 3 Exposure to MTV Shuga and consistent condom use in the nested cohort of AGYW aged 13-22 (n=2184)

	Unadjusted		Shuga adjusted			Adjusted-All		
	**OR**	**95%CI**	**OR**	**95%CI**	**p-value**	**OR**	**95%CI**	**p-value**
**Ever watched MTV Shuga, 2018/19**								
No	1					1		
Yes	1.84	1.22-2.78				1.9	1.24-2.93	0.003
**Age**	0.94	0.86-1.01	0.94	0.87-1.02	0.157	1	0.91-1.10	0.984
**Currently in school**								
No	1		1			1		
Yes	1.76	1.22-2.53	1.73	1.20-2.49	0.003	1.78	1.15-2.76	0.01
**Socio-economic status, 2018**								
Low	1		1			1		
Middle	1.01	0.60-1.70	0.97	0.58-1.63		0.98	0.58-1.67	
High	0.87	0.52-1.47	0.8	0.47-1.36	0.552	0.86	0.50-1.48	0.753
**Urban or rural**								
Rural	1		1			1		
Peri-urban/urban	0.7	0.48-1.02	0.67	0.46-0.97	0.035	0.71	0.48-1.05	0.088
**Invited or received DREAMS, 2017/18**								
No	1		1			1		
Yes	1.13	0.81-1.59	1.13	0.80-1.59	0.486	0.97	0.67-1.40	0.875

For peer review only

Supplementary Table 4 Exposure to MTV Shuga and HIV testing in the nested cohort of AGYW aged 13-22 (n=2184)

	Unadjusted		Shuga adjusted			Adjusted-All		
	**OR**	**95%CI**	**OR**	**95%CI**	**p-value**	**OR**	**95%CI**	**p-value**
**Ever watched MTV Shuga, 2018/19**								
No	1					1		
Yes	0.93	0.71-1.21				1.02	0.77-1.36	0.885
**Age**	1.3	1.25-1.35	1.3	1.24-1.35	<0.001	1.3	1.24-1.37	<0.001
**Currently in school**								
No	1		1			1		
Yes	0.37	0.28-0.48	0.37	0.28-0.48	0	1.07	0.74-1.53	0.726
**Socio-economic status, 2018**								
Low	1		1			1		
Middle	0.67	0.48-0.94	0.67	0.48-0.95		0.71	0.49-1.02	
High	0.58	0.41-0.81	0.58	0.41-0.81	0.007	0.6	0.41-0.86	0.018
**Urban or rural**								
Rural	1		1			1		
Peri-urban/urban	0.84	0.68-1.03	0.84	0.69-1.04	0.104	0.89	0.71-1.11	0.298
**Invited or received DREAMS, 2017/18**								
No	1		1			1		
Yes	0.84	0.69-1.02	0.84	0.69-1.02	0.081	0.98	0.79-1.23	0.89

For peer review only

Supplementary Table 5: Exposure to MTV Shuga with HIV prevention and SRHR awareness and uptake in the cross-sectional analysis of AGYW aged 12-24 (n=4127)

	aOR (95% Confidence Interval)	P-value
**District**		
City of Johannesburg	1	
Ekurhuleni	0.66 (0.47-0.91)	0.013
eThekwini	0.78 (0.54-1.13)	0.186
**Age group**		
12-14	1	
15-19	1.75 (0.35-8.84)	0.499
20-24	1.60 (0.32-8.07)	0.570
**Highest education level**		
Grade R to 7	1	
No schooling	3.84 (1.05-14.02)	0.041
Grade 8 to 12	3.17 (0.93-10.77)	0.065
Complete or incomplete tertiary	6.52 (1.90-22.33)	0.003
**Ever had sex with a boy/man**		
No	1	
Yes	0.67 (0.32-1.40)	0.286
**Away from home**		
No	1	
Yes	1.48 (0.93-2.37)	0.099
**PrEP awareness**		
No	1	
Yes	1.71 (1.20-2.43)	0.003
**Contraception use**		
No	1	
Yes	0.95 (0.74-1.23)	0.718
**Condom use at last sex**		
No	1	
Yes	1.19 (0.92-1.55)	0.183
**HIV test (self-report)**		
No	1	
Yes	0.88 (0.63-1.24)	0.471

For peer review only

Supplementary Table 6 Estimated causal effect of MTV Shuga on HSV-2 incidence, overall and by age group

Estimated causal effect of MTV Shuga on HSV-2 incidence, overall and by age group											
	% Outcome in total study population	Estimated % Outcome if no Shuga	95% CI	Estimated % Outcome if all get Shuga	95% CI	Risk Difference (%; PS adjusted)	95% CI	Prevalence Ratio (%; PS adjusted)	95% CI	Odds Ratio (%; PS adjusted)	95% CI
PS adjustment: Primary results											
Overall	17.3	17.2	14.71 - 19.67	18.0	14.51 - 22.32	0.80	-3.24 - 5.71	1.05	0.82 - 1.36	1.06	0.79 - 1.46
13-17 Years	13.2	12.4	9.85 - 15.16	15.7	11.63 - 20.26	3.34	-1.60 - 8.25	1.27	0.89 - 1.79	1.32	0.87 - 1.98
18-22 Years	24.3	25.8	21.01 - 30.74	22.0	15.19 - 30.17	-3.76	-11.92 - 5.68	0.85	0.59 - 1.24	0.81	0.51 - 1.34
**Sensitivity analyses**											
Regression under counterfactual framework											
Overall	17.3	17.2	14.74 - 19.66	17.8	14.50 - 22.08	0.65	-3.42 - 5.72	1.04	0.82 - 1.36	1.05	0.78 - 1.46
13-17 Years	13.2	12.42	9.86 - 15.21	15.9	11.85 - 20.49	3.47	-1.54 - 8.63	1.28	0.89 - 1.80	1.33	0.88 - 1.99
18-22 Years	24.3	25.7	20.78 - 30.63	21.3	14.88 - 28.28	-4.42	-12.28 - 4.95	0.83	0.57 - 1.22	0.78	0.48 - 1.31
PS stratification											
Overall	17.3	17.1	14.68 - 19.68	17.9	14.53 - 22.13	0.80	-3.21 - 5.52	1.09	0.84 - 1.40	1.11	0.82 - 1.51
PS weighting											

For peer review only

Overall	17.3	17.08	14.74 - 19.48	17.22	13.99 - 21.10	0.13	-3.76 - 4.63	1.01	0.79 - 1.30	1.01	0.76 - 1.37

For peer review only

STROBE Statement—checklist of items that should be included in reports of observational studies

	Item No	Recommendation	Page No
**Title and abstract**	1	(*a*) Indicate the study's design with a commonly used term in the title or the abstract	1
		(*b*) Provide in the abstract an informative and balanced summary of what was done and what was found	2
**Introduction**			
Background/rationale	2	Explain the scientific background and rationale for the investigation being reported	4
Objectives	3	State specific objectives, including any prespecified hypotheses	6
**Methods**			
Study design	4	Present key elements of study design early in the paper	6-7
Setting	5	Describe the setting, locations, and relevant dates, including periods of recruitment, exposure, follow-up, and data collection	7
Participants	6	(*a*) *Cohort study*—Give the eligibility criteria, and the sources and methods of selection of participants. Describe methods of follow-up<br>*Case-control study*—Give the eligibility criteria, and the sources and methods of case ascertainment and control selection. Give the rationale for the choice of cases and controls<br>*Cross-sectional study*—Give the eligibility criteria, and the sources and methods of selection of participants	7
		(*b*) *Cohort study*—For matched studies, give matching criteria and number of exposed and unexposed<br>*Case-control study*—For matched studies, give matching criteria and the number of controls per case	
Variables	7	Clearly define all outcomes, exposures, predictors, potential confounders, and effect modifiers. Give diagnostic criteria, if applicable	8-9
Data sources/ measurement	8*	For each variable of interest, give sources of data and details of methods of assessment (measurement). Describe comparability of assessment methods if there is more than one group	8-10
Bias	9	Describe any efforts to address potential sources of bias	9-10
Study size	10	Explain how the study size was arrived at	6-7
Quantitative variables	11	Explain how quantitative variables were handled in the analyses. If applicable, describe which groupings were chosen and why	8
Statistical methods	12	(*a*) Describe all statistical methods, including those used to control for confounding	9-10
		(*b*) Describe any methods used to examine subgroups and interactions	10
		(*c*) Explain how missing data were addressed	10
		(*d*) *Cohort study*—If applicable, explain how loss to follow-up was addressed<br>*Case-control study*—If applicable, explain how matching of cases and controls was addressed<br>*Cross-sectional study*—If applicable, describe analytical methods taking account of sampling strategy	10
		(*e*) Describe any sensitivity analyses	10

Continued on next page

For peer review only - http://bmjopen.bmj.com/site/about/guidelines.xhtml

**Results**			
Participants	13*	(a) Report numbers of individuals at each stage of study—eg numbers potentially eligible, examined for eligibility, confirmed eligible, included in the study, completing follow-up, and analysed	11
		(b) Give reasons for non-participation at each stage	11
		(c) Consider use of a flow diagram	11
Descriptive data	14*	(a) Give characteristics of study participants (eg demographic, clinical, social) and information on exposures and potential confounders	11
		(b) Indicate number of participants with missing data for each variable of interest	
		(c) *Cohort study*—Summarise follow-up time (eg, average and total amount)	
Outcome data	15*	*Cohort study*—Report numbers of outcome events or summary measures over time	12
		Case-control study—Report numbers in each exposure category, or summary measures of exposure	
		Cross-sectional study—Report numbers of outcome events or summary measures	12
Main results	16	(*a*) Give unadjusted estimates and, if applicable, confounder-adjusted estimates and their precision (eg, 95% confidence interval). Make clear which confounders were adjusted for and why they were included	12-16
		(*b*) Report category boundaries when continuous variables were categorized	12-16
		(*c*) If relevant, consider translating estimates of relative risk into absolute risk for a meaningful time period	
Other analyses	17	Report other analyses done—eg analyses of subgroups and interactions, and sensitivity analyses	17
**Discussion**			
Key results	18	Summarise key results with reference to study objectives	17-18
Limitations	19	Discuss limitations of the study, taking into account sources of potential bias or imprecision. Discuss both direction and magnitude of any potential bias	18
Interpretation	20	Give a cautious overall interpretation of results considering objectives, limitations, multiplicity of analyses, results from similar studies, and other relevant evidence	18-19
Generalisability	21	Discuss the generalisability (external validity) of the study results	17-19
**Other information**			
Funding	22	Give the source of funding and the role of the funders for the present study and, if applicable, for the original study on which the present article is based	20

*Give information separately for cases and controls in case-control studies and, if applicable, for exposed and unexposed groups in cohort and cross-sectional studies.

Note: An Explanation and Elaboration article discusses each checklist item and gives methodological background and published examples of transparent reporting. The STROBE checklist is best used in conjunction with this article (freely available on the Web sites of PLoS Medicine at http://www.plosmedicine.org/, Annals of Internal Medicine at http://www.annals.org/, and Epidemiology at http://www.epidem.com/). Information on the STROBE Initiative is available at www.strobe-statement.org.

For peer review only - http://bmjopen.bmj.com/site/about/guidelines.xhtml

BMJ Open

Evaluating use of mass-media communication intervention 'MTV-Shuga' on increased awareness and demand for HIV and sexual health services by adolescent girls and young women in South Africa: An observational study

Journal:	*BMJ Open*
Manuscript ID	bmjopen-2022-062804.R2
Article Type:	Original research
Date Submitted by the Author:	03-Jan-2023

Complete List of Authors:	Chimbindi, Natsayi; Africa Health Research Institute; University College London,<br>Mthiyane, Nondumiso; Africa Health Research Institute<br>Chidumwa, Glory ; Africa Health Research Institute<br>Zuma, Thembelihle; Africa Health Research Institute; University College London,<br>Dreyer, Jaco; Africa Health Research Institute<br>Birdthistle, Isolde; London School of Hygiene and Tropical Medicine Faculty of Epidemiology and Population Health, Department of Population Health<br>Floyd, Sian; London School of Hygiene & Tropical Medicine, Infectious Disease Epidemiology<br>Kyegombe, Nambusi; London School of Hygiene & Tropical Medicine<br>Grundy, Chris; London School of Hygiene and Tropical Medicine Faculty of Epidemiology and Population Health, Department of Population Health<br>Cawood, Cherie; Epicentre Health Research<br>Danaviah, Siva; Africa Health Research Institute<br>Smit, Theresa; Africa Health Research Institute<br>Pillay, Deenan; University College London,<br>Baisley, Kathy; London School of Hygiene and Tropical Medicine Faculty of Epidemiology and Population Health; Africa Health Research Institute<br>Harling, Guy; Africa Health Research Institute<br>Seeley, Janet; London School of Hygiene and Tropical Medicine, Department of Global Health &Development; Africa Health Research Institute<br>Shahmanesh, Maryam; University College London, Institute for Global Health; Africa Health Research Institute,

**Primary Subject Heading**:	Sexual health
Secondary Subject Heading:	HIV/AIDS, Public health, Sexual health
Keywords:	HIV & AIDS < INFECTIOUS DISEASES, PUBLIC HEALTH, PREVENTIVE MEDICINE

For peer review only - http://bmjopen.bmj.com/site/about/guidelines.xhtml

I, the Submitting Author has the right to grant and does grant on behalf of all authors of the Work (as defined in the below author licence), an exclusive licence and/or a non-exclusive licence for contributions from authors who are: i) UK Crown employees; ii) where BMJ has agreed a CC-BY licence shall apply, and/or iii) in accordance with the terms applicable for US Federal Government officers or employees acting as part of their official duties; on a worldwide, perpetual, irrevocable, royalty-free basis to BMJ Publishing Group Ltd ("BMJ") its licensees and where the relevant Journal is co-owned by BMJ to the co-owners of the Journal, to publish the Work in this journal and any other BMJ products and to exploit all rights, as set out in our licence.

The Submitting Author accepts and understands that any supply made under these terms is made by BMJ to the Submitting Author unless you are acting as an employee on behalf of your employer or a postgraduate student of an affiliated institution which is paying any applicable article publishing charge ("APC") for Open Access articles. Where the Submitting Author wishes to make the Work available on an Open Access basis (and intends to pay the relevant APC), the terms of reuse of such Open Access shall be governed by a Creative Commons licence – details of these licences and which Creative Commons licence will apply to this Work are set out in our licence referred to above.

Other than as permitted in any relevant BMJ Author's Self Archiving Policies, I confirm this Work has not been accepted for publication elsewhere, is not being considered for publication elsewhere and does not duplicate material already published. I confirm all authors consent to publication of this Work and authorise the granting of this licence.

For peer review only

Title page

Revised Title: **Evaluating use of mass-media communication intervention 'MTV-Shuga' on increased awareness and demand for HIV and sexual health services by adolescent girls and young women in South Africa: An observational study**

Authors: Natsayi Chimbindi*[1,2,3], Nondumiso Mthiyane*[2], Glory Chidumwa[1], Thembelihle Zuma[1,2,3], Jaco Dreyer[1], Isolde Birdthistle[4], Sian Floyd[4], Nambusi Kyegombe[4], Chris Grundy[4], Cherie Cawood[5], Siva Danaviah[1], Theresa Smit[1], Deenan Pillay[2], Kathy Baisley[1,3], Guy Harling[1,2,3,6,7], Janet Seeley[1,3,4], Maryam Shahmanesh§[1,2,3]

1. Africa Health Research Institute, KwaZulu-Natal, South Africa
2. University College London, Institute for Global Health, London, UK
3. University of KwaZulu-Natal, KwaZulu-Natal, South Africa
4. London School of Hygiene and Tropical Medicine, London, UK
5. Epicentre, South Africa
6. MRC/Wits Rural Public Health & Health Transitions Research Unit (Agincourt), University of the Witwatersrand, Gauteng, South Africa
7. Department of Epidemiology & Harvard Center for Population and Development Studies, Harvard T.H. Chan School of Public Health, Boston, USA

*Joint authors

§Corresponding author (full address details): Maryam Shahmanesh, Institute for Global Health, UCL, 3rd Floor, Mortimer Market Centre, Capper Street, London WC1E 6JB. m.shahmanesh@ucl.ac.uk. +44 7776 185 572

Key words (6): HIV, Adolescents, mass communication campaigns, Sexual Health, Teenage Pregnancy, South Africa.

For peer review only

Abstract (word count=272)

Objective: To investigate the effect of exposure to MTV Shuga:Down South' (MTVShuga-DS) during the scale-up of combination HIV-prevention interventions on awareness and uptake of SRH and HIV-prevention services by adolescent girls and young women (AGYW).

Design: One longitudinal and three cross-sectional surveys of representative samples of AGYW.

Setting: AGYW in four South African districts with high HIV-prevalence (>10%) (May 2017 and September 2019).

Participants: 6311 AGYW aged 12-24.

Measures: Using logistic-regression we measured the relationship between exposure to MTV Shuga-DS and awareness of Pre-Exposure Prophylaxis (PrEP), condom-use at last sex, uptake of HIV-testing or contraception, and incident pregnancy or HSV-2 infection.

Results: Within the rural cohort 2184 (85.5%) of eligible sampled individuals were enrolled, of whom 92.6% had at least one follow-up visit; the urban cross-sectional surveys enrolled 4127 (22.6%) of eligible sampled individuals. Self-report of watching at least one MTV Shuga-DS episode was 14.1% (cohort) and 35.8% (cross-section), while storyline recall was 5.5% (cohort) and 6.7% (cross-section).

In the cohort, after adjustment (for HIV-prevention intervention-exposure, age, education, SES), MTVShuga-DS exposure was associated with increased PrEP awareness (aOR=2.06,95%CI:1.57-2.70), contraception uptake (aOR=2.08,95%CI:1.45-2.98) and consistent condom-use (aOR=1.84, 95%CI:1.24-2.93), but not with HIV-testing (aOR=1.02,95%CI:0.77-1.21) or acquiring HSV-2 (aOR=0.92, 95%CI:0.61-1.38). In the cross-sections, MTVShuga-DS was associated with greater PrEP awareness (aOR=1.7, 95%CI:1.20-2.43), but no other outcome.

Conclusions: Among both urban and rural AGYW in South Africa, MTVShuga-DS exposure was associated with increased PrEP awareness and improved demand for some HIV-prevention and SRH technologies but not sexual health outcomes. However, exposure to MTVShuga-DS was low. Given these positive indications, supportive programming may be required to raise exposure and allow future evaluation of edu-drama impact in this setting.

For peer review only - http://bmjopen.bmj.com/site/about/guidelines.xhtml

Strengths and limitations of this study

- Evaluated the real-world reach of nationally broadcast edu-drama focusing on adolescent sexual health in South Africa.
- Data collection focused on a vulnerable population of AGYW across four diverse high HIV-burden districts of South Africa that included both rural and urban settings.
- The strength of this study is the use of longitudinal data from a cohort of AGYW nested in a larger population-based longitudinal HIV surveillance. In addition data were drawn from cross-sectional representative samples of AGYW from four districts of South Africa. These data enabled us to measure the reach of MTV-Shuga and the relationship between exposure to the edu-drama on awareness and demand for HIV prevention technologies in young women.
- The limitation of observational studies is that they do not infer the cause–effect relationship, in this case we cannot ascertain causality/impact of exposure to MTV-Shuga on uptake of sexual health promotion and innovative technologies.

For peer review only

For peer review only - http://bmjopen.bmj.com/site/about/guidelines.xhtml

[word count 4531]

Introduction

HIV remains one of the gravest health problems facing young people living in sub-Saharan Africa (SSA). There are over 7.7 million people living with HIV in South Africa, with more than 200,000 new HIV infections annually in those aged 15-49 years (1). The highest incidence is in adolescent girls and young women (AGYW) (15-24 years) (1, 2). Although HIV incidence has been declining in South Africa, a 43% decline in the overall incidence rate between 2012 and 2017, from 4.0 to 2.3 seroconversion events per 100 person-years among 15-49 year old; it still remains high among AGYW in South Africa (3). In uMkhanyakude, HIV incidence was lower during roll-out of combination HIV prevention for AGYW (2016 to 2018) than in the previous 5-year period among 15- to 19-year-old females (4.5 new infections per 100 person-years as compared with 2.8; and lower among 20- to 24-year-olds (7.1/100 person-years as compared with 5.8) (4).

In response to the high HIV incidence in young people, the South African government launched the `She Conquers Campaign', and the US President's Emergency Fund for AIDS Relief and others are supporting the roll-out of Determined, Resilient, Empowered, AIDS free, Mentored, and Safe (DREAMS)(5-7). These programmes provide an evidence-based combination HIV prevention package, including HIV-testing and counselling for adolescent girls and young women (AGYW) and their male sexual partners, alongside universal test and treat and improved sexual health services (8, 9).

However, the key ingredient to the success of these multicomponent interventions will be the extent to which AGYW and their male partners at most risk of HIV will uptake and adhere to the active components of the intervention. This is challenging: uptake and retention of contraception and HIV treatment cascade by young people, even within population-wide Universal Test and Treat trials, has been suboptimal (2, 10, 11). Data from the baseline analysis for the DREAMS impact evaluation in uMkhanyakude district, rural KwaZulu-Natal (KZN) in 2015 suggest that less than 40% of girls (15-19 years) and boys and young men (15-29 years) had ever tested for HIV; linkage to HIV treatment was even lower (12). Contraception use prevalence was 20% in girls (15-19 years) and 50% in young women (20-24 years) and 21% of 15-19 year old girls had ever been pregnant (2).

It is against this backdrop that the fifth series of Shuga: 'MTV Shuga: Down South' (MTV Shuga-DS), a mass media serial edu-drama designed for SA, was broadcast on free-to-air South Africa National television. From March 8th 2017, MTV Shuga aired one episode per week for 12 weeks (with repeats). MTV Shuga is a mass media behaviour change campaign

For peer review only - http://bmjopen.bmj.com/site/about/guidelines.xhtml

that aims to improve sexual and reproductive health rights (SRHR). At the centre of the campaign, which includes radio and social media, is a TV-drama that weaves messages about HIV, family planning, transactional and intergenerational sex, sexual identity, safer and healthy sexual relationships, into storylines with young characters (http://www.mtvshuga.com/show/series-5/MTV Shuga-down-south/).

Mass media campaigns, have the potential to reach a large number of people and have been shown to improve knowledge and health behaviour of a range of health conditions, with more recent data suggesting that theoretically informed and targeted interventions are more likely to have an effect (13, 14). MTV Shuga-DS was designed to reduce HIV-related risk behaviour and improve SRHR outcomes in adolescents and young adults in SA. This was expected to be achieved through increasing young people's awareness of their sexual and reproductive health rights and demand for, and uptake of HIV and SRH prevention and treatment technologies. The show's characters explicitly model how to discuss issues that are sensitive or taboo. MTV Shuga use the technique of 'melodramas', where drama is created through the battles between stereotypical goodies and baddies, and the way in which the 'transitional' (often empathetic) character, begins as ambivalent but changes into a positive role model to promote positive behaviour change(15). This is a deliberate method to immerse the audience in the action, rather than passively watching or listening (16). AGYW, or at least early adopters, are anticipated to be immersed in the serial, able to classify and identify with the transitional characters and their outcomes. Pathways to behaviour change through MTV Shuga, therefore relate to the extent to which the observer, including early adopters, are immersed and critically engaged with the story. It also depends on a context which is supportive rather than disruptive (see the conceptual framework Figure 1 below) (17).

Figure 1 showing conceptual framework for MTV Shuga impact on HIV prevention on AGYW in uMkhanyakude

A cluster randomised controlled trial of community-viewings of MTV Shuga in Nigeria found that exposure to MTV Shuga significantly improved HIV knowledge and testing in both sexes, the intervention arm showing 35% more likely to test for HIV than the control arm. There was also a 60% reduction in genital chlamydia as a marker of recent sexual risk in women, amongst those exposed to MTV Shuga compared to those who were not (18). There were, however, fewer changes in social norms, particularly around gender-based violence. Further work suggested that the impact was greatest in those who were immersed in the narrative. The importance of immersion (classification of characters and identifying with them and observing outcomes) coupled with critical participation and an enabling context were also found in a

For peer review only - http://bmjopen.bmj.com/site/about/guidelines.xhtml

thematic analysis looking at how storylines in MTV Shuga-DS shaped awareness, knowledge and opinions of sexual health and personal relationships among young people in SA (17).

Whilst the Nigerian and SA studies provide evidence for the efficacy of MTV Shuga impacting on SRHR and HIV-testing behaviours in exposed individuals, there is little evidence of how this will translate into a population-level effect when nationally broadcast and in less controlled environments. In particular, it is not clear how the impact will spill-over to non-viewers and how innovations will diffuse when shown and watched by adolescents and young adults in a real-world scale-up. It is also unclear how such impact will differ according to: setting (rural or urban); differential digital literacy and access to social media (geographically and socioeconomically), and the dose and context of watching (shared viewing with family, friends or individual experience through social media).

We use the opportunity of MTV Shuga being broadcast in the context of an impact evaluation of DREAMS roll out (January 2016-September 2019) in a representative, population-based sample of young people (12) to describe the real-world reach of nationally broadcast MTV Shuga-DS. Further, we explore the hypothesis that exposure to a mass-media serial edu-drama, like MTV Shuga, will improve SRHR outcomes by increasing demand for, and uptake of, existing combination individual and community-based SRH and HIV prevention services for AGYW in four diverse settings including uMkhanyakude, a socioeconomically deprived rural district with an extremely high burden of HIV: 40% antenatal HIV prevalence and an annual HIV incidence of 5% in girls (15-19 years) and 8% in young women (20-24 years) (2, 19) and in three high-prevalence urban districts (HIV prevalence of greater than 10%) of City of Johannesburg, Ekurhuleni, and eThekwini.

Methods

Study design

We employed a longitudinal cohort and cross-sectional surveys of representative samples of AGYW aged 12-24 in four districts of South Africa with a high burden of HIV to measure the reach of MTV Shuga-DS. Data were collected between May 2017 and September 2019.

We used baseline and follow-up data from a nested cohort of 2184 AGYW aged 13-22 years, enrolled in 2017 for the DREAMS impact evaluation. The cohort is nested in a large population-based longitudinal HIV surveillance study, in the uMkhanyakude district of KwaZulu-Natal (20, 21). A random sample of 3013 AGYW was selected from the surveillance

For peer review only - http://bmjopen.bmj.com/site/about/guidelines.xhtml

population, stratified by age (13-17 years and 18-22 years) and geography, and invited to enrol in the nested cohort. Baseline interviews were conducted between May 2017 and February 2018 and follow-up interviews April 2018 and September 2019 in the local language (isiZulu) using a structured quantitative questionnaire programmed in REDCap (12).

The cross-sectional survey was conducted on a household-based representative sample of 4127 AGYW (between the ages 12-24 years) in three high prevalence (City of Johannesburg, Ekurhuleni and eThekwini) districts. Between August 2017 and July 2018, a stratified cluster-based sampling strategy was used to select 18500 AGYW aged 12-24 eligible for a cross-sectional survey of individuals, based on an expected response rate of 80% (22).

The interview included questions on socio-demographics, general health, exposure to DREAMS and to MTV Shuga, sexual relationships, awareness and uptake of DREAMS and DREAMS-like services, migration, and gender norms across the four districts. A Dried Blood Spot (DBS) was taken at baseline and follow-up for Herpes Simplex Virus type 2 (HSV-2) antibody testing in the uMkhanyakude district. For sexual behaviour questions, participants were given a tablet computer to complete a self-interview; the fieldworker was available to provide support as needed.

Study setting and population

The cohort was nested within the Africa Health Research Institute (AHRI) triannual demographic surveillance of a population of approximately 150,000 people who are members of 15,000 geocoded households in an area of 845 km^2 (20). The study area is mostly rural and poor with high levels of youth unemployment (over 85% of those aged 18-24 are unemployed) (2, 19).

The cross-sectional survey was conducted in three districts which were mostly urban with more towns and townships compared to AHRI surveillance area. The three study districts (City of Johannesburg, Ekurhuleni and eThekwini) consist of an estimated 12,073,421 individuals. The eThekwini district in KZN province is among those with the highest HIV prevalence (16.8% HIV prevalence in 2016) in South Africa. Over two-thirds (68%) of eThekwini is considered rural and 32% urban. About 11 963 (3%) AGYW in eThekwini are estimated to be living with HIV. The Gauteng province (GP), whilst geographically the smallest, is the most populous province in South Africa. GP has the fifth highest provincial HIV prevalence in the country with a prevalence of 11.1% among those aged 15 to 49 years old in 2016. The HIV prevalence in the two districts, City of Johannesburg and Ekurhuleni, is 11.1% and 14.3% among 15- to 49-

For peer review only - http://bmjopen.bmj.com/site/about/guidelines.xhtml

year-olds, respectively. Both districts are densely populated and have high levels of industrialisation. The HIV prevalence amongst AGYW (15 to 24 years old) in the City of Johannesburg is 3%, and similarly 3% in Ekurhuleni in 2012.

Variables and Measurement:

Outcomes definitions:

We measured the effect of exposure to MTV Shuga on awareness and uptake of HIV prevention and SRHR outcomes at the follow-up visit. Our outcomes were: 1) self-reported HIV-testing in the past 12 months; 2) awareness of Pre-Exposure Prophylaxis (PrEP) for HIV prevention; 3) condom use at last sex; 4) use of contraception; 5) any new pregnancy since baseline; and 6) any new teenage pregnancy (restricted to those under the age of 20). AGYW were considered to use contraception if they self-reported using pill, injection, Intrauterine Device (IUD), implant, sterilisation or self-reported consistent condom use (using condoms as a contraceptive method and at last sex). Condomless sex was calculated using 2019 data among participants who reported having had sex with the most recent partner in the past 12 months. Recent pregnancy was calculated as any new pregnancy that occurred between baseline and 2019, while teenage pregnancy was calculated as any new pregnancy that occurred between baseline and 2019 among participants aged below 20 years. We also examined the effect of exposure to MTV Shuga on incident HSV-2 infections, among those who were HSV2 negative at baseline.

Exposure definitions:

Exposure to MTV Shuga was defined as ever watched MTV Shuga between 2017 and 2018. The level of exposure was measured based on the content of the series MTV Shuga, defined using 15 questions used to assess knowledge of content of MTV Shuga series. A composite score was developed summing-up the correct responses. The scores ranged between 2 and 14, and the median being 4. The median was used as a cut-off to define level of exposure among those who watched the series. Consequently, the level of exposure was categorized into 3 levels: High (watching a MTV Shuga and being able to correctly respond to 5 or more questions on content); Medium (watching programme and being able to correctly respond to less than 5 questions); and None (not watched any MTV Shuga). This was further categorised into 3 levels: High (watching MTV Shuga and being able to recall the content from MTV Shuga); Medium (watching programme, but unable to recall content); and None (not being aware of and not watched any MTV Shuga).

For peer review only - http://bmjopen.bmj.com/site/about/guidelines.xhtml

Potential confounding variables:

We included socio-demographic and sexual behaviour characteristics of AGYW that were measured at baseline and exposure to HIV prevention. The socio-demographic variables included age (as measured at follow-up), household socio-economic status, education broken down by those who are still in school and those who have completed school, geographic area (rural or peri-urban/urban), and migration in the last 12 months. The socio-economic status (SES) variable was constructed using Principal Component Analysis (PCA) based on ownership of household assets and characteristics such as access to piped water, type of toilet, electricity and type of cooking fuel (23). Further, potential individual-level confounders measured included exposure to DREAMS (defined either as ever been invited to participate in any of the DREAMS activities or ever used any of the DREAMS HIV prevention interventions in the past 12 months or since 2016 and phone ownership at baseline.

Laboratory:

The HerpeSelect® 2 ELISA IgG assay (FOCUS Diagnostics, Cypress, California, USA) for the qualitative detection of human IgG class antibodies to HSV-2 was used on Dried blood Spot (DBS) samples collected on Whatman 903 filter cards. The HerpeSelect ® 2 ELISA IgG assay uses purified type-specific gG-2 antigen immobilized on polystyrene microwells reducing the cross-reactivity issues as seen with viral lysate assays(24). The assay is validated for use with serum samples but was optimised for use with DBS in the AHRI Diagnostic Research Laboratory following comparative testing with plasma samples. During the initial evaluation of the HerpeSelect ® 2 ELISA IgG a select number of plasma samples were also tested by an external accredited pathology laboratory. A 6mm diameter punch of a DBS spot was incubated overnight in 150ul Assay Diluent for no more than 16 hours at 4°C. The assay was performed with 50ul of the eluent in accordance with the manufacturer's instructions. Following a disproportionately high number of positive results based on other studies and our experience we multiplied the mean cut-off calibrator absorbance values by a factor of 1.5 before determining the index value for each sample (25, 26). The HerpeSelect ® 2 ELISA IgG results are reported as positive (index value >1.10), equivocal (index value of ≥ 0.90 but ≤1.10) or negative (index value <0.90). All initial equivocal results were re-tested and those that re-tested equivocal are reported as equivocal. An incident HSV-2 was defined as having been negative at baseline and positive at follow-up. Those who were equivocal at either baseline or follow-up were not considered as a sero-conversions.

For peer review only

Statistical analyses:

We conducted two separate analyses for cohort and cross-sectional data. For the nested cohort, we included only participants who had data available at baseline and follow-up. We used Chi-square tests to compare baseline characteristics between AGYW who did and did not have any exposure to MTV Shuga. We used logistic regression to examine the effect of MTV Shuga on health outcomes, adjusting for exposure to DREAMS and all other potential confounders. Potential effect-modification of MTV Shuga by exposure to DREAMS was examined by fitting an interaction term to fully adjusted model: likelihood ratio tests were used to compare models with and without interaction terms.

We calculated the proportion of AGYW who reported an outcome (condomless-sex, recent pregnant) or tested positive for HSV-2 at 12-month or 24-month follow-up; and estimated associations between MTV Shuga and each outcome using a logistic regression, adjusting for potential confounders (age, household and individual socio-demographic characteristics and sexual behaviour). For HSV-2 incidence, we included participants who tested negative at baseline and had at least 1 follow-up test result. For DREAMS exposure, we included data collected at baseline and 12-month follow-up. For health outcomes (consistent condom use, modern contraception, HIV testing, PrEP awareness), we used data collected at 24-month follow-up; and for HSV-2 and pregnancy incidence we used data collected at 12 and 24-month follow-up.

Propensity score logistic regression adjustment was used to estimate the causal effect of MTV Shuga on health outcomes. A propensity or probability of being exposed to MTV Shuga was measured by fitting a logistic regression with MTV Shuga exposure as an outcome and potential confounders. A logistic regression models adjusting for propensity scores were then used to predict the probability of an outcome for all participants and separately by age group, under two scenario (1) exposed to MTV Shuga and (2) Not exposed to MTV Shuga. The predicted probabilities were then used to calculate the marginal risk difference, prevalence ratio and odds ratio. Confidence intervals were generated by using a bootstrap procedure, repeating the estimation procedure described above in 1000 samples that were drawn with replacement from the complete dataset and calculating 95% confidence intervals from the resulting bootstrap distribution using the 2.5% and 97.5% percentiles. We also used propensity score stratification and probability weighting methods to check the consistency of our findings.

For peer review only - http://bmjopen.bmj.com/site/about/guidelines.xhtml

For cross-sectional survey, we used Chi square test to compare the characteristics of AGYW who did and did not have any exposure to MTV Shuga; and logistic regression models adjusting for potential confounders were used to examine the association between exposure to MTV Shuga and health outcomes. Sampling weights were applied to achieve proportionality between groups of participants in the survey.

All analyses were performed using Stata version 15 (StataCorp LP, College Station, Texas USA).

Reporting

The STROBE reporting guidelines were used to guide synthesis and standardise reporting of our results(27)

Patient and public involvement

The study did not involve patients. AHRI has a Public Engagement Unit which conducts community engagement activities with the local communities as part of study findings dissemination. The Community Advisory Board provided feedback of the study including design before approval from ethics review board. Study findings are being made publicly available to funders, participants and the public through webinars, study reports and open access journal articles.

Results:

Participants

Of 3013 potentially eligible AGYW randomly selected from the surveillance data set, 85.5% of those eligible consented to participate at baseline (Figure 2). Of the 2184 eligible participants that were surveyed at baseline, 2016 (92.3%) had at least one follow-up visit and contributed data to this analysis. From the cross-sectional survey, 4127 (22.6%) eligible participants were surveyed.

Figure 2 Flowchart showing AGYW follow-up from nested cohorts 2017-2019

Awareness and exposure to MTV Shuga

MTV Shuga exposure at baseline was limited, with a total of only 308 (14.1%) respondents reported watching at least one episode. In the cross-sectional analysis of the three districts, a total of 1477 (35.8%) reported watching any MTV Shuga. In the nested cohort 121 (5.5%)

For peer review only

recalled any storyline. Similarly in the cross-sectional snapshot 276 (6.7%) recalled the storyline (high exposure).

Social demographic characteristics of adolescents and young people by exposure to MTV Shuga (Table 1&2)

Table 1 summarises the profiles of the nested cohort (n=2184) and cross-sectional surveys (n=4127) AGYW comparing those exposed to MTV Shuga with those not exposed. In summary those who had seen any MTV Shuga were more likely to be from households in the highest socioeconomic tertile (p<0.001) and more urbanised areas (p<0.001). They were also more likely to have also received DREAMS (p=0.015) (Table 1). In the cross-sectional surveys (n=4127), MTV Shuga exposure was associated with older age (p<0.001), tertiary education (p <0.001) and never having sex (p< 0.001) Table 1.

Table 1. Baseline socio-demographic characteristics of adolescent girls and young women (13-22) by exposure to MTV Shuga in the nested cohort (n=2184)

	Overall		Exposed		Not exposed		
	n/N	%	n/N	%	n/N	%	p-value
**Age group (4 cats), 2017**							
13-14	460/2184	21.1	72/308	23.4	388/1876	20.7	
15-17	688/2184	31.5	107/308	34.7	581/1876	31	
18-19	475/2184	21.7	60/308	19.5	415/1876	22.1	
20-22	561/2184	25.7	69/308	22.4	492/1876	26.2	0.216
**Currently in school**							
No	540/2184	24.7	67/308	21.8	473/1876	25.2	
Yes	1644/2184	75.3	241/308	78.2	1403/1876	74.8	0.192
**Socio-economic status, 2018**							
Low	255/2118	12	22/303	7.3	233/1815	12.8	
Middle	920/2118	43.4	110/303	36.3	810/1815	44.6	
High	943/2118	44.5	171/303	56.4	772/1815	42.5	<0.001
**Urban or rural**							
Rural	1388/2165	64.1	165/305	54.1	1223/1860	65.8	
Peri-urban/urban	777/2165	35.9	140/305	45.9	637/1860	34.2	<0.001
**Invited or received DREAMS, 2017/18**	1101/2184	50.4	175/308	56.8	926/1876	49.4	0.015
**Away from home in the last 12 months**	314/1853	17.0	45/228	15.6	269/1565	17.2	0.516
**Baseline socio-demographic characteristics of adolescent girls and young women (12-24) by exposure to MTV Shuga in the cross-sectional survey (n=4127)**							
	Overall		Exposed		Not exposed		

For peer review only - http://bmjopen.bmj.com/site/about/guidelines.xhtml

	n/N	%	n/N	%	n/N	%	p-value
**Age group, (N=4127)**							
12-14	958/4127	23.2	307/1477	20.1	651/2650	24.6	
15-19	1628/4127	39.5	599/1477	40.6	1029/2650	38.8	
20-24	1541/4127	37.3	571/1477	38.7	970/2650	36.6	0.022
**District**							
City of Johannesburg	1146/4127	27.8	476/1477	32.2	670/2650	25.3	
Ekurhuleni	1635/4127	39.6	521/1477	35.3	1114/2650	42.0	
eThekwini	1342/4127	32.5	480/1477	32.5	862/2650	32.5	<0.001
**Highest Education (N=4108)**							
No schooling	175/4108	4.3	99/1465	6.8	76/2646	2.9	
Grade R to 7	502/4108	12.2	142/1465	9.7	360/2646	13.6	
Grade 8 to 12	2978/4108	72.5	1022/1465	69.8	1956/2646	74.0	
Tertiary studies (complete/incomplete)	453/4108	11.0	202/1465	13.8	251/2646	9.5	<0.001
**Ever had sex with a boy/man (n=4108)**	1860/4108	45.3	621/1469	42.3	1239/2639	47.0	0.004
**Away from home in the last 12 months (n=4121)**	183/4121	4.4	66/1474	4.5	117/2647	4.4	0.932

After adjustment for confounders in nested cohorts (Table 2), AGYW from wealthier households (aOR=2.04 95%CI 1.27-3.30), peri-urban or urban areas (aOR=1.54 95%CI 1.19-1.98) and those invited to DREAMS (aOR=1.48 95%CI 1.14-1.92) were more likely to be exposed to MTV Shuga than those from poor households, from rural areas and those not invited to DREAMS respectively. Similarly, after adjustment in the cross-sectional surveys (Table 2), AGYW with higher education were more likely to be exposed to MTV Shuga (aOR=2.58 95%CI 1.81-3.69) than those with less and those who ever had sex (aOR=0.68 95%CI 0.57-0.82) were less likely to be exposed to MTV Shuga.

Table 2 Factors associated with exposure to MTV Shuga in the nested cohort of AGYW aged 13-22 (n=2184)

	Unadjusted		Adjusted - All		
	OR	95%CI	OR	95%CI	P-value
**Age group, 2017**					
13-14	1		1		
15-17	0.99	0.72 -1.37	0.93	0.66 -1.29	
18-19	0.78	0.54 -1.13	0.77	0.51 -1.14	
20-22	0.76	0.53 -1.08	0.78	0.50 -1.21	0.555
**Currently in school**					
No	1		1		
Yes	1.21	0.91 -1.62	1.06	0.78 -1.43	0.726

For peer review only - http://bmjopen.bmj.com/site/about/guidelines.xhtml

**Socio-economic status, 2018**					
Low	1		1		
Middle	1.44	0.89 -2.33	1.27	0.78 -2.07	
High	2.35	1.47 -3.74	2.04	1.27 -3.30	<0.001
**Site**					
Rural	1		1		
Peri-urban/ urban	1.63	1.28 -2.08	1.54	1.19 -1.98	0.001
**Invited or received DREAMS, 2017/18**					
No	1		1		
Yes	1.35	1.06 -1.72	1.48	1.14 -1.92	0.003

Factors associated with exposure to MTV Shuga in the cross-sectional analysis of AGYW aged 12-24 (n=4127)

	**Unadjusted**		**Adjusted - all**		
	**OR**	**95% CI**	**OR**	**95% CI**	**P-value**
**District**					
City of Johannesburg	1		1		
Ekurhuleni	0.69	0.54 - 0.89	0.66	0.52- 0.84	0.001
eThekwini	0.79	0.60 - 1.04	0.78	0.59- 1.04	0.087
**Age group**					
12-14	1		1		
15-19	1.27	1.06 - 1.53	1.18	0.95- 1.46	0.126
20-24	1.22	1.01 - 1.48	1.16	0.90- 1.49	0.256
**Highest education level**					
Grade R to 7	1		1		
No schooling	2.88	1.81 - 4.58	3.29	2.01 - 5.38	<0.001
Grade 8 to 12	1.38	1.09 - 1.75	1.42	1.08 - 1.86	0.011
Complete or incomplete tertiary	2.29	1.70 - 3.09	2.58	1.81 - 3.69	<0.001
**Ever had sex with a boy/man**					
No	1		1		
Yes	0.82	0.71 - 0.95	0.68	0.57- 0.82	<0.001
**Away from home in last 12 months**					
No	1		1		
Yes	1.15	0.80 - 1.64	1.21	0.83- 1.77	0.309

For peer review only - http://bmjopen.bmj.com/site/about/guidelines.xhtml

Relationship between MTV Shuga exposure and HIV and SRHR outcomes (Table 3&4)

In the nested cohorts by 2019, overall 63.3% of those aged 14-23 knew their HIV status, 13.4% were consistently using contraception, 20.0% were using condoms consistently, and about a third were aware of PrEP, 5% had a pregnancy and 15% acquired HSV2. There were higher proportions of contraception use, condom use and PrEP awareness among those exposed to MTV Shuga (Table 3). For survey sites (Table 3) overall, 85.0% knew their HIV status, over a fifth 22.6% were using contraception and about half using condoms 48.4%. About a tenth (7.5%) were aware of PrEP, with higher proportions of these being among those exposed to MTV Shuga.

Table 3: HIV and SRHR outcomes by exposure to MTV Shuga nested cohort 13-22 year olds (n= 2167)

	Overall		Exposed (n=308)		Not exposed (n=1878)		P-value
	n/N	%	n/N	%	n/N	%	
Knowledge of HIV status, 2019	1083/1712	63.3	175/283	61.8	908/1429	63.5	0.587
Modern contraception, 2019	221/1651	13.4	56/271	20.7	165/1380	12	<0.001
Consistent condom use, 2019	168/838	20	41/141	29.1	127/697	18.2	0.003
Aware of PrEP, 2019	523/1712	30.5	124/283	43.8	399/1429	27.9	0.302
Pregnant in 2018/19	124/2184	5.7	20/308	6.5	104/1876	5.5	0.504
Teenage pregnancy	72/1395	5.16	55/1187	4.63	17/208	8.17	0.033
HSV-2 2018/19	241/1562	15.4	35/237	14.8	206/1325	15.5	0.760
**HIV and SRHR outcomes by exposure to MTV Shuga weighted for sampling cross sectional survey 12-24 year olds (n=4127)**							
	**Overall**		**Exposed**		**Not exposed**		
	**n/N**	**%**	**n/N**	**%**	**n/N**	**%**	**p-value**
PrEP awareness (N=4127)	310/4127	7.5	148/1477	10.0	162/2650	6.1	<0.001
HIV test (self-report) (N=2529)	2156/2529	85.3	656/797	82.3	1500/1732	86.6	0.005
Condom use at last sex (N=1898)‡	918/1898	48.4	320/640	50.0	598/1258	47.5	0.310
Contraception use (N=4127)	934/4127	22.6	302/1477	20.5	632/2650	23.9	0.012

‡Restricted to those who ever had sex with man

At follow up, incident HSV-2 and teenage pregnancy were high, HSV-2 incidence was 15.26 and teenage pregnancy incidence was 9.86 per 100 person-years, respectively (Table 4).

For peer review only - http://bmjopen.bmj.com/site/about/guidelines.xhtml

Table 4: Rate of SRHR outcome by exposure to MTV Shuga (nested cohort 13-22 year olds n=2167)

**HSV-2 incidence rate**					
	person-time	n with HSV-2	Rate/ 100 py	[95% Conf.	Interval]
MTV Shuga Not exposed	1303.0	206	15.81	13.79	18.12
MTV Shuga Exposed	276.3	35	12.67	9.09	17.64
Total	1579.3	241	15.26	13.45	17.31
**Pregnancy incidence rates among all AGYW**					
	person-time	n pregnant	Rate/100 py	[95% Conf.	Interval]
MTV Shuga Not exposed	1068.7	104	9.73	8.03	11.79
MTV Shuga Exposed	188.5	20	10.61	6.84	16.44
Total	1257.2	124	9.86	8.27	11.76
**Pregnancy incidence rates among girls below 19 years**					
	person-time	n pregnant	Rate/100 py	[95% Conf.	Interval]
MTV Shuga Not exposed	668.7	55	8.22	6.31	10.71
MTV Shuga Exposed	123.9	17	13.72	8.53	22.07
Total	792.6	72	9.08	7.21	11.44

MTV Shuga and HIV prevention and SRHR awareness and uptake

In the nested cohort after adjusting for age, education, SES, area and DREAMS, MTV Shuga exposure in the AGYW cohort was associated with significantly greater awareness of PrEP (aOR=2.06, 95%CI: 1.57-2.70), contraception uptake (aOR=2.08, 95%CI: 1.45-2.98), and consistent condom use (aOR=1.84, 95%CI: 1.24-2.93). Watching MTV-Shuga was not associated with HIV testing (aOR=1.02,95%CI: 0.77-1.21) (Figure 3 and Supplementary Tables S1-S4). There was no effect modification by DREAMS exposure.

Figure 3 Forest plots showing the association between MTV Shuga exposure and HIV prevention and SRHR awareness and uptake in the nested cohorts.

Similarly, in the cross-sectional analysis, after adjusting for age, education, district, migration and sexual history, exposure to MTV-Shuga watching was associated with greater awareness of PrEP (aOR=1.70, 95%CI 1.20 - 2.43). However, there was no association with contraception, lower self-reported HIV testing, or condom use as shown in Figure 4 below (Supplementary Table 5).

For peer review only - http://bmjopen.bmj.com/site/about/guidelines.xhtml

Figure 4 Forest plots showing association between MTV Shuga exposure and HIV prevention and SRHR awareness and uptake in the cross-sectional surveys

Causal effect of MTV Shuga on health outcomes – HSV2

The causal analysis similarly finds no effect of MTV Shuga on HSV2, with a risk difference of 1.10 95% CI (-2.82- 5.38)%. Findings in the younger age group (aged 13-17) and the older age group (18-22) was similar (Supplementary Table 6).

Discussion

In our study of the population-level effect of a national broadcast of a TV-based edu-drama on HIV prevention and SRHR, exposure to MTV Shuga was associated with higher awareness of a novel HIV prevention intervention (PrEP). However, despite a very high incidence of HSV-2 and teenage pregnancy, MTV Shuga exposure was not significantly associated with safer sexual behaviour, uptake of contraception and HIV-testing or prevention of teenage pregnancy. Notably though, the size of the relationship and direction of effect between exposure to MTV Shuga, condom use and markers of unprotected sex (HSV-2 and pregnancy) was consistent with a possible relationship. These findings may be partly explained by our finding that less than one in 12 of the target age group had any exposure to MTV Shuga and only half of these had high exposure (defined as watching the MTV Shuga South African series and being able to recall the content), suggesting that one of the limiting factors for the effective use of TV-based edu-drama maybe the dose that young people are exposed to, particularly in rural and resource-constrained settings most affected by HIV.

Our inability to find a measurable population effect of mass-media edu-drama behaviour change campaign compared to the trial findings of the RCT is disappointing (18). However, firstly the direction and size of the relationships suggest that we may have been able to see an effect if the proportion exposed had been greater than 7%. Secondly, AGYW in urban settings were more likely to have been exposed to MTV Shuga and they are also more likely to be living in small towns and townships. Data from our settings suggest that young people in small towns and townships are more vulnerable to HIV (28) and sexual risk (29) and therefore it is possible that a real effect of MTV Shuga on this group was masked by their greater risk for the outcome. Due to the low numbers with high levels of exposure to MTV Shuga we did not have the power to explore this hypothesis by looking at effect modification by geography or socioeconomic status.

For peer review only - http://bmjopen.bmj.com/site/about/guidelines.xhtml

The finding of a differential association of MTV Shuga exposure with awareness of PrEP, compared to uptake of HIV and contraception, suggests that whilst educational mass entertainment may be able to increase awareness and possibly demand for a service, it does not impact on accessibility of the service: i.e. it impacts the first two steps of the prevention cascade and not the final step(30). Well described barriers to uptake of HIV-testing and contraception in this area are internalized and externalized stigma, fear of judgement from health care workers and the social costs of accessing care in busy primary health care settings(31-33). Behaviour change intervention including mass communication campaigns can be constrained or facilitated by the context in which people live (17, 34). To optimise MTV Shuga's effect there may need to be parallel innovations in SRHR and HIV service delivery that makes the services easier to access. We aim to test this hypothesis by providing community-based delivery of HIV and SRHR services in the context of the MTV Shuga.

The behaviour change theory that underpins edu-drama as a vehicle for mass behaviour change communication (16) explicitly suggests that the audience, and especially the early adopters, need to be actively watching, rather than passively watching or listening (17, 18). TV watching in rural homesteads can be in the context of large, often grandparent-led households and competing chores and priorities. This coupled with the relatively late timing of the shows may explain why so few girls and young women were sufficiently engaged or immersed to be able to recall characters or story lines. Moreover, the timing of this analysis may have allowed insufficient time for early adopters to convey the message of the show.

Limitations

The limitations of our study are that a meaningful exposure to MTV Shuga was low and so whilst the size of the relationship and direction of effect between exposure to MTV Shuga, condom use, and markers of unprotected sex (HSV-2 and pregnancy) suggested a possible effect, but we did not have the power to show a significant relationship between exposure to MTV Shuga and SRHR outcomes. We also do not have the power to see a difference by dose and immersion. Furthermore, as this is an observational study, we cannot exclude the possibility that those who are exposed to MTV Shuga are systematically different in ways that impact on the outcome of interest, for example more exposed to social media and sexual health promotion and innovative technologies to support sexual health than those who were not.

For peer review only - http://bmjopen.bmj.com/site/about/guidelines.xhtml

Conclusions and implications for the future

- Meaningful exposure to MTV Shuga was low across rural and urban settings in South Africa and so additional efforts need to be made to reach young people and increase their immersion in promising edu-drama if it is to have the desired effect, especially in rural and deprived settings.
- MTV Shuga was an effective vehicle to raise awareness and promote newer HIV prevention technologies such as HIV PrEP.
- There was some suggestion that MTV Shuga improved uptake of some HIV prevention and sexual health technologies (contraception and condoms)
- There was less evidence from this observational study that it improved SRHR and HIV outcomes

We highlight the importance of evaluating the real-world scale up of promising interventions to understand both the reach and population effect as well as inform interventions to increase impact and equity.

Efforts to increase exposure, which have been rolled out as part of MTV Shuga in SA, such as social media, school-based or community-based MTV Shuga film clubs will need to be evaluated, both to understand whether or not they increase exposure and coverage and improve SRH and HIV outcomes. However, to have a significant impact on the HIV and SRH prevention and treatment cascades, demand generation in AGYW needs to be delivered in parallel with accessible service delivery models that support adherence and retention (30).

Contributorship

M.S., N.C., J.D., T.Z. N.K, G.H. and C.C. developed the study tools and performed the research. M.S., J.S. D.P., K.B., I.B., and S.F. designed the research study. T.S. and S.D. conducted the laboratory analysis. N.M. supported by K.B. conducted the statistical analysis and G.C. M.S wrote the first and final draft of the paper with input from N.C., N.M., G.H., J.S., I.B., G.C., K.B., C.C., T.S., T.Z, D.P and N.K. All the authors critically reviewed the manuscript. All authors have approved the final draft of the paper.

Competing interests

All authors declare they have no conflict of interest

For peer review only - http://bmjopen.bmj.com/site/about/guidelines.xhtml

Funding

The impact evaluation of DREAMS and MTV Shuga is funded by the Bill and Melinda Gates Foundation (OPP1136774 and OPP1171600, http://www.gatesfoundation.org). Foundation staff advised the study team, but did not substantively affect the study design, instruments, interpretation of data, or decision to publish. The research leading to these results has received funding from the People Programme (Marie Curie Actions) of the European Union's seventh Framework Programme FP7/2007-2013 under REA grant agreement n° 612216. MS National Institutes of Health 5R01MH114560-03 funding acknowledgement. This research was funded in whole, or in part, by the Africa Health Research Institute through the Wellcome [Strategic Core award: 201433/Z/16/A]. For the purpose of open access, the author has applied a CC BY public copyright licence to any Author Accepted Manuscript version arising from this submission. N.C is supported by a training fellowship from the National Institute for Health Research (NIHR) (using the UK's Official Development Assistance (ODA) Funding) and Wellcome [grant reference number 224309/Z/21/Z] under the NIHR-Wellcome Partnership for Global Health Research. The views expressed are those of the authors and not necessarily those of Wellcome, the NIHR or the Department of Health and Social Care. GH is supported by a fellowship from the Royal Society and the Wellcome Trust [Grant number 210479/Z/18/Z]. MS work is also supported by a BMGF 3ie grant at AHRI. This research was funded in whole, or in part, by the Wellcome Trust [Grant number 210479/Z/18/Z]. For the purpose of open access, the author has applied a CC BY public copyright licence to any Author Accepted Manuscript version arising from this submission.

Data sharing statement

Data are available on reasonable request.

Ethics statement

The DREAMS Partnership impact evaluation protocol was reviewed and approved by the University of KwaZulu-Natal (UKZN) Biomedical Research Ethics Committee (BREC) (BFC 339/16), the London School of Hygiene & Tropical Medicine Research Ethics Committee (Ref 11835) and the AHRI Somkhele Community Advisory Board, the Associate Director of Science of the Center for Global Health (CGH) at the United States Centers for Disease Control and Prevention (CDC) in Atlanta and the Department of Health, Province of KZN and Gauteng. Approval for the demographic surveillance and data collection in the clinics was granted by UKZN BREC. Participation in the study was voluntary. All participants provided separate informed consent to take part in data collection through the questionnaires and the HSV2 sero-

For peer review only

survey. Consent for follow-up interviews was provided separately. For participants aged <18 years, written parental consent and participant assent were provided. All data were anonymised for analysis.

Acknowledgements

The authors are grateful to the communities of the Hlabisa sub-district, Ekurhuleni district, Thekwini district and City of Johannesburg who contributed their data to this study and to all the staff at Africa Health Research Institute and EpiCentre who collected the data. We acknowledge Eskindir Shambullo for his input in this manuscript.

References

1. HSRC. The fifth South African National HIV prevalence, incidence, behaviour and communication survey [Report]. http://www.hsrc.ac.za/uploads/pageContent/9234/SABSSMV_Impact_Assessment_Summary_ZA_ADS_cleared_PDFA4.pdf: South African Health Services Research Council 2017 [updated 01/11/2018.
2. Chimbindi N, Mthiyane N, Birdthistle I, Floyd S, McGrath N, Pillay D, et al. Persistently high incidence of HIV and poor service uptake in adolescent girls and young women in rural KwaZulu-Natal, South Africa prior to DREAMS. PLoS One. 2018;13(10):e0203193.
3. Vandormael A, Akullian A, Siedner M, de Oliveira T, Bärnighausen T, Tanser F. Declines in HIV incidence among men and women in a South African population-based cohort. Nature Communications. 2019;10(1):5482.
4. Birdthistle I, Kwaro D, Shahmanesh M, Baisley K, Khagayi S, Chimbindi N, et al. Evaluating the impact of DREAMS on HIV incidence among adolescent girls and young women: A population-based cohort study in Kenya and South Africa. PLoS medicine. 2021;18(10):e1003837.
5. Department of Health RoSA. National Campaign for Young Women and Adolescent Girls. . 2016.
6. Saul J, Bachman G, Allen S, Toiv NF, Cooney C, Beamon TA. The DREAMS core package of interventions: A comprehensive approach to preventing HIV among adolescent girls and young women. PLOS ONE. 2018;13(12):e0208167.
7. PEPFAR. Preventing HIV in Adolescent Girls and Young Women: Guidance for PEPFAR Country Teams on the DREAMS Partnership 2015.
8. PEPFAR. South Africa DREAMS overview https://www.pepfar.gov/documents/organization/253958.pdf: PEPFAR; 2017 [

For peer review only - http://bmjopen.bmj.com/site/about/guidelines.xhtml

9. Chang L, Serwadda D, Quinn T, Wawer M, Gray R, Reynolds S. Combination implementation for HIV prevention: moving from clinical trial evidence to population-level effects The Lancet Infectious Diseases 2013;13(1):65-76.
10. Iwuji CC, Orne-Gliemann J, Larmarange J, Balestre E, Thiebaut R, Tanser F, et al. Universal test and treat and the HIV epidemic in rural South Africa: a phase 4, open-label, community cluster randomised trial. Lancet HIV. 2018;5(3):e116-e25.
11. Iwuji CC, Orne-Gliemann J, Tanser F, Boyer S, Lessells RJ, Lert F, et al. Evaluation of the impact of immediate versus WHO recommendations-guided antiretroviral therapy initiation on HIV incidence: the ANRS 12249 TasP (Treatment as Prevention) trial in Hlabisa sub-district, KwaZulu-Natal, South Africa: study protocol for a cluster randomised controlled trial. Trials. 2013;14:230.
12. Birdthistle I, Schaffnit SB, Kwaro D, Shahmanesh M, Ziraba A, Kabiru CW, et al. Evaluating the impact of the DREAMS partnership to reduce HIV incidence among adolescent girls and young women in four settings: a study protocol. BMC Public Health. 2018;18(1):912.
13. Shen F, Han J. Effectiveness of entertainment education in communicating health information: a systematic review. Asian Journal of Communication. 2014;24(6):605-16.
14. Noar SM, Palmgreen P, Chabot M, Dobransky N, Zimmerman RS. A 10-year systematic review of HIV/AIDS mass communication campaigns: have we made progress? Journal of Health Communication. 2009;14(1):15-42.
15. Kyegombe N, Zuma T, Hlongwane S, Nhlenyama M, Chimbindi N, Birdthistle I, et al. A qualitative exploration of the salience of MTV-Shuga, an edutainment programme, and adolescents' engagement with sexual and reproductive health information in rural KwaZulu-Natal, South Africa. Sexual and Reproductive Health Matters. 2022;30(1):2083809.
16. Barker K. Sex Soap and Social change- The Sabedo methadology https://www.populationmedia.org/product/sabido-theory/; http://www.comminit.com/content/sabido-methodology [
17. Baker V, Birdthistle I, Seeley J, Arnold G, Piot S, Hajiyiannis H. How can a TV drama about Love, Sex and Growing up Increase Knowledge and Shape the Sexual Health Behaviours of Young People? . AIDS Impact; Cape Town, South Africa2017.
18. MTV Shuga Soap Opera Turns Edutainment into a Tool to Fight HIV and Gender-Based Violence http://www.worldbank.org/en/news/feature/2017/02/23/mtv-shuga-soap-opera-turns-edutainment-into-a-tool-to-fight-hiv-and-gender-based-violence2017 [

For peer review only - http://bmjopen.bmj.com/site/about/guidelines.xhtml

19. National Department of Health. The National Antenatal Sentinel HIV prevalence survey, South Africa, http://www.health.gov.za/index.php/shortcodes/2015-03-29-10-42-47/2015-04-30-08-18-10/2015-04-30-08-21-56?download=2584:2015-national-antenatal-hiv-prevalence-survey-final-23oct17, 2013 [

20. Herbst K, Law M, Geldsetzer P, Tanser F, Harling G, Barnighausen T. Innovations in health and demographic surveillance systems to establish the causal impacts of HIV policies. Current Opinion on HIV AIDS. 2015;10(6):483-94.

21. Gareta D, Baisley K, Mngomezulu T, Smit T, Khoza T, Nxumalo S, et al. Cohort Profile Update: Africa Centre Demographic Information System (ACDIS) and population-based HIV survey. Int J Epidemiol. 2021;50(1):33-4.

22. George G, Cawood C, Puren A, Khanyile D, Gerritsen A, Govender K, et al. Evaluating DREAMS HIV prevention interventions targeting adolescent girls and young women in high HIV prevalence districts in South Africa: protocol for a cross-sectional study. BMC Women's Health. 2020;20(1):7.

23. Filmer D, Pritchett LH. Estimating Wealth Effects Without Expenditure Data—Or Tears: An Application To Educational Enrollments In States Of India*. Demography. 2001;38:115-32.

24. Arvin A, Prober C. Herpes Simplex Viruses. In: Murray P, Baron E, Pfaller M, Tenover F, Yolkenet R, editors. Manual of Clinical Microbiology 6th Ed Washington D.C.: ASM; 1995. p. 876-83.

25. Delany-Moretlwe S, Jentsch U, Weiss H, Moyes J, Ashley-Morrow R, Stevens W, et al. Comparison of Focus HerpeSelect ® and Kalon TM HSV-2 gG2 ELISA serological assays to detect herpes simplex virus type 2 (HSV-2) antibodies in a South African population. . Sexually Transmitted Infections,. 2009;86(1):46-50.

26. Ashley-Morrow R, Nolkamper J, Robinson N, Bishop N, Smith J. Performance of Focus ELISA test for herpes simplex virus type 1 (HSV-1) and HSV-2 antibodies among women in ten diverse geographical locations. . Clinical Microbiology and Infection 2004;10(6):530-6.

27. STROBE Statement. STROBE Checklists 2007 [Available from: http://www.strobe-statement.org/index.php?id=available-checklists.

28. Cuadros DF, Li J, Branscum AJ, Akullian A, Jia P, Mziray EN, et al. Mapping the spatial variability of HIV infection in Sub-Saharan Africa: Effective information for localized HIV prevention and control. Scientific Reports. 2017;7(1):9093.

29. Ngwenya N, Gumede D, Shahmanesh M, McGrath N, Grant A, Seeley J. Community perceptions of the socio-economic structural context influencing HIV and TB risk, prevention and treatment in a high prevalence area in the era of antiretroviral therapy. African Journal of AIDS Research. 2018;17(1):72-81.

For peer review only - http://bmjopen.bmj.com/site/about/guidelines.xhtml

30. Hargreaves JR, Delany-Moretlwe S, Hallett TB, Johnson S, Kapiga S, Bhattacharjee P, et al. The HIV prevention cascade: integrating theories of epidemiological, behavioural, and social science into programme design and monitoring. Lancet HIV. 2016;3(7):e318-22.

31. Hargreaves JR, Krishnaratne S, Mathema H, Lilleston PS, Sievwright K, Mandla N, et al. Individual and community-level risk factors for HIV stigma in 21 Zambian and South African communities: analysis of data from the HPTN071 (PopART) study. AIDS. 2018;32(6):783-93.

32. Chimbindi N, Zuma T, Nakasone S, Birdthistle I, Mthiyane N, Dreyer J, et al. Platforms, Processes and Perceptions of PrEP Delivery Through DREAMS in a Rural Setting in KwaZulu-Natal, South Africa. . Research for Prevention 2018 (HIVR4P 2018) Madrid, Spain 2018.

33. Adeagbo O, Herbst C, Dlamini N, Mhlongo T, Luthuli M, Xulu S, et al. “If she tests negative, it means I am also negative”: Men’s construction of HIV testing in KwaZulu-Natal, South Africa. . HIV Research for Prevention (HIVR4P) 23 October 2018.; Madrid, Spain **2018**

34. Michie S, van Stralen MM, West R. The behaviour change wheel: A new method for characterising and designing behaviour change interventions. Implementation Science. 2011;6(42).

For peer review only

Figure legends

Figure 1 showing conceptual framework for MTV Shuga impact on HIV prevention on AGYW in uMkhanyakude

Figure 2 Flowchart showing AGYW follow-up from nested cohorts 2017-2019

Figure 3 Forest plots showing the association between MTV Shuga exposure and HIV prevention and SRHR awareness and uptake in the nested cohorts.

Figure 4 Forest plots showing association between MTV Shuga exposure and HIV prevention and SRHR awareness and uptake in the cross-sectional surveys

For peer review only

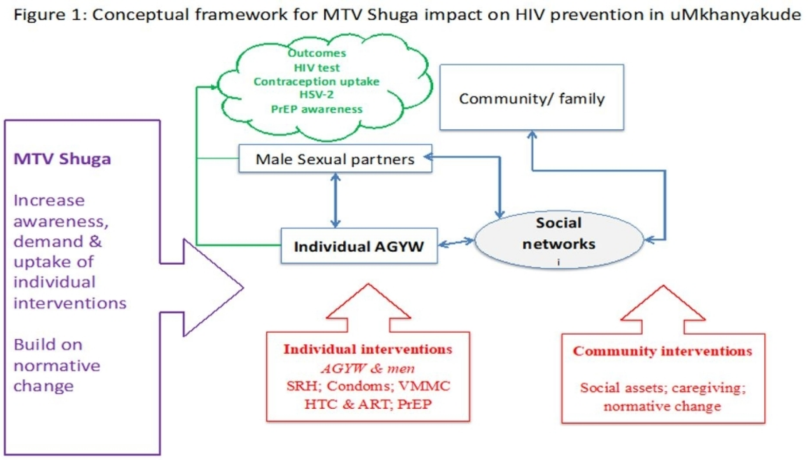

Figure 1 showing conceptual framework for MTV Shuga impact on HIV prevention on AGYW in uMkhanyakude

98x51mm (400 x 400 DPI)

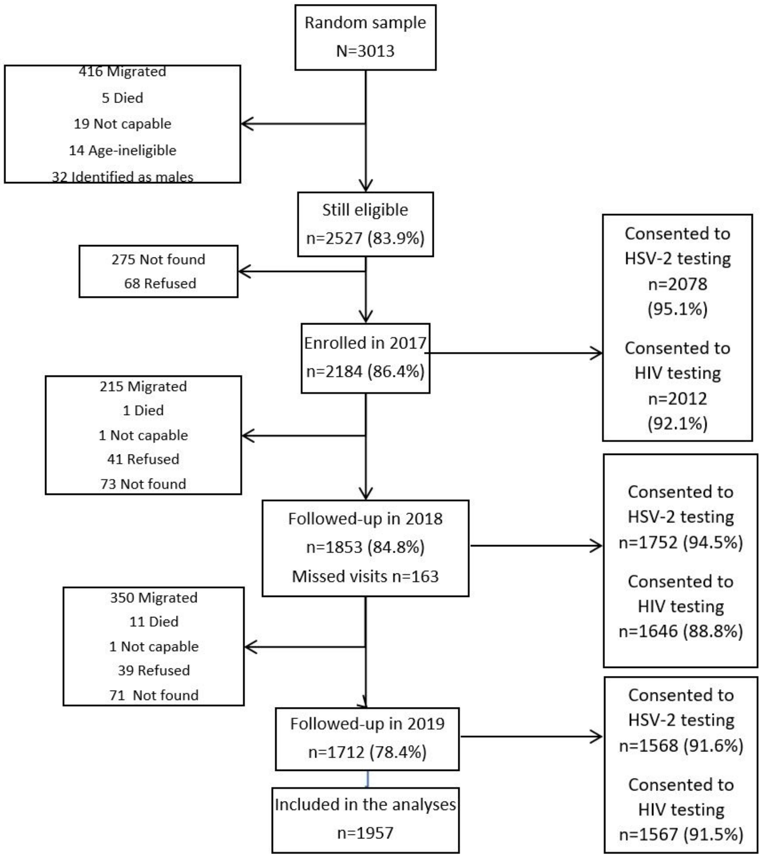

Figure 2 Flowchart showing AGYW follow-up from nested cohorts 2017-2019

71x64mm (400 x 400 DPI)

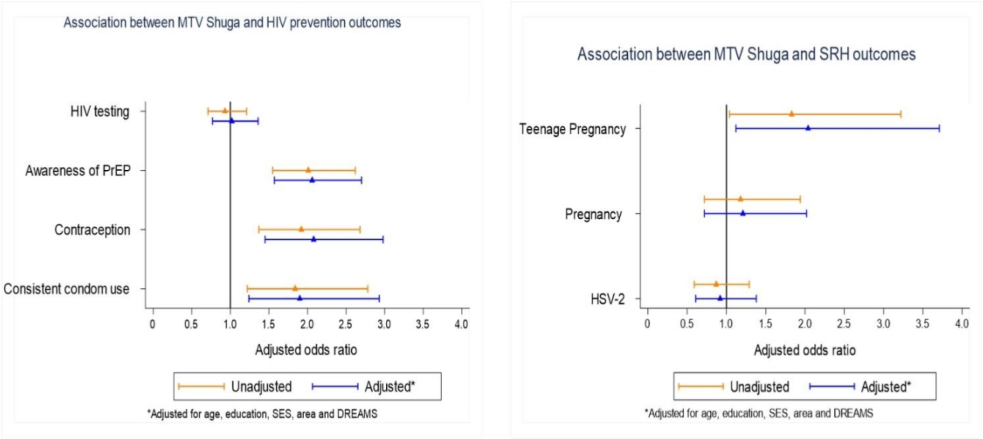

Figure 3 Forest plots showing the association between MTV Shuga exposure and HIV prevention and SRHR awareness and uptake in the nested cohorts.

95x46mm (400 x 400 DPI)

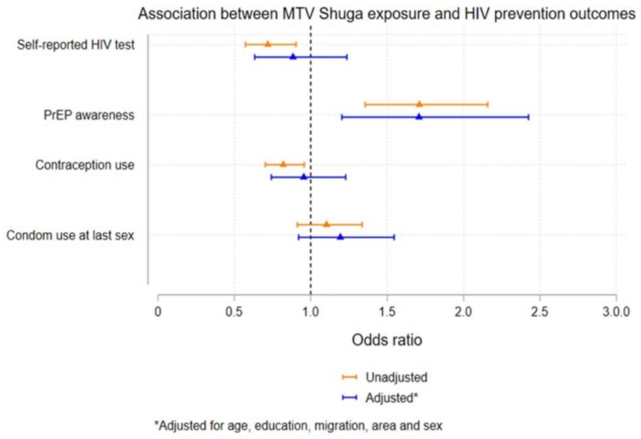

Figure 4 Forest plots showing association between MTV Shuga exposure and HIV prevention and SRHR awareness and uptake in the cross-sectional surveys

101x54mm (400 x 400 DPI)

Supplementary Table 1 Exposure to MTV Shuga and PrEP awareness in the nested cohort of AGYW aged 13-22 (n=2184)

	**Unadjusted**		**Shuga adjusted**			**Adjusted-All**		
	**OR**	**95%CI**	**OR**	**95%CI**	**p-value**	**OR**	**95%CI**	**p-value**
**Ever watched MTV Shuga, 2018/19**								
No	1					1		
Yes	2.01	1.55 -2.62				2.06	1.57 -2.70	<0.001
**Age**	1.12	1.08-1.16	1.12	1.08-1.17	<0.001	1.11	1.06-1.17	<0.001
**Currently in school**								
No	1		1			1		
Yes	0.6	0.47 -0.76	0.59	0.46 -0.75	<0.001	0.89	0.65 -1.23	0.488
**Socio-economic status, 2018**								
Low	1		1			1		
Middle	0.99	0.71 -1.39	0.96	0.68 -1.35		1.01	0.71 -1.44	
High	1.13	0.81 -1.58	1.05	0.75 -1.47	0.744	1.11	0.78 -1.58	0.699
**Urban or rural**								
Rural	1		1			1		
Peri-urban/urban	0.98	0.79 -1.22	0.93	0.75 -1.16	0.525	0.92	0.73 -1.15	0.459
**Invited or received DREAMS, 2017/18**								
No	1		1			1		
Yes	0.89	0.73 -1.10	0.88	0.71 -1.08	0.212	0.99	0.79 -1.23	0.897

For peer review only

Supplementary Table 2 Exposure to MTV Shuga and contraception uptake in the nested cohort of AGYW aged 13-22 (n=2184)

	Unadjusted		Shuga adjusted			Adjusted-All		
	OR	95%CI	OR	95%CI	p-value	OR	95%CI	p-value
**Ever watched MTV Shuga, 2018/19**								
No	1					1		
Yes	1.92	1.37 -2.68				2.08	1.45-2.98	<0.001
**Age**	1.26	1.19-1.33	1.27	1.20-1.34	<0.001	1.32	1.24-1.42	<0.001
**Currently in school**								
No	1		1			1		
Yes	0.6	0.44 -0.83	0.59	0.43-0.82	0.001	1.7	1.13-2.55	0.01
**Socio-economic status, 2018**								
Low	1		1			1		
Middle	0.79	0.51 -1.22	0.76	0.49-1.19		0.8	0.51-1.28	
High	0.86	0.55 -1.34	0.8	0.51-1.24	0.489	0.83	0.52-1.32	0.646
**Urban or rural**								
Rural	1		1			1		
Peri-urban/urban	0.91	0.68 -1.24	0.86	0.64-1.17	0.345	0.89	0.64-1.24	0.49
**Invited or received DREAMS, 2017/18**								
No	1		1			1		
Yes	0.84	0.63 -1.11	0.83	0.62-1.10	0.191	1.04	0.76-1.42	0.813

For peer review only

Supplementary Table 3 Exposure to MTV Shuga and consistent condom use in the nested cohort of AGYW aged 13-22 (n=2184)

	Unadjusted		Shuga adjusted			Adjusted-All		
	**OR**	**95%CI**	**OR**	**95%CI**	**p-value**	**OR**	**95%CI**	**p-value**
**Ever watched MTV Shuga, 2018/19**								
No	1					1		
Yes	1.84	1.22-2.78				1.9	1.24-2.93	0.003
**Age**	0.94	0.86-1.01	0.94	0.87-1.02	0.157	1	0.91-1.10	0.984
**Currently in school**								
No	1		1			1		
Yes	1.76	1.22-2.53	1.73	1.20-2.49	0.003	1.78	1.15-2.76	0.01
**Socio-economic status, 2018**								
Low	1		1			1		
Middle	1.01	0.60-1.70	0.97	0.58-1.63		0.98	0.58-1.67	
High	0.87	0.52-1.47	0.8	0.47-1.36	0.552	0.86	0.50-1.48	0.753
**Urban or rural**								
Rural	1		1			1		
Peri-urban/urban	0.7	0.48-1.02	0.67	0.46-0.97	0.035	0.71	0.48-1.05	0.088
**Invited or received DREAMS, 2017/18**								
No	1		1			1		
Yes	1.13	0.81-1.59	1.13	0.80-1.59	0.486	0.97	0.67-1.40	0.875

For peer review only

Supplementary Table 4 Exposure to MTV Shuga and HIV testing in the nested cohort of AGYW aged 13-22 (n=2184)

	Unadjusted		Shuga adjusted			Adjusted-All		
	**OR**	**95%CI**	**OR**	**95%CI**	**p-value**	**OR**	**95%CI**	**p-value**
**Ever watched MTV Shuga, 2018/19**								
No	1					1		
Yes	0.93	0.71-1.21				1.02	0.77-1.36	0.885
**Age**	1.3	1.25-1.35	1.3	1.24-1.35	<0.001	1.3	1.24-1.37	<0.001
**Currently in school**								
No	1		1			1		
Yes	0.37	0.28-0.48	0.37	0.28-0.48	0	1.07	0.74-1.53	0.726
**Socio-economic status, 2018**								
Low	1		1			1		
Middle	0.67	0.48-0.94	0.67	0.48-0.95		0.71	0.49-1.02	
High	0.58	0.41-0.81	0.58	0.41-0.81	0.007	0.6	0.41-0.86	0.018
**Urban or rural**								
Rural	1		1			1		
Peri-urban/urban	0.84	0.68-1.03	0.84	0.69-1.04	0.104	0.89	0.71-1.11	0.298
**Invited or received DREAMS, 2017/18**								
No	1		1			1		
Yes	0.84	0.69-1.02	0.84	0.69-1.02	0.081	0.98	0.79-1.23	0.89

For peer review only

Supplementary Table 5: Exposure to MTV Shuga with HIV prevention and SRHR awareness and uptake in the cross-sectional analysis of AGYW aged 12-24 (n=4127)

	aOR (95% Confidence Interval)	P-value
**District**		
City of Johannesburg	1	
Ekurhuleni	0.66 (0.47-0.91)	0.013
eThekwini	0.78 (0.54-1.13)	0.186
**Age group**		
12-14	1	
15-19	1.75 (0.35-8.84)	0.499
20-24	1.60 (0.32-8.07)	0.570
**Highest education level**		
Grade R to 7	1	
No schooling	3.84 (1.05-14.02)	0.041
Grade 8 to 12	3.17 (0.93-10.77)	0.065
Complete or incomplete tertiary	6.52 (1.90-22.33)	0.003
**Ever had sex with a boy/man**		
No	1	
Yes	0.67 (0.32-1.40)	0.286
**Away from home**		
No	1	
Yes	1.48 (0.93-2.37)	0.099
**PrEP awareness**		
No	1	
Yes	1.71 (1.20-2.43)	0.003
**Contraception use**		
No	1	
Yes	0.95 (0.74-1.23)	0.718
**Condom use at last sex**		
No	1	
Yes	1.19 (0.92-1.55)	0.183
**HIV test (self-report)**		
No	1	
Yes	0.88 (0.63-1.24)	0.471

For peer review only

Supplementary Table 6 Estimated causal effect of MTV Shuga on HSV-2 incidence, overall and by age group

Estimated causal effect of MTV Shuga on HSV-2 incidence, overall and by age group											
	% Outcome in total study population	Estimated % Outcome if no Shuga	95% CI	Estimated % Outcome if all get Shuga	95% CI	Risk Difference (%; PS adjusted)	95% CI	Prevalence Ratio (%; PS adjusted)	95% CI	Odds Ratio (%; PS adjusted)	95% CI
PS adjustment: Primary results											
Overall	17.3	17.2	14.71 - 19.67	18.0	14.51 - 22.32	0.80	-3.24 - 5.71	1.05	0.82 - 1.36	1.06	0.79 - 1.46
13-17 Years	13.2	12.4	9.85 - 15.16	15.7	11.63 - 20.26	3.34	-1.60 - 8.25	1.27	0.89 - 1.79	1.32	0.87 - 1.98
18-22 Years	24.3	25.8	21.01 - 30.74	22.0	15.19 - 30.17	-3.76	-11.92 - 5.68	0.85	0.59 - 1.24	0.81	0.51 - 1.34
**Sensitivity analyses**											
Regression under counterfactual framework											
Overall	17.3	17.2	14.74 - 19.66	17.8	14.50 - 22.08	0.65	-3.42 - 5.72	1.04	0.82 - 1.36	1.05	0.78 - 1.46
13-17 Years	13.2	12.42	9.86 - 15.21	15.9	11.85 - 20.49	3.47	-1.54 - 8.63	1.28	0.89 - 1.80	1.33	0.88 - 1.99
18-22 Years	24.3	25.7	20.78 - 30.63	21.3	14.88 - 28.28	-4.42	-12.28 - 4.95	0.83	0.57 - 1.22	0.78	0.48 - 1.31
PS stratification											
Overall	17.3	17.1	14.68 - 19.68	17.9	14.53 - 22.13	0.80	-3.21 - 5.52	1.09	0.84 - 1.40	1.11	0.82 - 1.51
PS weighting											

For peer review only

Overall	17.3	17.08	14.74 - 19.48	17.22	13.99 - 21.10	0.13	-3.76 - 4.63	1.01	0.79 - 1.30	1.01	0.76 - 1.37

For peer review only

STROBE Statement—checklist of items that should be included in reports of observational studies

	Item No	Recommendation	Page No
**Title and abstract**	1	(*a*) Indicate the study's design with a commonly used term in the title or the abstract	1
		(*b*) Provide in the abstract an informative and balanced summary of what was done and what was found	2
**Introduction**			
Background/rationale	2	Explain the scientific background and rationale for the investigation being reported	4
Objectives	3	State specific objectives, including any prespecified hypotheses	6
**Methods**			
Study design	4	Present key elements of study design early in the paper	6-7
Setting	5	Describe the setting, locations, and relevant dates, including periods of recruitment, exposure, follow-up, and data collection	7
Participants	6	(*a*) *Cohort study*—Give the eligibility criteria, and the sources and methods of selection of participants. Describe methods of follow-up<br>*Case-control study*—Give the eligibility criteria, and the sources and methods of case ascertainment and control selection. Give the rationale for the choice of cases and controls<br>*Cross-sectional study*—Give the eligibility criteria, and the sources and methods of selection of participants	7
		(*b*) *Cohort study*—For matched studies, give matching criteria and number of exposed and unexposed<br>*Case-control study*—For matched studies, give matching criteria and the number of controls per case	
Variables	7	Clearly define all outcomes, exposures, predictors, potential confounders, and effect modifiers. Give diagnostic criteria, if applicable	8-9
Data sources/ measurement	8*	For each variable of interest, give sources of data and details of methods of assessment (measurement). Describe comparability of assessment methods if there is more than one group	8-10
Bias	9	Describe any efforts to address potential sources of bias	9-10
Study size	10	Explain how the study size was arrived at	6-7
Quantitative variables	11	Explain how quantitative variables were handled in the analyses. If applicable, describe which groupings were chosen and why	8
Statistical methods	12	(*a*) Describe all statistical methods, including those used to control for confounding	9-10
		(*b*) Describe any methods used to examine subgroups and interactions	10
		(*c*) Explain how missing data were addressed	10
		(*d*) *Cohort study*—If applicable, explain how loss to follow-up was addressed<br>*Case-control study*—If applicable, explain how matching of cases and controls was addressed<br>*Cross-sectional study*—If applicable, describe analytical methods taking account of sampling strategy	10
		(*e*) Describe any sensitivity analyses	10

Continued on next page

For peer review only - http://bmjopen.bmj.com/site/about/guidelines.xhtml

For peer review only

**Results**			
Participants	13*	(a) Report numbers of individuals at each stage of study—eg numbers potentially eligible, examined for eligibility, confirmed eligible, included in the study, completing follow-up, and analysed	11
		(b) Give reasons for non-participation at each stage	11
		(c) Consider use of a flow diagram	11
Descriptive data	14*	(a) Give characteristics of study participants (eg demographic, clinical, social) and information on exposures and potential confounders	11
		(b) Indicate number of participants with missing data for each variable of interest	
		(c) *Cohort study*—Summarise follow-up time (eg, average and total amount)	
Outcome data	15*	*Cohort study*—Report numbers of outcome events or summary measures over time	12
		Case-control study—Report numbers in each exposure category, or summary measures of exposure	
		Cross-sectional study—Report numbers of outcome events or summary measures	12
Main results	16	(*a*) Give unadjusted estimates and, if applicable, confounder-adjusted estimates and their precision (eg, 95% confidence interval). Make clear which confounders were adjusted for and why they were included	12-16
		(*b*) Report category boundaries when continuous variables were categorized	12-16
		(*c*) If relevant, consider translating estimates of relative risk into absolute risk for a meaningful time period	
Other analyses	17	Report other analyses done—eg analyses of subgroups and interactions, and sensitivity analyses	17
**Discussion**			
Key results	18	Summarise key results with reference to study objectives	17-18
Limitations	19	Discuss limitations of the study, taking into account sources of potential bias or imprecision. Discuss both direction and magnitude of any potential bias	18
Interpretation	20	Give a cautious overall interpretation of results considering objectives, limitations, multiplicity of analyses, results from similar studies, and other relevant evidence	18-19
Generalisability	21	Discuss the generalisability (external validity) of the study results	17-19
**Other information**			
Funding	22	Give the source of funding and the role of the funders for the present study and, if applicable, for the original study on which the present article is based	20

*Give information separately for cases and controls in case-control studies and, if applicable, for exposed and unexposed groups in cohort and cross-sectional studies.

Note: An Explanation and Elaboration article discusses each checklist item and gives methodological background and published examples of transparent reporting. The STROBE checklist is best used in conjunction with this article (freely available on the Web sites of PLoS Medicine at http://www.plosmedicine.org/, Annals of Internal Medicine at http://www.annals.org/, and Epidemiology at http://www.epidem.com/). Information on the STROBE Initiative is available at www.strobe-statement.org.

For peer review only - http://bmjopen.bmj.com/site/about/guidelines.xhtml

BMJ Open

Evaluating use of mass-media communication intervention 'MTV-Shuga' on increased awareness and demand for HIV and sexual health services by adolescent girls and young women in South Africa: An observational study

Journal:	*BMJ Open*
Manuscript ID	bmjopen-2022-062804.R3
Article Type:	Original research
Date Submitted by the Author:	21-Apr-2023
Complete List of Authors:	Chimbindi, Natsayi; Africa Health Research Institute; University College London,<br>Mthiyane, Nondumiso; Africa Health Research Institute<br>Chidumwa, Glory ; Africa Health Research Institute; Wits Reproductive Health and HIV Institute, Faculty of Health Sciences, University of the Witwatersrand<br>Zuma, Thembelihle; Africa Health Research Institute; University College London,<br>Dreyer, Jaco; Africa Health Research Institute<br>Birdthistle, Isolde; London School of Hygiene and Tropical Medicine Faculty of Epidemiology and Population Health, Department of Population Health<br>Floyd, Sian; London School of Hygiene & Tropical Medicine, Infectious Disease Epidemiology<br>Kyegombe, Nambusi; London School of Hygiene & Tropical Medicine<br>Grundy, Chris; London School of Hygiene and Tropical Medicine Faculty of Epidemiology and Population Health, Department of Population Health<br>Cawood, Cherie; Epicentre Health Research<br>Danaviah, Siva; Africa Health Research Institute<br>Smit, Theresa; Africa Health Research Institute<br>Pillay, Deenan; University College London,<br>Baisley, Kathy; London School of Hygiene and Tropical Medicine Faculty of Epidemiology and Population Health; Africa Health Research Institute<br>Harling, Guy; Africa Health Research Institute; University College London<br>Seeley, Janet; London School of Hygiene and Tropical Medicine, Department of Global Health &Development; Africa Health Research Institute<br>Shahmanesh, Maryam; University College London, Institute for Global Health; Africa Health Research Institute,
**Primary Subject Heading**:	Sexual health
Secondary Subject Heading:	HIV/AIDS, Public health, Sexual health
Keywords:	HIV & AIDS < INFECTIOUS DISEASES, PUBLIC HEALTH, PREVENTIVE MEDICINE

I, the Submitting Author has the right to grant and does grant on behalf of all authors of the Work (as defined in the below author licence), an exclusive licence and/or a non-exclusive licence for contributions from authors who are: i) UK Crown employees; ii) where BMJ has agreed a CC-BY licence shall apply, and/or iii) in accordance with the terms applicable for US Federal Government officers or employees acting as part of their official duties; on a worldwide, perpetual, irrevocable, royalty-free basis to BMJ Publishing Group Ltd ("BMJ") its licensees and where the relevant Journal is co-owned by BMJ to the co-owners of the Journal, to publish the Work in this journal and any other BMJ products and to exploit all rights, as set out in our licence.

The Submitting Author accepts and understands that any supply made under these terms is made by BMJ to the Submitting Author unless you are acting as an employee on behalf of your employer or a postgraduate student of an affiliated institution which is paying any applicable article publishing charge ("APC") for Open Access articles. Where the Submitting Author wishes to make the Work available on an Open Access basis (and intends to pay the relevant APC), the terms of reuse of such Open Access shall be governed by a Creative Commons licence – details of these licences and which Creative Commons licence will apply to this Work are set out in our licence referred to above.

Other than as permitted in any relevant BMJ Author's Self Archiving Policies, I confirm this Work has not been accepted for publication elsewhere, is not being considered for publication elsewhere and does not duplicate material already published. I confirm all authors consent to publication of this Work and authorise the granting of this licence.

For peer review only

Title page

Revised Title: **Evaluating use of mass-media communication intervention 'MTV-Shuga' on increased awareness and demand for HIV and sexual health services by adolescent girls and young women in South Africa: An observational study**

Authors: Natsayi Chimbindi*[1,2,3], Nondumiso Mthiyane*[2], Glory Chidumwa[1,4], Thembelihle Zuma[1,2,3], Jaco Dreyer[1], Isolde Birdthistle[5], Sian Floyd[5], Nambusi Kyegombe[5], Chris Grundy[5], Cherie Cawood[6], Siva Danaviah[1], Theresa Smit[1], Deenan Pillay[2], Kathy Baisley[1,3], Guy Harling[1,2,3,7,], Janet Seeley[1,3,5], Maryam Shahmanesh§[1,2,3]

1. Africa Health Research Institute, KwaZulu-Natal, South Africa
2. University College London, Institute for Global Health, London, UK
3. University of KwaZulu-Natal, KwaZulu-Natal, South Africa
4. Wits Reproductive Health and HIV Institute, Faculty of Health Sciences, University of the Witwatersrand, Johannesburg, South Africa
5. London School of Hygiene and Tropical Medicine, London, UK
6. Epicentre Health Research, Durban, South Africa
7. MRC/Wits Rural Public Health & Health Transitions Research Unit (Agincourt), University of the Witwatersrand, Gauteng, South Africa

*Joint authors

§Corresponding author (full address details): Maryam Shahmanesh, Institute for Global Health, UCL, 3rd Floor, Mortimer Market Centre, Capper Street, London WC1E 6JB. m.shahmanesh@ucl.ac.uk. +44 7776 185 572

Key words (6): HIV, Adolescents, mass communication campaigns, Sexual Health, Teenage Pregnancy, South Africa.

Abstract (word count=272)

Objective: To investigate the effect of exposure to MTV Shuga:Down South' (MTVShuga-DS)

For peer review only

during the scale-up of combination HIV-prevention interventions on awareness and uptake of sexual reproductive health (SRH) and HIV-prevention services by adolescent girls and young women (AGYW).

Design: One longitudinal and three cross-sectional surveys of representative samples of AGYW.

Setting: AGYW in four South African districts with high HIV-prevalence (>10%) (May 2017 and September 2019).

Participants: 6311 AGYW aged 12-24.

Measures: Using logistic-regression we measured the relationship between exposure to MTV Shuga-DS and awareness of Pre-Exposure Prophylaxis (PrEP), condom-use at last sex, uptake of HIV-testing or contraception, and incident pregnancy or Herpes simplex virus 2 (HSV-2) infection.

Results: Within the rural cohort 2184 (85.5%) of eligible sampled individuals were enrolled, of whom 92.6% had at least one follow-up visit; the urban cross-sectional surveys enrolled 4127 (22.6%) of eligible sampled individuals. Self-report of watching at least one MTV Shuga-DS episode was 14.1% (cohort) and 35.8% (cross-section), while storyline recall was 5.5% (cohort) and 6.7% (cross-section).

In the cohort, after adjustment (for HIV-prevention intervention-exposure, age, education, socio-economic status (SES)), MTVShuga-DS exposure was associated with increased PrEP awareness (adjusted odds ratio (aOR)=2.06,95% confidence interval (CI):1.57-2.70), contraception uptake (aOR=2.08,95%CI:1.45-2.98) and consistent condom-use (aOR=1.84, 95%CI:1.24-2.93), but not with HIV-testing (aOR=1.02,95%CI:0.77-1.21) or acquiring HSV-2 (aOR=0.92, 95%CI:0.61-1.38). In the cross-sections, MTVShuga-DS was associated with greater PrEP awareness (aOR=1.7, 95%CI:1.20-2.43), but no other outcome.

Conclusions: Among both urban and rural AGYW in South Africa, MTVShuga-DS exposure was associated with increased PrEP awareness and improved demand for some HIV-prevention and SRH technologies but not sexual health outcomes. However, exposure to MTVShuga-DS was low. Given these positive indications, supportive programming may be required to raise exposure and allow future evaluation of edu-drama impact in this setting.

For peer review only - http://bmjopen.bmj.com/site/about/guidelines.xhtml

Strengths and limitations of this study

- Evaluated the real-world reach of nationally broadcast edu-drama focusing on adolescent sexual health in South Africa.
- Data collection focused on a vulnerable population of AGYW across four diverse high HIV-burden districts of South Africa that included both rural and urban settings.
- Strength of the study is measurement of exposure to MTV Shuga on HIV and SRH outcomes in representative sample of AGYW during a real-world implementation in different South African settings.
- The limitation of observational studies is that they do not infer the cause–effect relationship, in this case we cannot ascertain causality/impact of exposure to MTV-Shuga on uptake of sexual health promotion and innovative technologies.

For peer review only

For peer review only - http://bmjopen.bmj.com/site/about/guidelines.xhtml

[word count 4531]

Introduction

HIV remains one of the gravest health problems facing young people living in sub-Saharan Africa (SSA). There are over 7.7 million people living with HIV in South Africa (SA), with more than 200,000 new HIV infections annually in those aged 15-49 years (1). The highest incidence is in adolescent girls and young women (AGYW) (15-24 years) (1, 2). Although HIV incidence has been declining in South Africa, a 43% decline in the overall incidence rate between 2012 and 2017, from 4.0 to 2.3 seroconversion events per 100 person-years among 15-49 year old; it still remains high among AGYW in South Africa (3). In uMkhanyakude, HIV incidence was lower during roll-out of combination HIV prevention for AGYW (2016 to 2018) than in the previous 5-year period among 15- to 19-year-old females (4.5 new infections per 100 person-years as compared with 2.8; and lower among 20- to 24-year-olds (7.1/100 person-years as compared with 5.8) (4).

In response to the high HIV incidence in young people, the South African government launched the `She Conquers Campaign', and the US President's Emergency Fund for AIDS Relief and others are supporting the roll-out of Determined, Resilient, Empowered, AIDS free, Mentored, and Safe (DREAMS)(5-7). These programmes provide an evidence-based combination HIV prevention package, including HIV-testing and counselling for adolescent girls and young women (AGYW) and their male sexual partners, alongside universal test and treat and improved sexual health services (8, 9).

However, the key ingredient to the success of these multicomponent interventions will be the extent to which AGYW and their male partners at most risk of HIV will uptake and adhere to the active components of the intervention. This is challenging: uptake and retention of contraception and HIV treatment cascade by young people, even within population-wide Universal Test and Treat trials, has been suboptimal (2, 10, 11). Data from the baseline analysis for the DREAMS impact evaluation in uMkhanyakude district, rural KwaZulu-Natal (KZN) in 2015 suggest that less than 40% of girls (15-19 years) and boys and young men (15-29 years) had ever tested for HIV; linkage to HIV treatment was even lower (12). Contraception use prevalence was 20% in girls (15-19 years) and 50% in young women (20-24 years) and 21% of 15-19 year old girls had ever been pregnant (2).

It is against this backdrop that the fifth series of Shuga: 'MTV Shuga: Down South' (MTV Shuga-DS), a mass media serial edu-drama designed for SA, was broadcast on free-to-air South Africa National television. From March 8th 2017, MTV Shuga aired one episode per week for 12 weeks (with repeats). MTV Shuga is a mass media behaviour change campaign

For peer review only

that aims to improve sexual and reproductive health rights (SRHR). At the centre of the campaign, which includes radio and social media, is a TV-drama that weaves messages about HIV, family planning, transactional and intergenerational sex, sexual identity, safer and healthy sexual relationships, into storylines with young characters (http://www.mtvshuga.com/show/series-5/MTV Shuga-down-south/).

Mass media campaigns, have the potential to reach a large number of people and have been shown to improve knowledge and health behaviour of a range of health conditions, with more recent data suggesting that theoretically informed and targeted interventions are more likely to have an effect (13, 14). MTV Shuga-DS was designed to reduce HIV-related risk behaviour and improve SRHR outcomes in adolescents and young adults in SA. This was expected to be achieved through increasing young people's awareness of their sexual and reproductive health rights and demand for, and uptake of HIV and sexual reproductive health (SRH) prevention and treatment technologies. The show's characters explicitly model how to discuss issues that are sensitive or taboo. MTV Shuga use the technique of 'melodramas', where drama is created through the battles between stereotypical goodies and baddies, and the way in which the 'transitional' (often empathetic) character, begins as ambivalent but changes into a positive role model to promote positive behaviour change(15). This is a deliberate method to immerse the audience in the action, rather than passively watching or listening (16). AGYW, or at least early adopters, are anticipated to be immersed in the serial, able to classify and identify with the transitional characters and their outcomes. Pathways to behaviour change through MTV Shuga, therefore relate to the extent to which the observer, including early adopters, are immersed and critically engaged with the story. It also depends on a context which is supportive rather than disruptive (see the conceptual framework Figure 1 below) (17).

Figure 1 showing conceptual framework for MTV Shuga impact on HIV prevention on AGYW in uMkhanyakude

A cluster randomised controlled trial of community-viewings of MTV Shuga in Nigeria found that exposure to MTV Shuga significantly improved HIV knowledge and testing in both sexes, the intervention arm showing 35% more likely to test for HIV than the control arm. There was also a 60% reduction in genital chlamydia as a marker of recent sexual risk in women, amongst those exposed to MTV Shuga compared to those who were not (18). There were, however, fewer changes in social norms, particularly around gender-based violence. Further work suggested that the impact was greatest in those who were immersed in the narrative. The importance of immersion (classification of characters and identifying with them and observing outcomes) coupled with critical participation and an enabling context were also found in a

For peer review only - http://bmjopen.bmj.com/site/about/guidelines.xhtml

thematic analysis looking at how storylines in MTV Shuga-DS shaped awareness, knowledge and opinions of sexual health and personal relationships among young people in SA (17).

Whilst the Nigerian and SA studies provide evidence for the efficacy of MTV Shuga impacting on SRHR and HIV-testing behaviours in exposed individuals, there is little evidence of how this will translate into a population-level effect when nationally broadcast and in less controlled environments. In particular, it is not clear how the impact will spill-over to non-viewers and how innovations will diffuse when shown and watched by adolescents and young adults in a real-world scale-up. It is also unclear how such impact will differ according to: setting (rural or urban); differential digital literacy and access to social media (geographically and socioeconomically), and the dose and context of watching (shared viewing with family, friends or individual experience through social media).

We use the opportunity of MTV Shuga being broadcast in the context of an impact evaluation of DREAMS roll out (January 2016-September 2019) in a representative, population-based sample of young people (12) to describe the real-world reach of nationally broadcast MTV Shuga-DS. Further, we explore the hypothesis that exposure to a mass-media serial edu-drama, like MTV Shuga, will improve SRHR outcomes by increasing demand for, and uptake of, existing combination individual and community-based SRH and HIV prevention services for AGYW in four diverse settings including uMkhanyakude, a socioeconomically deprived rural district with an extremely high burden of HIV: 40% antenatal HIV prevalence and an annual HIV incidence of 5% in girls (15-19 years) and 8% in young women (20-24 years) (2, 19) and in three high-prevalence urban districts (HIV prevalence of greater than 10%) of City of Johannesburg, Ekurhuleni, and eThekwini.

Methods

Study design

We employed a longitudinal cohort and cross-sectional surveys of representative samples of AGYW aged 12-24 in four districts of South Africa with a high burden of HIV to measure the reach of MTV Shuga-DS. Data were collected between May 2017 and September 2019.

We used baseline and follow-up data from a nested cohort of 2184 AGYW aged 13-22 years, enrolled in 2017 for the DREAMS impact evaluation. The cohort is nested in a large population-based longitudinal HIV surveillance study, in the uMkhanyakude district of KwaZulu-Natal (20, 21). A random sample of 3013 AGYW was selected from the surveillance

For peer review only - http://bmjopen.bmj.com/site/about/guidelines.xhtml

population, stratified by age (13-17 years and 18-22 years) and geography, and invited to enrol in the nested cohort. Baseline interviews were conducted between May 2017 and February 2018 and follow-up interviews April 2018 and September 2019 in the local language (isiZulu) using a structured quantitative questionnaire programmed in REDCap software (12).

The cross-sectional survey was conducted on a household-based representative sample of 4127 AGYW (between the ages 12-24 years) in three high prevalence (City of Johannesburg, Ekurhuleni and eThekwini) districts. Between August 2017 and July 2018, a stratified cluster-based sampling strategy was used to select 18500 AGYW aged 12-24 eligible for a cross-sectional survey of individuals, based on an expected response rate of 80% (22).

The interview included questions on socio-demographics, general health, exposure to DREAMS and to MTV Shuga, sexual relationships, awareness and uptake of DREAMS and DREAMS-like services, migration, and gender norms across the four districts. A Dried Blood Spot (DBS) was taken at baseline and follow-up for Herpes Simplex Virus type 2 (HSV-2) antibody testing in the uMkhanyakude district. For sexual behaviour questions, participants were given a tablet computer to complete a self-interview; the fieldworker was available to provide support as needed.

Study setting and population

The cohort was nested within the Africa Health Research Institute (AHRI) triannual demographic surveillance of a population of approximately 150,000 people who are members of 15,000 geocoded households in an area of 845 km^2 (20). The study area is mostly rural and poor with high levels of youth unemployment (over 85% of those aged 18-24 are unemployed) (2, 19).

The cross-sectional survey was conducted in three districts which were mostly urban with more towns and townships compared to AHRI surveillance area. The three study districts (City of Johannesburg, Ekurhuleni and eThekwini) consist of an estimated 12,073,421 individuals. The eThekwini district in KZN province is among those with the highest HIV prevalence (16.8% HIV prevalence in 2016) in South Africa. Over two-thirds (68%) of eThekwini is considered rural and 32% urban. About 11 963 (3%) AGYW in eThekwini are estimated to be living with HIV. The Gauteng province (GP), whilst geographically the smallest, is the most populous province in South Africa. GP has the fifth highest provincial HIV prevalence in the country with a prevalence of 11.1% among those aged 15 to 49 years old in 2016. The HIV prevalence in the two districts, City of Johannesburg and Ekurhuleni, is 11.1% and 14.3% among 15- to 49-

For peer review only - http://bmjopen.bmj.com/site/about/guidelines.xhtml

year-olds, respectively. Both districts are densely populated and have high levels of industrialisation. The HIV prevalence amongst AGYW (15 to 24 years old) in the City of Johannesburg is 3%, and similarly 3% in Ekurhuleni in 2012.

Variables and Measurement:

Outcomes definitions:

We measured the effect of exposure to MTV Shuga on awareness and uptake of HIV prevention and SRHR outcomes at the follow-up visit. Our outcomes were: 1) self-reported HIV-testing in the past 12 months; 2) awareness of Pre-Exposure Prophylaxis (PrEP) for HIV prevention; 3) condom use at last sex; 4) use of contraception; 5) any new pregnancy since baseline; and 6) any new teenage pregnancy (restricted to those under the age of 20). AGYW were considered to use contraception if they self-reported using pill, injection, Intrauterine Device (IUD), implant, sterilisation or self-reported consistent condom use (using condoms as a contraceptive method and at last sex). Condomless sex was calculated using 2019 data among participants who reported having had sex with the most recent partner in the past 12 months. Recent pregnancy was calculated as any new pregnancy that occurred between baseline and 2019, while teenage pregnancy was calculated as any new pregnancy that occurred between baseline and 2019 among participants aged below 20 years. We also examined the effect of exposure to MTV Shuga on incident HSV-2 infections, among those who were HSV-2 negative at baseline.

Exposure definitions:

Exposure to MTV Shuga was defined as ever watched MTV Shuga between 2017 and 2018. The level of exposure was measured based on the content of the series MTV Shuga, defined using 15 questions used to assess knowledge of content of MTV Shuga series. A composite score was developed summing-up the correct responses. The scores ranged between 2 and 14, and the median being 4. The median was used as a cut-off to define level of exposure among those who watched the series. Consequently, the level of exposure was categorized into 3 levels: High (watching a MTV Shuga and being able to correctly respond to 5 or more questions on content); Medium (watching programme and being able to correctly respond to less than 5 questions); and None (not watched any MTV Shuga). This was further categorised into 3 levels: High (watching MTV Shuga and being able to recall the content from MTV Shuga); Medium (watching programme, but unable to recall content); and None (not being aware of and not watched any MTV Shuga).

For peer review only

Potential confounding variables:

We included socio-demographic and sexual behaviour characteristics of AGYW that were measured at baseline and exposure to HIV prevention. The socio-demographic variables included age (as measured at follow-up), household socio-economic status (SES), education broken down by those who are still in school and those who have completed school, geographic area (rural or peri-urban/urban), and migration in the last 12 months. The socio-economic status (SES) variable was constructed using Principal Component Analysis (PCA) based on ownership of household assets and characteristics such as access to piped water, type of toilet, electricity and type of cooking fuel (23). Further, potential individual-level confounders measured included exposure to DREAMS (defined either as ever been invited to participate in any of the DREAMS activities or ever used any of the DREAMS HIV prevention interventions in the past 12 months or since 2016) and phone ownership at baseline.

Laboratory:

The HerpeSelect® 2 ELISA IgG assay (FOCUS Diagnostics, Cypress, California, USA) for the qualitative detection of human Immunoglobulin G (IgG) class antibodies to HSV-2 was used on Dried blood Spot (DBS) samples collected on Whatman 903 filter cards. The HerpeSelect ® 2 ELISA IgG assay uses purified type-specific gG-2 antigen immobilized on polystyrene microwells reducing the cross-reactivity issues as seen with viral lysate assays(24). The assay is validated for use with serum samples but was optimised for use with DBS in the AHRI Diagnostic Research Laboratory following comparative testing with plasma samples. During the initial evaluation of the HerpeSelect® 2 ELISA IgG a select number of plasma samples were also tested by an external accredited pathology laboratory. A 6mm diameter punch of a DBS spot was incubated overnight in 150ul Assay Diluent for no more than 16 hours at 4°C. The assay was performed with 50ul of the eluent in accordance with the manufacturer's instructions. Following a disproportionately high number of positive results based on other studies and our experience we multiplied the mean cut-off calibrator absorbance values by a factor of 1.5 before determining the index value for each sample (25, 26). The HerpeSelect ® 2 ELISA IgG results are reported as positive (index value >1.10), equivocal (index value of ≥ 0.90 but ≤1.10) or negative (index value <0.90). All initial equivocal results were re-tested and those that re-tested equivocal are reported as equivocal. An incident HSV-2 was defined as having been negative at baseline and positive at follow-up. Those who were equivocal at either baseline or follow-up were not considered as a sero-conversions.

For peer review only

Statistical analyses:

We conducted two separate analyses for cohort and cross-sectional data. For the nested cohort, we included only participants who had data available at baseline and follow-up. We used Chi-square tests to compare baseline characteristics between AGYW who did and did not have any exposure to MTV Shuga. We used logistic regression to examine the effect of MTV Shuga on health outcomes, adjusting for exposure to DREAMS and all other potential confounders. Potential effect-modification of MTV Shuga by exposure to DREAMS was examined by fitting an interaction term to fully adjusted model: likelihood ratio tests were used to compare models with and without interaction terms.

We calculated the proportion of AGYW who reported an outcome (condomless-sex, recent pregnant) or tested positive for HSV-2 at 12-month or 24-month follow-up; and estimated associations between MTV Shuga and each outcome using a logistic regression, adjusting for potential confounders (age, household and individual socio-demographic characteristics and sexual behaviour). For HSV-2 incidence, we included participants who tested negative at baseline and had at least 1 follow-up test result. For DREAMS exposure, we included data collected at baseline and 12-month follow-up. For health outcomes (consistent condom use, modern contraception, HIV testing, PrEP awareness), we used data collected at 24-month follow-up; and for HSV-2 and pregnancy incidence we used data collected at 12 and 24-month follow-up.

Propensity score logistic regression adjustment was used to estimate the causal effect of MTV Shuga on health outcomes. A propensity or probability of being exposed to MTV Shuga was measured by fitting a logistic regression with MTV Shuga exposure as an outcome and potential confounders. A logistic regression models adjusting for propensity scores were then used to predict the probability of an outcome for all participants and separately by age group, under two scenario (1) exposed to MTV Shuga and (2) Not exposed to MTV Shuga. The predicted probabilities were then used to calculate the marginal risk difference, prevalence ratio and odds ratio (OR). Confidence intervals (CI) were generated by using a bootstrap procedure, repeating the estimation procedure described above in 1000 samples that were drawn with replacement from the complete dataset and calculating 95% confidence intervals from the resulting bootstrap distribution using the 2.5% and 97.5% percentiles. We also used propensity score stratification and probability weighting methods to check the consistency of our findings.

For peer review only

For peer review only - http://bmjopen.bmj.com/site/about/guidelines.xhtml

For cross-sectional survey, we used Chi square test to compare the characteristics of AGYW who did and did not have any exposure to MTV Shuga; and logistic regression models adjusting for potential confounders were used to examine the association between exposure to MTV Shuga and health outcomes. Sampling weights were applied to achieve proportionality between groups of participants in the survey.

All analyses were performed using Stata version 15 (StataCorp LP, College Station, Texas USA).

Reporting

The STROBE reporting guidelines were used to guide synthesis and standardise reporting of our results(27)

Patient and public involvement

The study did not involve patients. AHRI has a Public Engagement Unit which conducts community engagement activities with the local communities as part of study findings dissemination. The Community Advisory Board provided feedback of the study including design before approval from ethics review board. Study findings are being made publicly available to funders, participants and the public through webinars, study reports and open access journal articles.

Results:

Participants

Of 3013 potentially eligible AGYW randomly selected from the surveillance data set, 85.5% of those eligible consented to participate at baseline (Figure 2). Of the 2184 eligible participants that were surveyed at baseline, 2016 (92.3%) had at least one follow-up visit and contributed data to this analysis. From the cross-sectional survey, 4127 (22.6%) eligible participants were surveyed.

Figure 2 Flowchart showing AGYW follow-up from nested cohorts 2017-2019

Awareness and exposure to MTV Shuga

MTV Shuga exposure at baseline was limited, with a total of only 308 (14.1%) respondents reported watching at least one episode. In the cross-sectional analysis of the three districts, a total of 1477 (35.8%) reported watching any MTV Shuga. In the nested cohort 121 (5.5%)

recalled any storyline. Similarly in the cross-sectional snapshot 276 (6.7%) recalled the storyline (high exposure).

Social demographic characteristics of adolescents and young people by exposure to MTV Shuga (Table 1&2)

Table 1 summarises the profiles of the nested cohort (n=2184) and cross-sectional surveys (n=4127) AGYW comparing those exposed to MTV Shuga with those not exposed. In summary those who had seen any MTV Shuga were more likely to be from households in the highest socioeconomic tertile (p<0.001) and more urbanised areas (p<0.001). They were also more likely to have also received DREAMS (p=0.015) (Table 1). In the cross-sectional surveys (n=4127), MTV Shuga exposure was associated with older age (p<0.001), tertiary education (p <0.001) and never having sex (p< 0.001) Table 1.

Table 1. Baseline socio-demographic characteristics of adolescent girls and young women (13-22) by exposure to MTV Shuga in the nested cohort (n=2184)

	Overall		Exposed		Not exposed		
	n/N	%	n/N	%	n/N	%	p-value
**Age group (4 cats), 2017**							
13-14	460/2184	21.1	72/308	23.4	388/1876	20.7	
15-17	688/2184	31.5	107/308	34.7	581/1876	31	
18-19	475/2184	21.7	60/308	19.5	415/1876	22.1	
20-22	561/2184	25.7	69/308	22.4	492/1876	26.2	0.216
**Currently in school**							
No	540/2184	24.7	67/308	21.8	473/1876	25.2	
Yes	1644/2184	75.3	241/308	78.2	1403/1876	74.8	0.192
**Socio-economic status, 2018**							
Low	255/2118	12	22/303	7.3	233/1815	12.8	
Middle	920/2118	43.4	110/303	36.3	810/1815	44.6	
High	943/2118	44.5	171/303	56.4	772/1815	42.5	<0.001
**Urban or rural**							
Rural	1388/2165	64.1	165/305	54.1	1223/1860	65.8	
Peri-urban/urban	777/2165	35.9	140/305	45.9	637/1860	34.2	<0.001
**Invited or received DREAMS, 2017/18**	1101/2184	50.4	175/308	56.8	926/1876	49.4	0.015
**Away from home in the last 12 months**	314/1853	17.0	45/228	15.6	269/1565	17.2	0.516
**Baseline socio-demographic characteristics of adolescent girls and young women (12-24) by exposure to MTV Shuga in the cross-sectional survey (n=4127)**							
	Overall		Exposed		Not exposed		

For peer review only - http://bmjopen.bmj.com/site/about/guidelines.xhtml

	n/N	%	n/N	%	n/N	%	p-value
**Age group, (N=4127)**							
12-14	958/4127	23.2	307/1477	20.1	651/2650	24.6	
15-19	1628/4127	39.5	599/1477	40.6	1029/2650	38.8	
20-24	1541/4127	37.3	571/1477	38.7	970/2650	36.6	0.022
**District**							
City of Johannesburg	1146/4127	27.8	476/1477	32.2	670/2650	25.3	
Ekurhuleni	1635/4127	39.6	521/1477	35.3	1114/2650	42.0	
eThekwini	1342/4127	32.5	480/1477	32.5	862/2650	32.5	<0.001
**Highest Education (N=4108)**							
No schooling	175/4108	4.3	99/1465	6.8	76/2646	2.9	
Grade R to 7	502/4108	12.2	142/1465	9.7	360/2646	13.6	
Grade 8 to 12	2978/4108	72.5	1022/1465	69.8	1956/2646	74.0	
Tertiary studies (complete/incomplete)	453/4108	11.0	202/1465	13.8	251/2646	9.5	<0.001
**Ever had sex with a boy/man (n=4108)**	1860/4108	45.3	621/1469	42.3	1239/2639	47.0	0.004
**Away from home in the last 12 months (n=4121)**	183/4121	4.4	66/1474	4.5	117/2647	4.4	0.932

After adjustment for confounders in nested cohorts (Table 2), AGYW from wealthier households (adjusted Odds Ratio (aOR)=2.04 95%CI 1.27-3.30), peri-urban or urban areas (aOR=1.54 95%CI 1.19-1.98) and those invited to DREAMS (aOR=1.48 95%CI 1.14-1.92) were more likely to be exposed to MTV Shuga than those from poor households, from rural areas and those not invited to DREAMS respectively. Similarly, after adjustment in the cross-sectional surveys (Table 2), AGYW with higher education were more likely to be exposed to MTV Shuga (aOR=2.58 95%CI 1.81-3.69) than those with less and those who ever had sex (aOR=0.68 95%CI 0.57-0.82) were less likely to be exposed to MTV Shuga.

Table 2 Factors associated with exposure to MTV Shuga in the nested cohort of AGYW aged 13-22 (n=2184)

	Unadjusted		Adjusted - All		
	OR	95%CI	OR	95%CI	P-value
**Age group, 2017**					
13-14	1		1		
15-17	0.99	0.72 -1.37	0.93	0.66 -1.29	
18-19	0.78	0.54 -1.13	0.77	0.51 -1.14	
20-22	0.76	0.53 -1.08	0.78	0.50 -1.21	0.555
**Currently in school**					
No	1		1		
Yes	1.21	0.91 -1.62	1.06	0.78 -1.43	0.726

For peer review only - http://bmjopen.bmj.com/site/about/guidelines.xhtml

**Socio-economic status, 2018**					
Low	1		1		
Middle	1.44	0.89 -2.33	1.27	0.78 -2.07	
High	2.35	1.47 -3.74	2.04	1.27 -3.30	<0.001
**Site**					
Rural	1		1		
Peri-urban/ urban	1.63	1.28 -2.08	1.54	1.19 -1.98	0.001
**Invited or received DREAMS, 2017/18**					
No	1		1		
Yes	1.35	1.06 -1.72	1.48	1.14 -1.92	0.003

Factors associated with exposure to MTV Shuga in the cross-sectional analysis of AGYW aged 12-24 (n=4127)

	**Unadjusted**		**Adjusted - all**		
	**OR**	**95% CI**	**OR**	**95% CI**	**P-value**
**District**					
City of Johannesburg	1		1		
Ekurhuleni	0.69	0.54 - 0.89	0.66	0.52- 0.84	0.001
eThekwini	0.79	0.60 - 1.04	0.78	0.59- 1.04	0.087
**Age group**					
12-14	1		1		
15-19	1.27	1.06 - 1.53	1.18	0.95- 1.46	0.126
20-24	1.22	1.01 - 1.48	1.16	0.90- 1.49	0.256
**Highest education level**					
Grade R to 7	1		1		
No schooling	2.88	1.81 - 4.58	3.29	2.01 - 5.38	<0.001
Grade 8 to 12	1.38	1.09 - 1.75	1.42	1.08 - 1.86	0.011
Complete or incomplete tertiary	2.29	1.70 - 3.09	2.58	1.81 - 3.69	<0.001
**Ever had sex with a boy/man**					
No	1		1		
Yes	0.82	0.71 - 0.95	0.68	0.57- 0.82	<0.001
**Away from home in last 12 months**					
No	1		1		
Yes	1.15	0.80 - 1.64	1.21	0.83- 1.77	0.309

For peer review only - http://bmjopen.bmj.com/site/about/guidelines.xhtml

Relationship between MTV Shuga exposure and HIV and SRHR outcomes (Table 3&4)

In the nested cohorts by 2019, overall 63.3% of those aged 14-23 knew their HIV status, 13.4% were consistently using contraception, 20.0% were using condoms consistently, and about a third were aware of PrEP, 5% had a pregnancy and 15% acquired HSV-2. There were higher proportions of contraception use, condom use and PrEP awareness among those exposed to MTV Shuga (Table 3). For survey sites (Table 3) overall, 85.0% knew their HIV status, over a fifth 22.6% were using contraception and about half using condoms 48.4%. About a tenth (7.5%) were aware of PrEP, with higher proportions of these being among those exposed to MTV Shuga.

Table 3: HIV and SRHR outcomes by exposure to MTV Shuga nested cohort 13-22 year olds (n= 2167)

	Overall		Exposed (n=308)		Not exposed (n=1878)		P-value
	n/N	%	n/N	%	n/N	%	
Knowledge of HIV status, 2019	1083/1712	63.3	175/283	61.8	908/1429	63.5	0.587
Modern contraception, 2019	221/1651	13.4	56/271	20.7	165/1380	12	<0.001
Consistent condom use, 2019	168/838	20	41/141	29.1	127/697	18.2	0.003
Aware of PrEP, 2019	523/1712	30.5	124/283	43.8	399/1429	27.9	0.302
Pregnant in 2018/19	124/2184	5.7	20/308	6.5	104/1876	5.5	0.504
Teenage pregnancy	72/1395	5.16	55/1187	4.63	17/208	8.17	0.033
HSV-2 2018/19	241/1562	15.4	35/237	14.8	206/1325	15.5	0.760
**HIV and SRHR outcomes by exposure to MTV Shuga weighted for sampling cross sectional survey 12-24 year olds (n=4127)**							
	Overall		Exposed		Not exposed		
	n/N	%	n/N	%	n/N	%	p-value
PrEP awareness (N=4127)	310/4127	7.5	148/1477	10.0	162/2650	6.1	<0.001
HIV test (self-report) (N=2529)	2156/2529	85.3	656/797	82.3	1500/1732	86.6	0.005
Condom use at last sex							0.310
(N=1898)‡	918/1898	48.4	320/640	50.0	598/1258	47.5	
Contraception use (N=4127)	934/4127	22.6	302/1477	20.5	632/2650	23.9	0.012

‡Restricted to those who ever had sex with man

At follow up, incident HSV-2 and teenage pregnancy were high, HSV-2 incidence was 15.26 and teenage pregnancy incidence was 9.86 per 100 person-years, respectively (Table 4).

For peer review only

Table 4: Rate of SRHR outcome by exposure to MTV Shuga (nested cohort 13-22 year olds n=2167)

**HSV-2 incidence rate**					
	person-time	n with HSV-2	Rate/ 100 person-years (py)	[95% Conf.	Interval]
MTV Shuga Not exposed	1303.0	206	15.81	13.79	18.12
MTV Shuga Exposed	276.3	35	12.67	9.09	17.64
Total	1579.3	241	15.26	13.45	17.31
**Pregnancy incidence rates among all AGYW**					
	person-time	n pregnant	Rate/100 py	[95% Conf.	Interval]
MTV Shuga Not exposed	1068.7	104	9.73	8.03	11.79
MTV Shuga Exposed	188.5	20	10.61	6.84	16.44
Total	1257.2	124	9.86	8.27	11.76
**Pregnancy incidence rates among girls below 19 years**					
	person-time	n pregnant	Rate/100 py	[95% Conf.	Interval]
MTV Shuga Not exposed	668.7	55	8.22	6.31	10.71
MTV Shuga Exposed	123.9	17	13.72	8.53	22.07
Total	792.6	72	9.08	7.21	11.44

MTV Shuga and HIV prevention and SRHR awareness and uptake

In the nested cohort after adjusting for age, education, SES, area and DREAMS, MTV Shuga exposure in the AGYW cohort was associated with significantly greater awareness of PrEP (aOR=2.06, 95%CI: 1.57-2.70), contraception uptake (aOR=2.08, 95%CI: 1.45-2.98), and consistent condom use (aOR=1.84, 95%CI: 1.24-2.93). Watching MTV-Shuga was not associated with HIV testing (aOR=1.02,95%CI: 0.77-1.21) (Figure 3 and Supplementary Tables S1-S4). There was no effect modification by DREAMS exposure.

Figure 3 Forest plots showing the association between MTV Shuga exposure and HIV prevention and SRHR awareness and uptake in the nested cohorts.

Similarly, in the cross-sectional analysis, after adjusting for age, education, district, migration and sexual history, exposure to MTV-Shuga watching was associated with greater awareness of PrEP (aOR=1.70, 95%CI 1.20 - 2.43). However, there was no association with contraception, lower self-reported HIV testing, or condom use as shown in Figure 4 below (Supplementary Table 5).

For peer review only - http://bmjopen.bmj.com/site/about/guidelines.xhtml

Figure 4 Forest plots showing association between MTV Shuga exposure and HIV prevention and SRHR awareness and uptake in the cross-sectional surveys

Causal effect of MTV Shuga on health outcomes – HSV-2

The causal analysis similarly finds no effect of MTV Shuga on HSV-2, with a risk difference of 1.10 95% CI (-2.82- 5.38)%. Findings in the younger age group (aged 13-17) and the older age group (18-22) was similar (Supplementary Table 6).

Discussion

In our study of the population-level effect of a national broadcast of a TV-based edu-drama on HIV prevention and SRHR, exposure to MTV Shuga was associated with higher awareness of a novel HIV prevention intervention (PrEP). However, despite a very high incidence of HSV-2 and teenage pregnancy, MTV Shuga exposure was not significantly associated with safer sexual behaviour, uptake of contraception and HIV-testing or prevention of teenage pregnancy. Notably though, the size of the relationship and direction of effect between exposure to MTV Shuga, condom use and markers of unprotected sex (HSV-2 and pregnancy) was consistent with a possible relationship. These findings may be partly explained by our finding that less than one in 12 of the target age group had any exposure to MTV Shuga and only half of these had high exposure (defined as watching the MTV Shuga South African series and being able to recall the content), suggesting that one of the limiting factors for the effective use of TV-based edu-drama maybe the dose that young people are exposed to, particularly in rural and resource-constrained settings most affected by HIV.

Our inability to find a measurable population effect of mass-media edu-drama behaviour change campaign compared to the trial findings of the randomised controlled trial is disappointing (18). However, firstly the direction and size of the relationships suggest that we may have been able to see an effect if the proportion exposed had been greater than 7%. Secondly, AGYW in urban settings were more likely to have been exposed to MTV Shuga and they are also more likely to be living in small towns and townships. Data from our settings suggest that young people in small towns and townships are more vulnerable to HIV (28) and sexual risk (29) and therefore it is possible that a real effect of MTV Shuga on this group was masked by their greater risk for the outcome. Due to the low numbers with high levels of exposure to MTV Shuga we did not have the power to explore this hypothesis by looking at effect modification by geography or socioeconomic status.

For peer review only - http://bmjopen.bmj.com/site/about/guidelines.xhtml

For peer review only

The finding of a differential association of MTV Shuga exposure with awareness of PrEP, compared to uptake of HIV and contraception, suggests that whilst educational mass entertainment may be able to increase awareness and possibly demand for a service, it does not impact on accessibility of the service: i.e. it impacts the first two steps of the prevention cascade and not the final step(30). Well described barriers to uptake of HIV-testing and contraception in this area are internalized and externalized stigma, fear of judgement from health care workers and the social costs of accessing care in busy primary health care settings(31-33). Behaviour change intervention including mass communication campaigns can be constrained or facilitated by the context in which people live (17, 34). To optimise MTV Shuga's effect there may need to be parallel innovations in SRHR and HIV service delivery that makes the services easier to access. We aim to test this hypothesis by providing community-based delivery of HIV and SRHR services in the context of the MTV Shuga.

The behaviour change theory that underpins edu-drama as a vehicle for mass behaviour change communication (16) explicitly suggests that the audience, and especially the early adopters, need to be actively watching, rather than passively watching or listening (17, 18). TV watching in rural homesteads can be in the context of large, often grandparent-led households and competing chores and priorities. This coupled with the relatively late timing of the shows may explain why so few girls and young women were sufficiently engaged or immersed to be able to recall characters or story lines. Moreover, the timing of this analysis may have allowed insufficient time for early adopters to convey the message of the show.

Limitations

The limitations of our study are that a meaningful exposure to MTV Shuga was low and so whilst the size of the relationship and direction of effect between exposure to MTV Shuga, condom use, and markers of unprotected sex (HSV-2 and pregnancy) suggested a possible effect, but we did not have the power to show a significant relationship between exposure to MTV Shuga and SRHR outcomes. We also do not have the power to see a difference by dose and immersion. Furthermore, as this is an observational study, we cannot exclude the possibility that those who are exposed to MTV Shuga are systematically different in ways that impact on the outcome of interest, for example more exposed to social media and sexual health promotion and innovative technologies to support sexual health than those who were not.

For peer review only - http://bmjopen.bmj.com/site/about/guidelines.xhtml

Conclusions and implications for the future

- Meaningful exposure to MTV Shuga was low across rural and urban settings in South Africa and so additional efforts need to be made to reach young people and increase their immersion in promising edu-drama if it is to have the desired effect, especially in rural and deprived settings.
- MTV Shuga was an effective vehicle to raise awareness and promote newer HIV prevention technologies such as HIV PrEP.
- There was some suggestion that MTV Shuga improved uptake of some HIV prevention and sexual health technologies (contraception and condoms)
- There was less evidence from this observational study that it improved SRHR and HIV outcomes

We highlight the importance of evaluating the real-world scale up of promising interventions to understand both the reach and population effect as well as inform interventions to increase impact and equity.

Efforts to increase exposure, which have been rolled out as part of MTV Shuga in SA, such as social media, school-based or community-based MTV Shuga film clubs will need to be evaluated, both to understand whether or not they increase exposure and coverage and improve SRH and HIV outcomes. However, to have a significant impact on the HIV and SRH prevention and treatment cascades, demand generation in AGYW needs to be delivered in parallel with accessible service delivery models that support adherence and retention (30).

Contributorship

M.S., N.C., J.D., T.Z. N.K, G.H. and C.C. developed the study tools and performed the research. M.S., J.S. D.P., K.B., I.B., and S.F. designed the research study. T.S. and S.D. conducted the laboratory analysis. N.M. supported by K.B. conducted the statistical analysis and G.C. M.S wrote the first and final draft of the paper with input from N.C., N.M., G.H., J.S., I.B., G.C., K.B., C.C., T.S., T.Z, D.P and N.K. All the authors critically reviewed the manuscript. All authors have approved the final draft of the paper.

Competing interests

All authors declare they have no conflict of interest

For peer review only

Funding

The impact evaluation of DREAMS and MTV Shuga is funded by the Bill and Melinda Gates Foundation (OPP1136774 and OPP1171600, http://www.gatesfoundation.org). Foundation staff advised the study team, but did not substantively affect the study design, instruments, interpretation of data, or decision to publish. The research leading to these results has received funding from the People Programme (Marie Curie Actions) of the European Union's seventh Framework Programme FP7/2007-2013 under REA grant agreement n° 612216. MS National Institutes of Health 5R01MH114560-03 funding acknowledgement. This research was funded in whole, or in part, by the Africa Health Research Institute through the Wellcome [Strategic Core award: 201433/Z/16/A]. For the purpose of open access, the author has applied a CC BY public copyright licence to any Author Accepted Manuscript version arising from this submission. N.C is supported by a training fellowship from the National Institute for Health Research (NIHR) (using the UK's Official Development Assistance (ODA) Funding) and Wellcome [grant reference number 224309/Z/21/Z] under the NIHR-Wellcome Partnership for Global Health Research. The views expressed are those of the authors and not necessarily those of Wellcome, the NIHR or the Department of Health and Social Care. GH is supported by a fellowship from the Royal Society and the Wellcome Trust [Grant number 210479/Z/18/Z]. MS work is also supported by a BMGF 3ie grant at AHRI. This research was funded in whole, or in part, by the Wellcome Trust [Grant number 210479/Z/18/Z]. For the purpose of open access, the author has applied a CC BY public copyright licence to any Author Accepted Manuscript version arising from this submission.

Data sharing statement

Data are available on reasonable request.

Ethics statement

The DREAMS Partnership impact evaluation protocol was reviewed and approved by the University of KwaZulu-Natal (UKZN) Biomedical Research Ethics Committee (BREC) (BFC 339/16), the London School of Hygiene & Tropical Medicine Research Ethics Committee (Ref 11835) and the AHRI Somkhele Community Advisory Board, the Associate Director of Science of the Center for Global Health (CGH) at the United States Centers for Disease Control and Prevention (CDC) in Atlanta and the Department of Health, Province of KZN and Gauteng. Approval for the demographic surveillance and data collection in the clinics was granted by UKZN BREC. Participation in the study was voluntary. All participants provided separate informed consent to take part in data collection through the questionnaires and the HSV2 sero-

For peer review only

survey. Consent for follow-up interviews was provided separately. For participants aged <18 years, written parental consent and participant assent were provided. All data were anonymised for analysis.

Acknowledgements

The authors are grateful to the communities of the Hlabisa sub-district, Ekurhuleni district, Thekwini district and City of Johannesburg who contributed their data to this study and to all the staff at Africa Health Research Institute and EpiCentre who collected the data. We acknowledge Eskindir Shambullo for his input in this manuscript.

References

1. HSRC. The fifth South African National HIV prevalence, incidence, behaviour and communication survey [Report]. http://www.hsrc.ac.za/uploads/pageContent/9234/SABSSMV_Impact_Assessment_Summary_ZA_ADS_cleared_PDFA4.pdf: South African Health Services Research Council 2017 [updated 01/11/2018.
2. Chimbindi N, Mthiyane N, Birdthistle I, Floyd S, McGrath N, Pillay D, et al. Persistently high incidence of HIV and poor service uptake in adolescent girls and young women in rural KwaZulu-Natal, South Africa prior to DREAMS. PLoS One. 2018;13(10):e0203193.
3. Vandormael A, Akullian A, Siedner M, de Oliveira T, Bärnighausen T, Tanser F. Declines in HIV incidence among men and women in a South African population-based cohort. Nature Communications. 2019;10(1):5482.
4. Birdthistle I, Kwaro D, Shahmanesh M, Baisley K, Khagayi S, Chimbindi N, et al. Evaluating the impact of DREAMS on HIV incidence among adolescent girls and young women: A population-based cohort study in Kenya and South Africa. PLoS medicine. 2021;18(10):e1003837.
5. Department of Health RoSA. National Campaign for Young Women and Adolescent Girls. . 2016.
6. Saul J, Bachman G, Allen S, Toiv NF, Cooney C, Beamon TA. The DREAMS core package of interventions: A comprehensive approach to preventing HIV among adolescent girls and young women. PLOS ONE. 2018;13(12):e0208167.
7. PEPFAR. Preventing HIV in Adolescent Girls and Young Women: Guidance for PEPFAR Country Teams on the DREAMS Partnership 2015.
8. PEPFAR. South Africa DREAMS overview https://www.pepfar.gov/documents/organization/253958.pdf: PEPFAR; 2017 [

For peer review only - http://bmjopen.bmj.com/site/about/guidelines.xhtml

9. Chang L, Serwadda D, Quinn T, Wawer M, Gray R, Reynolds S. Combination implementation for HIV prevention: moving from clinical trial evidence to population-level effects The Lancet Infectious Diseases 2013;13(1):65-76.

10. Iwuji CC, Orne-Gliemann J, Larmarange J, Balestre E, Thiebaut R, Tanser F, et al. Universal test and treat and the HIV epidemic in rural South Africa: a phase 4, open-label, community cluster randomised trial. Lancet HIV. 2018;5(3):e116-e25.

11. Iwuji CC, Orne-Gliemann J, Tanser F, Boyer S, Lessells RJ, Lert F, et al. Evaluation of the impact of immediate versus WHO recommendations-guided antiretroviral therapy initiation on HIV incidence: the ANRS 12249 TasP (Treatment as Prevention) trial in Hlabisa sub-district, KwaZulu-Natal, South Africa: study protocol for a cluster randomised controlled trial. Trials. 2013;14:230.

12. Birdthistle I, Schaffnit SB, Kwaro D, Shahmanesh M, Ziraba A, Kabiru CW, et al. Evaluating the impact of the DREAMS partnership to reduce HIV incidence among adolescent girls and young women in four settings: a study protocol. BMC Public Health. 2018;18(1):912.

13. Shen F, Han J. Effectiveness of entertainment education in communicating health information: a systematic review. Asian Journal of Communication. 2014;24(6):605-16.

14. Noar SM, Palmgreen P, Chabot M, Dobransky N, Zimmerman RS. A 10-year systematic review of HIV/AIDS mass communication campaigns: have we made progress? Journal of Health Communication. 2009;14(1):15-42.

15. Kyegombe N, Zuma T, Hlongwane S, Nhlenyama M, Chimbindi N, Birdthistle I, et al. A qualitative exploration of the salience of MTV-Shuga, an edutainment programme, and adolescents' engagement with sexual and reproductive health information in rural KwaZulu-Natal, South Africa. Sexual and Reproductive Health Matters. 2022;30(1):2083809.

16. Barker K. Sex Soap and Social change- The Sabedo methadology https://www.populationmedia.org/product/sabido-theory/; http://www.comminit.com/content/sabido-methodology [

17. Baker V, Birdthistle I, Seeley J, Arnold G, Piot S, Hajiyiannis H. How can a TV drama about Love, Sex and Growing up Increase Knowledge and Shape the Sexual Health Behaviours of Young People? . AIDS Impact; Cape Town, South Africa2017.

18. MTV Shuga Soap Opera Turns Edutainment into a Tool to Fight HIV and Gender-Based Violence http://www.worldbank.org/en/news/feature/2017/02/23/mtv-shuga-soap-opera-turns-edutainment-into-a-tool-to-fight-hiv-and-gender-based-violence2017 [

For peer review only - http://bmjopen.bmj.com/site/about/guidelines.xhtml

19. National Department of Health. The National Antenatal Sentinel HIV prevalence survey, South Africa, http://www.health.gov.za/index.php/shortcodes/2015-03-29-10-42-47/2015-04-30-08-18-10/2015-04-30-08-21-56?download=2584:2015-national-antenatal-hiv-prevalence-survey-final-23oct17, 2013 [

20. Herbst K, Law M, Geldsetzer P, Tanser F, Harling G, Barnighausen T. Innovations in health and demographic surveillance systems to establish the causal impacts of HIV policies. Current Opinion on HIV AIDS. 2015;10(6):483-94.

21. Gareta D, Baisley K, Mngomezulu T, Smit T, Khoza T, Nxumalo S, et al. Cohort Profile Update: Africa Centre Demographic Information System (ACDIS) and population-based HIV survey. Int J Epidemiol. 2021;50(1):33-4.

22. George G, Cawood C, Puren A, Khanyile D, Gerritsen A, Govender K, et al. Evaluating DREAMS HIV prevention interventions targeting adolescent girls and young women in high HIV prevalence districts in South Africa: protocol for a cross-sectional study. BMC Women's Health. 2020;20(1):7.

23. Filmer D, Pritchett LH. Estimating Wealth Effects Without Expenditure Data—Or Tears: An Application To Educational Enrollments In States Of India*. Demography. 2001;38:115-32.

24. Arvin A, Prober C. Herpes Simplex Viruses. In: Murray P, Baron E, Pfaller M, Tenover F, Yolkenet R, editors. Manual of Clinical Microbiology 6th Ed Washington D.C.: ASM; 1995. p. 876-83.

25. Delany-Moretlwe S, Jentsch U, Weiss H, Moyes J, Ashley-Morrow R, Stevens W, et al. Comparison of Focus HerpeSelect ® and Kalon TM HSV-2 gG2 ELISA serological assays to detect herpes simplex virus type 2 (HSV-2) antibodies in a South African population. . Sexually Transmitted Infections,. 2009;86(1):46-50.

26. Ashley-Morrow R, Nolkamper J, Robinson N, Bishop N, Smith J. Performance of Focus ELISA test for herpes simplex virus type 1 (HSV-1) and HSV-2 antibodies among women in ten diverse geographical locations. . Clinical Microbiology and Infection 2004;10(6):530-6.

27. STROBE Statement. STROBE Checklists 2007 [Available from: http://www.strobe-statement.org/index.php?id=available-checklists.

28. Cuadros DF, Li J, Branscum AJ, Akullian A, Jia P, Mziray EN, et al. Mapping the spatial variability of HIV infection in Sub-Saharan Africa: Effective information for localized HIV prevention and control. Scientific Reports. 2017;7(1):9093.

29. Ngwenya N, Gumede D, Shahmanesh M, McGrath N, Grant A, Seeley J. Community perceptions of the socio-economic structural context influencing HIV and TB risk, prevention and treatment in a high prevalence area in the era of antiretroviral therapy. African Journal of AIDS Research. 2018;17(1):72-81.

For peer review only - http://bmjopen.bmj.com/site/about/guidelines.xhtml

30. Hargreaves JR, Delany-Moretlwe S, Hallett TB, Johnson S, Kapiga S, Bhattacharjee P, et al. The HIV prevention cascade: integrating theories of epidemiological, behavioural, and social science into programme design and monitoring. Lancet HIV. 2016;3(7):e318-22.

31. Hargreaves JR, Krishnaratne S, Mathema H, Lilleston PS, Sievwright K, Mandla N, et al. Individual and community-level risk factors for HIV stigma in 21 Zambian and South African communities: analysis of data from the HPTN071 (PopART) study. AIDS. 2018;32(6):783-93.

32. Chimbindi N, Zuma T, Nakasone S, Birdthistle I, Mthiyane N, Dreyer J, et al. Platforms, Processes and Perceptions of PrEP Delivery Through DREAMS in a Rural Setting in KwaZulu-Natal, South Africa. . Research for Prevention 2018 (HIVR4P 2018) Madrid, Spain 2018.

33. Adeagbo O, Herbst C, Dlamini N, Mhlongo T, Luthuli M, Xulu S, et al. “If she tests negative, it means I am also negative”: Men’s construction of HIV testing in KwaZulu-Natal, South Africa. . HIV Research for Prevention (HIVR4P) 23 October 2018.; Madrid, Spain **2018**

34. Michie S, van Stralen MM, West R. The behaviour change wheel: A new method for characterising and designing behaviour change interventions. Implementation Science. 2011;6(42).

For peer review only - http://bmjopen.bmj.com/site/about/guidelines.xhtml

Figure legends

Figure 1 showing conceptual framework for MTV Shuga impact on HIV prevention on AGYW in uMkhanyakude

Figure 2 Flowchart showing AGYW follow-up from nested cohorts 2017-2019

Figure 3 Forest plots showing the association between MTV Shuga exposure and HIV prevention and SRHR awareness and uptake in the nested cohorts.

Figure 4 Forest plots showing association between MTV Shuga exposure and HIV prevention and SRHR awareness and uptake in the cross-sectional surveys

For peer review only

For peer review only - http://bmjopen.bmj.com/site/about/guidelines.xhtml

Figure 1: Conceptual framework for MTV Shuga impact on HIV prevention in uMkhanyakude

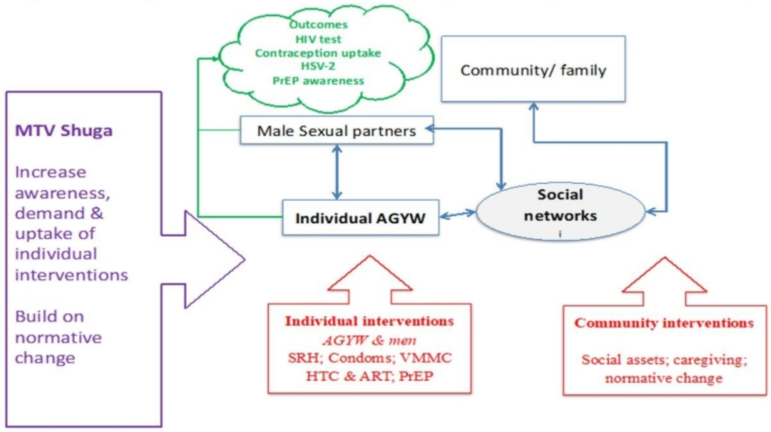

Figure 1 showing conceptual framework for MTV Shuga impact on HIV prevention on AGYW in uMkhanyakude

98x51mm (400 x 400 DPI)

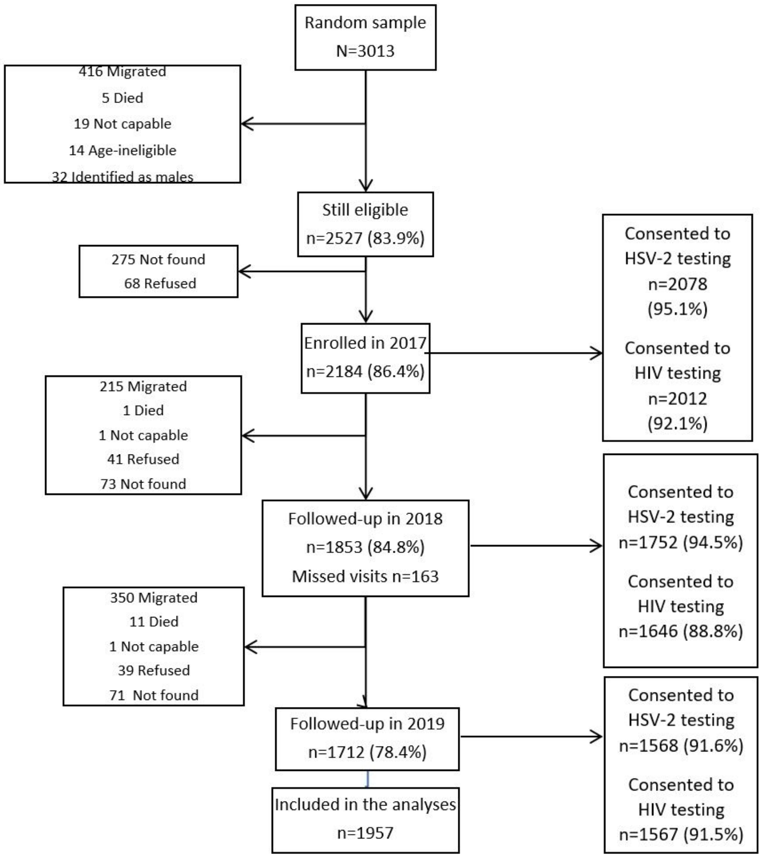

Figure 2 Flowchart showing AGYW follow-up from nested cohorts 2017-2019

71x64mm (400 x 400 DPI)

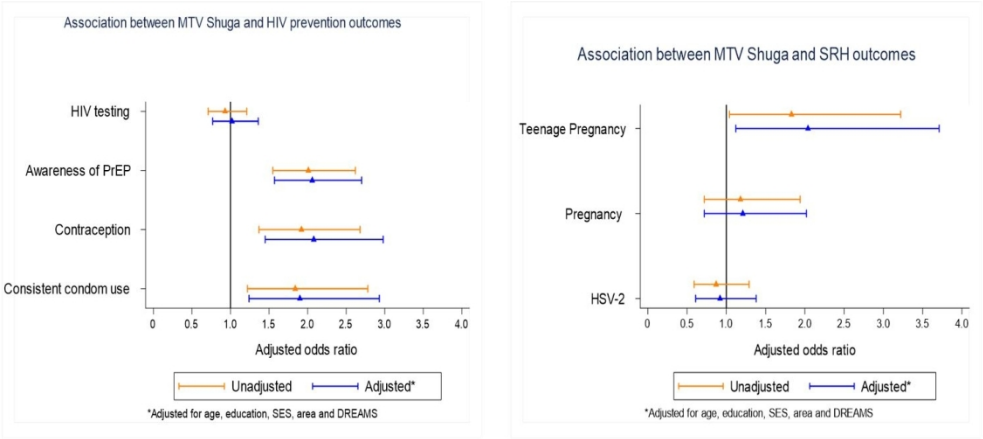

Figure 3 Forest plots showing the association between MTV Shuga exposure and HIV prevention and SRHR awareness and uptake in the nested cohorts.

95x46mm (400 x 400 DPI)

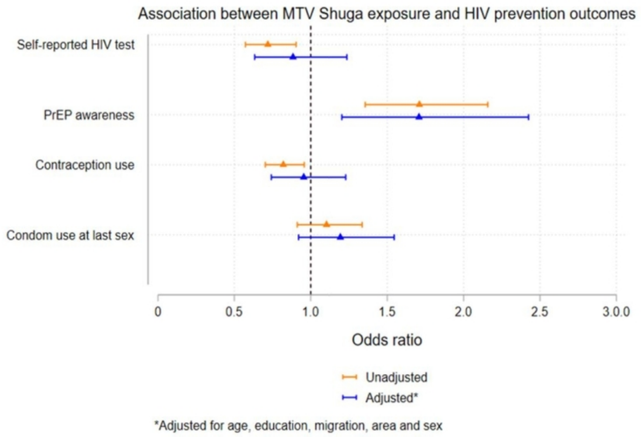

Figure 4 Forest plots showing association between MTV Shuga exposure and HIV prevention and SRHR awareness and uptake in the cross-sectional surveys

101x54mm (400 x 400 DPI)

Supplementary Table 1 Exposure to MTV Shuga and PrEP awareness in the nested cohort of AGYW aged 13-22 (n=2184)

	Unadjusted		Shuga adjusted			Adjusted-All		
	**OR**	**95%CI**	**OR**	**95%CI**	**p-value**	**OR**	**95%CI**	**p-value**
**Ever watched MTV Shuga, 2018/19**								
No	1					1		
Yes	2.01	1.55 -2.62				2.06	1.57 -2.70	<0.001
**Age**	1.12	1.08-1.16	1.12	1.08-1.17	<0.001	1.11	1.06-1.17	<0.001
**Currently in school**								
No	1		1			1		
Yes	0.6	0.47 -0.76	0.59	0.46 -0.75	<0.001	0.89	0.65 -1.23	0.488
**Socio-economic status, 2018**								
Low	1		1			1		
Middle	0.99	0.71 -1.39	0.96	0.68 -1.35		1.01	0.71 -1.44	
High	1.13	0.81 -1.58	1.05	0.75 -1.47	0.744	1.11	0.78 -1.58	0.699
**Urban or rural**								
Rural	1		1			1		
Peri-urban/urban	0.98	0.79 -1.22	0.93	0.75 -1.16	0.525	0.92	0.73 -1.15	0.459
**Invited or received DREAMS, 2017/18**								
No	1		1			1		
Yes	0.89	0.73 -1.10	0.88	0.71 -1.08	0.212	0.99	0.79 -1.23	0.897

For peer review only

Supplementary Table 2 Exposure to MTV Shuga and contraception uptake in the nested cohort of AGYW aged 13-22 (n=2184)

	Unadjusted		Shuga adjusted			Adjusted-All		
	OR	95%CI	OR	95%CI	p-value	OR	95%CI	p-value
**Ever watched MTV Shuga, 2018/19**								
No	1					1		
Yes	1.92	1.37 -2.68				2.08	1.45-2.98	<0.001
**Age**	1.26	1.19-1.33	1.27	1.20-1.34	<0.001	1.32	1.24-1.42	<0.001
**Currently in school**								
No	1		1			1		
Yes	0.6	0.44 -0.83	0.59	0.43-0.82	0.001	1.7	1.13-2.55	0.01
**Socio-economic status, 2018**								
Low	1		1			1		
Middle	0.79	0.51 -1.22	0.76	0.49-1.19		0.8	0.51-1.28	
High	0.86	0.55 -1.34	0.8	0.51-1.24	0.489	0.83	0.52-1.32	0.646
**Urban or rural**								
Rural	1		1			1		
Peri-urban/urban	0.91	0.68 -1.24	0.86	0.64-1.17	0.345	0.89	0.64-1.24	0.49
**Invited or received DREAMS, 2017/18**								
No	1		1			1		
Yes	0.84	0.63 -1.11	0.83	0.62-1.10	0.191	1.04	0.76-1.42	0.813

For peer review only

Supplementary Table 3 Exposure to MTV Shuga and consistent condom use in the nested cohort of AGYW aged 13-22 (n=2184)

	Unadjusted		Shuga adjusted			Adjusted-All		
	OR	95%CI	OR	95%CI	p-value	OR	95%CI	p-value
**Ever watched MTV Shuga, 2018/19**								
No	1					1		
Yes	1.84	1.22-2.78				1.9	1.24-2.93	0.003
**Age**	0.94	0.86-1.01	0.94	0.87-1.02	0.157	1	0.91-1.10	0.984
**Currently in school**								
No	1		1			1		
Yes	1.76	1.22-2.53	1.73	1.20-2.49	0.003	1.78	1.15-2.76	0.01
**Socio-economic status, 2018**								
Low	1		1			1		
Middle	1.01	0.60-1.70	0.97	0.58-1.63		0.98	0.58-1.67	
High	0.87	0.52-1.47	0.8	0.47-1.36	0.552	0.86	0.50-1.48	0.753
**Urban or rural**								
Rural	1		1			1		
Peri-urban/urban	0.7	0.48-1.02	0.67	0.46-0.97	0.035	0.71	0.48-1.05	0.088
**Invited or received DREAMS, 2017/18**								
No	1		1			1		
Yes	1.13	0.81-1.59	1.13	0.80-1.59	0.486	0.97	0.67-1.40	0.875

For peer review only

Supplementary Table 4 Exposure to MTV Shuga and HIV testing in the nested cohort of AGYW aged 13-22 (n=2184)

	Unadjusted		Shuga adjusted			Adjusted-All		
	OR	95%CI	OR	95%CI	p-value	OR	95%CI	p-value
**Ever watched MTV Shuga, 2018/19**								
No	1					1		
Yes	0.93	0.71-1.21				1.02	0.77-1.36	0.885
**Age**	1.3	1.25-1.35	1.3	1.24-1.35	<0.001	1.3	1.24-1.37	<0.001
**Currently in school**								
No	1		1			1		
Yes	0.37	0.28-0.48	0.37	0.28-0.48	0	1.07	0.74-1.53	0.726
**Socio-economic status, 2018**								
Low	1		1			1		
Middle	0.67	0.48-0.94	0.67	0.48-0.95		0.71	0.49-1.02	
High	0.58	0.41-0.81	0.58	0.41-0.81	0.007	0.6	0.41-0.86	0.018
**Urban or rural**								
Rural	1		1			1		
Peri-urban/urban	0.84	0.68-1.03	0.84	0.69-1.04	0.104	0.89	0.71-1.11	0.298
**Invited or received DREAMS, 2017/18**								
No	1		1			1		
Yes	0.84	0.69-1.02	0.84	0.69-1.02	0.081	0.98	0.79-1.23	0.89

For peer review only

Supplementary Table 5: Exposure to MTV Shuga with HIV prevention and SRHR awareness and uptake in the cross-sectional analysis of AGYW aged 12-24 (n=4127)

	aOR (95% Confidence Interval)	P-value
**District**		
City of Johannesburg	1	
Ekurhuleni	0.66 (0.47-0.91)	0.013
eThekwini	0.78 (0.54-1.13)	0.186
**Age group**		
12-14	1	
15-19	1.75 (0.35-8.84)	0.499
20-24	1.60 (0.32-8.07)	0.570
**Highest education level**		
Grade R to 7	1	
No schooling	3.84 (1.05-14.02)	0.041
Grade 8 to 12	3.17 (0.93-10.77)	0.065
Complete or incomplete tertiary	6.52 (1.90-22.33)	0.003
**Ever had sex with a boy/man**		
No	1	
Yes	0.67 (0.32-1.40)	0.286
**Away from home**		
No	1	
Yes	1.48 (0.93-2.37)	0.099
**PrEP awareness**		
No	1	
Yes	1.71 (1.20-2.43)	0.003
**Contraception use**		
No	1	
Yes	0.95 (0.74-1.23)	0.718
**Condom use at last sex**		
No	1	
Yes	1.19 (0.92-1.55)	0.183
**HIV test (self-report)**		
No	1	
Yes	0.88 (0.63-1.24)	0.471

For peer review only

Supplementary Table 6 Estimated causal effect of MTV Shuga on HSV-2 incidence, overall and by age group

**Estimated causal effect of MTV Shuga on HSV-2 incidence, overall and by age group**											
	**% Outcome in total study population**	**Estimated % Outcome if no Shuga**	**95% CI**	**Estimated % Outcome if all get Shuga**	**95% CI**	**Risk Difference (%; PS adjusted)**	**95% CI**	**Prevalence Ratio (%; PS adjusted)**	**95% CI**	**Odds Ratio (%; PS adjusted)**	**95% CI**
**PS adjustment: Primary results**											
Overall	17.3	17.2	14.71 - 19.67	18.0	14.51 - 22.32	0.80	-3.24 - 5.71	1.05	0.82 - 1.36	1.06	0.79 - 1.46
13-17 Years	13.2	12.4	9.85 - 15.16	15.7	11.63 - 20.26	3.34	-1.60 - 8.25	1.27	0.89 - 1.79	1.32	0.87 - 1.98
18-22 Years	24.3	25.8	21.01 - 30.74	22.0	15.19 - 30.17	-3.76	-11.92 - 5.68	0.85	0.59 - 1.24	0.81	0.51 - 1.34
**Sensitivity analyses**											
Regression under counterfactual framework											
Overall	17.3	17.2	14.74 - 19.66	17.8	14.50 - 22.08	0.65	-3.42 - 5.72	1.04	0.82 - 1.36	1.05	0.78 - 1.46
13-17 Years	13.2	12.42	9.86 - 15.21	15.9	11.85 - 20.49	3.47	-1.54 - 8.63	1.28	0.89 - 1.80	1.33	0.88 - 1.99
18-22 Years	24.3	25.7	20.78 - 30.63	21.3	14.88 - 28.28	-4.42	-12.28 - 4.95	0.83	0.57 - 1.22	0.78	0.48 - 1.31
PS stratification											
Overall	17.3	17.1	14.68 - 19.68	17.9	14.53 - 22.13	0.80	-3.21 - 5.52	1.09	0.84 - 1.40	1.11	0.82 - 1.51
PS weighting											

For peer review only

Overall	17.3	17.08	14.74 - 19.48	17.22	13.99 - 21.10	0.13	-3.76 - 4.63	1.01	0.79 - 1.30	1.01	0.76 - 1.37

For peer review only

STROBE Statement—checklist of items that should be included in reports of observational studies

	Item No	Recommendation	Page No
**Title and abstract**	1	(*a*) Indicate the study's design with a commonly used term in the title or the abstract	1
		(*b*) Provide in the abstract an informative and balanced summary of what was done and what was found	2
**Introduction**			
Background/rationale	2	Explain the scientific background and rationale for the investigation being reported	4
Objectives	3	State specific objectives, including any prespecified hypotheses	6
**Methods**			
Study design	4	Present key elements of study design early in the paper	6-7
Setting	5	Describe the setting, locations, and relevant dates, including periods of recruitment, exposure, follow-up, and data collection	7
Participants	6	(*a*) *Cohort study*—Give the eligibility criteria, and the sources and methods of selection of participants. Describe methods of follow-up<br>*Case-control study*—Give the eligibility criteria, and the sources and methods of case ascertainment and control selection. Give the rationale for the choice of cases and controls<br>*Cross-sectional study*—Give the eligibility criteria, and the sources and methods of selection of participants	7
		(*b*) *Cohort study*—For matched studies, give matching criteria and number of exposed and unexposed<br>*Case-control study*—For matched studies, give matching criteria and the number of controls per case	
Variables	7	Clearly define all outcomes, exposures, predictors, potential confounders, and effect modifiers. Give diagnostic criteria, if applicable	8-9
Data sources/ measurement	8*	For each variable of interest, give sources of data and details of methods of assessment (measurement). Describe comparability of assessment methods if there is more than one group	8-10
Bias	9	Describe any efforts to address potential sources of bias	9-10
Study size	10	Explain how the study size was arrived at	6-7
Quantitative variables	11	Explain how quantitative variables were handled in the analyses. If applicable, describe which groupings were chosen and why	8
Statistical methods	12	(*a*) Describe all statistical methods, including those used to control for confounding	9-10
		(*b*) Describe any methods used to examine subgroups and interactions	10
		(*c*) Explain how missing data were addressed	10
		(*d*) *Cohort study*—If applicable, explain how loss to follow-up was addressed<br>*Case-control study*—If applicable, explain how matching of cases and controls was addressed<br>*Cross-sectional study*—If applicable, describe analytical methods taking account of sampling strategy	10
		(*e*) Describe any sensitivity analyses	10

Continued on next page

For peer review only - http://bmjopen.bmj.com/site/about/guidelines.xhtml

**Results**			
Participants	13*	(a) Report numbers of individuals at each stage of study—eg numbers potentially eligible, examined for eligibility, confirmed eligible, included in the study, completing follow-up, and analysed	11
		(b) Give reasons for non-participation at each stage	11
		(c) Consider use of a flow diagram	11
Descriptive data	14*	(a) Give characteristics of study participants (eg demographic, clinical, social) and information on exposures and potential confounders	11
		(b) Indicate number of participants with missing data for each variable of interest	
		(c) *Cohort study*—Summarise follow-up time (eg, average and total amount)	
Outcome data	15*	*Cohort study*—Report numbers of outcome events or summary measures over time	12
		Case-control study—Report numbers in each exposure category, or summary measures of exposure	
		Cross-sectional study—Report numbers of outcome events or summary measures	12
Main results	16	(*a*) Give unadjusted estimates and, if applicable, confounder-adjusted estimates and their precision (eg, 95% confidence interval). Make clear which confounders were adjusted for and why they were included	12-16
		(*b*) Report category boundaries when continuous variables were categorized	12-16
		(*c*) If relevant, consider translating estimates of relative risk into absolute risk for a meaningful time period	
Other analyses	17	Report other analyses done—eg analyses of subgroups and interactions, and sensitivity analyses	17
**Discussion**			
Key results	18	Summarise key results with reference to study objectives	17-18
Limitations	19	Discuss limitations of the study, taking into account sources of potential bias or imprecision. Discuss both direction and magnitude of any potential bias	18
Interpretation	20	Give a cautious overall interpretation of results considering objectives, limitations, multiplicity of analyses, results from similar studies, and other relevant evidence	18-19
Generalisability	21	Discuss the generalisability (external validity) of the study results	17-19
**Other information**			
Funding	22	Give the source of funding and the role of the funders for the present study and, if applicable, for the original study on which the present article is based	20

*Give information separately for cases and controls in case-control studies and, if applicable, for exposed and unexposed groups in cohort and cross-sectional studies.

Note: An Explanation and Elaboration article discusses each checklist item and gives methodological background and published examples of transparent reporting. The STROBE checklist is best used in conjunction with this article (freely available on the Web sites of PLoS Medicine at http://www.plosmedicine.org/, Annals of Internal Medicine at http://www.annals.org/, and Epidemiology at http://www.epidem.com/). Information on the STROBE Initiative is available at www.strobe-statement.org.

For peer review only
